# *Rubus occidentalis* Ethanol Extract Attenuates Neuroinflammation and Cognitive Impairment in Lipopolysaccharide-Stimulated Microglia and Scopolamine-Induced Amnesic Mice

**DOI:** 10.3390/ph18101557

**Published:** 2025-10-16

**Authors:** Ga-Won Kim, Yon-Suk Kim, Tohmina Afroze Bondhon, Rengasamy Balakrishnan, Jun-Hyuk Han, Ji-Wung Kwon, Woo-Jung Kim, Dong-Kug Choi

**Affiliations:** 1 Department of Applied Life Sciences, Graduate School, BK21 Program, Konkuk University, Chungju 27478, Republic of Korea; gawon7425@naver.com (G.-W.K.); afrozebondhon@gmail.com (T.A.B.); digit0516@naver.com (J.-H.H.); 2 Department of Biotechnology, College of Biomedical and Health Science, Research Institute of Inflammatory Disease (RID), Konkuk University, Chungju 27478, Republic of Korea; kimyonsuk@kku.ac.kr (Y.-S.K.); balakonkuk@kku.ac.kr (R.B.); 3 Gochang Food & Industry Institute, Gochang 56417, Republic of Korea; kjwung@hanmail.net; 4 Biocenter, Gyeonggido Business and Science Accelerator, Gwanggyo-ro 147, Yeongtong-gu, Suwon 16229, Republic of Korea; wj0504@gbsa.or.kr

**Keywords:** *Rubus occidentalis*, neuroinflammation, lipopolysaccharide, cognitive function, miquelianin

## Abstract

**Background/Objectives:** Neuroinflammatory mechanisms, primarily mediated by activated microglia, play a key role in the progression of conditions such as mild cognitive impairment associated with Alzheimer’s disease. *Rubus occidentalis* (*R. occidentalis*), a black-fruited raspberry native to North America, is reported to possess antimicrobial, antidiabetic, and anticancer properties. This study investigated the neuroprotective and anti-neuroinflammatory effects of a 100% ethanol extract from premature *R. occidentalis* fruits (ROE) in lipopolysaccharide (LPS)-stimulated BV-2 microglial cells and a scopolamine-induced amnesic mouse model. **Methods:** C57BL/6N mice were orally administered ROE (100 or 200 mg/kg/b.w.) and donepezil (DNZ, 5 mg/kg) for 9 days and intraperitoneally injected with scopolamine (2 mg/kg/b.w.) for two days. Spatial learning and cognitive function were assessed using the Y-maze and Morris water maze tests. Protein and mRNA levels were examined both in vitro and in vivo through Western blotting and RT-PCR analysis. **Results:** In vitro, ROE improved cell viability and reduced nitric oxide overproduction in LPS-stimulated BV-2 cells, attenuated LPS-induced phosphorylation and degradation of IκB-α (thereby limiting NF-κB p65 nuclear translocation), and suppressed phosphorylation of MAPK signaling components. In vivo, ROE administration enhanced spatial learning and memory in scopolamine-treated C57BL/6N mice, increased hippocampal levels of brain-derived neurotrophic factor (BDNF) and phosphorylated CREB, and reduced the expression of iNOS and COX-2. **Conclusions:** Collectively, these results suggest that ROE possesses neuroprotective properties mediated by inhibition of NF-κB and MAPK signaling, promotion of CREB/BDNF pathways, and amelioration of neuroinflammation and cognitive deficits. Thus, ROE may represent a promising therapeutic candidate for neuroinflammatory disorders.

## 1. Introduction

Excessive microglia-mediated inflammation in the central nervous system (CNS) is a key feature of early mild cognitive impairment (MCI) linked to Alzheimer’s disease (AD). Aging is the leading risk factor, and the global population of individuals aged 65 and older with AD is expected to rise from 6.8 million in 2020 to 13.8 million by 2050 [1]. Clinically, AD is characterized by cognitive decline (including gradual memory loss, language impairments, and reduced ability to perform daily activities) and behavioral disturbances [2]. Neuropathological hallmarks include the accumulation of amyloid-beta plaques, tau hyperphosphorylation, neurofibrillary tangle formation, mitochondrial dysfunction, neuroinflammation, and neuronal loss [3]. In recent years, neuroinflammation has emerged as a major focus in AD research, with microglia recognized as key contributors. While microglial activation and associated pro-inflammatory phenotypes can confer neuroprotection, chronic activation leads to sustained neuroinflammation, a core pathological feature of AD and other neurodegenerative disorders [4].

Scopolamine, a non-selective muscarinic receptor antagonist, impairs spatial learning and short-term memory in both rodents and humans [5]. Scopolamine-induced mouse models are commonly used in neurocognitive research to reproduce key AD features, including cognitive deficits, cholinergic dysfunction, and neuronal damage [6]. Scopolamine also decreases hippocampal levels of brain-derived neurotrophic factor (BDNF) and cAMP response element-binding protein (CREB) [7]. BDNF supports synaptic integrity, enhances memory, and exerts anti-inflammatory effects primarily through CREB activation, which is essential for hippocampal learning and cognitive processes. Due to its central role in cognition, the BDNF/CREB pathway is considered a promising therapeutic target for AD [8].

Lipopolysaccharide (LPS) is a potent inducer of neuroinflammation and microglia activation, derived from Gram-negative bacteria [9]. LPS-activated microglia release several pro-inflammatory cytokines and mediators such as nitric oxide (NO), interleukin-1β (IL-1β), interleukin-6 (IL-6), tumor necrosis factor-α (TNF-α), cyclooxygenase-2 (COX-2), and inducible NO synthase (iNOS), accelerating neuroinflammatory responses and neuronal injury. This further activates microglia, creating a feedback loop of neuroinflammation. LPS also activates toll-like receptor 4 (TLR4), which triggers downstream signaling pathways, including nuclear factor-κB (NF-κB) and mitogen-activated protein kinases (MAPKs) [10,11]. Dysregulated activation of NF-κB and MAPKs (ERK, JNK, p38) is linked to cognitive impairment and neurodegenerative disease. Targeting the NF-κB/MAPK signaling pathways activated by LPS therefore represents a promising therapeutic strategy for neuroinflammation-related conditions.

The raw materials of natural products and their bioactive compounds are widely used as pharmacological agents. Due to their established safety, stability, and efficacy [12], they can offer considerable health benefits with minimal side effects. *R. occidentalis* (black raspberry), a member of the Rosaceae family native to North America, is a widely consumed fruit [13]. The genus includes over 700 species, but only a few, such as *R. occidentalis*, have been commercialized [14]. Black raspberries, raspberries, and Himalayan raspberries are consumed for their sweet, juicy fruits, which are rich in vitamins, minerals, and antioxidants. *R. occidentalis* has been studied for its therapeutic potential and is known for its antioxidant, antimicrobial, and anticancer properties derived from its rich polyphenol and flavonoid content [15]. One study reported that daily administration of 1500–2500 mg of dried black raspberry powder for 8 weeks resulted in a significant reduction in 24 h systolic blood pressure compared with a placebo [16]. A separate 12-week double-blind, placebo-controlled trial found that 4.8 g of black raspberry extract daily was well tolerated and showed no significant adverse effects in men aged 40–75 years [17]. These data suggest that *R. occidentalis* could offer benefits as a therapeutic agent for various diseases. However, its effect on neuroinflammation and cognitive function remains unexplored.

In this study, *R. occidentalis* extracts were prepared from both premature and mature fruits using different solvents. Their anti-inflammatory properties were evaluated in LPS-stimulated BV-2 microglia cells. Of the tested extracts, the ROE exhibited greater anti-inflammatory effects than extracts from mature fruits. Based on these findings, ROE was selected for subsequent experiments. The neuroprotective and anti-inflammatory potential of ROE was investigated in LPS-stimulated BV-2 microglia cells and in a scopolamine-induced amnesic mouse model to assess its effects on neuroinflammation and cognitive function.

## 2. Results

### 2.1. Inhibitory Effect of NO Production and Cell Viability of Mature and Premature R. occidentalis Extracts in LPS-Stimulated BV-2 Microglia Cells

**Figure 1:**
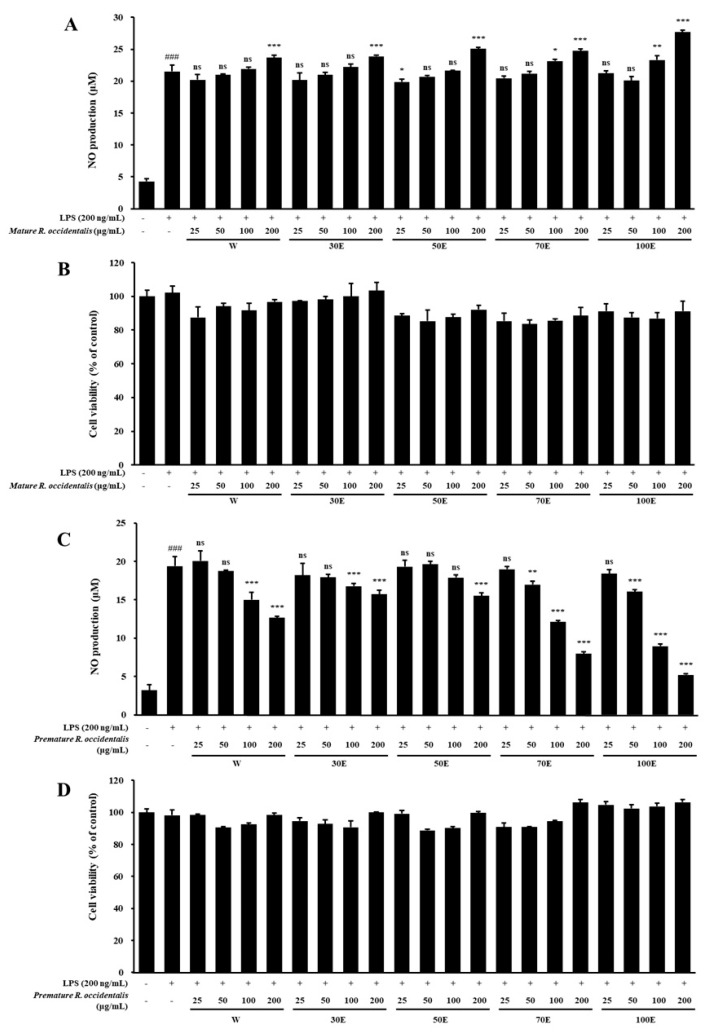
Effects of mature and premature *R. occidentalis* extracts on nitric oxide (NO) production and cell viability in LPS-stimulated BV-2 microglial cells. (**A**,**B**) Mature *R. occidentalis* extracts increased cell viability but showed no significant effect on NO production. (**C**,**D**) Premature *R. occidentalis* extracts increased cell viability and significantly reduced NO production. Data are expressed as mean ± SD (n = 3, independent experiments). ^###^ *p* < 0.001 vs. control; * *p* < 0.05, ** *p* < 0.01, *** *p* < 0.001, and ^ns^ non-significant vs. LPS group. Statistical significance was set at *p* < 0.05. W: water extraction, 30E: 30% ethanol extraction, 50E: 50% ethanol extraction, 70E: 70% ethanol extraction, 100E: 100% ethanol extraction.

Comparison of the neuroprotective and anti-neuroinflammatory effects of extracts from premature and mature *R. occidentalis* fruits showed that the premature extract was more effective. To evaluate the anti-inflammatory effects of mature and premature *R. occidentalis* extracts on NO production and cell viability, BV-2 microglia cells were pretreated with various extract concentrations, followed by stimulation with or without LPS (200 ng/mL) for 24 h. MTT assays demonstrated that neither mature nor premature *R. occidentalis* extracts exhibited cytotoxicity at concentrations up to 200 μg/mL (Figure 1B,D). NO production was measured in LPS-stimulated BV-2 microglia cells treated with the extracts. LPS stimulation significantly increased NO production compared with control (*p* < 0.001). Interestingly, pretreatment with premature *R. occidentalis* extracts resulted in a dose-dependent suppression of LPS-induced NO production; mature extracts did not show significant effects. Of all the tested extracts, the ROE exhibited the most potent inhibitory effect on NO production at all tested doses (*p* < 0.001) (Figure 1C). This suggests that ROE exerts its neuroprotective and anti-inflammatory effects primarily by regulating NO production and suppressing microglial activation. ROE was therefore selected for further in vitro and in vivo experiments.

### 2.2. Total Polyphenol and Flavonoid Content of R. occidentalis Extracts

Table 1 summarizes the total polyphenol and flavonoid contents of different concentrations of mature and premature *R. occidentalis* fruit extracts. The mature fruit extracts (prepared using water and ethanol at varying concentrations) had lower levels of both polyphenols and flavonoids compared with the premature fruit extracts (prepared using water and ethanol). Notably, ROE contained the highest concentrations of total polyphenols (186.063 ± 0.281 mg GAE/g extract) and flavonoids (17.738 ± 0.412 mg CE/g extract) of all samples tested. Based on this, ROE was further investigated for its neuroprotective properties and underlying mechanisms of action.

### 2.3. ROE Inhibits mRNA and Protein Expression of iNOS, COX-2, and Pro-Inflammatory Cytokines in LPS-Stimulated BV-2 Microglia Cells

**Figure 2:**
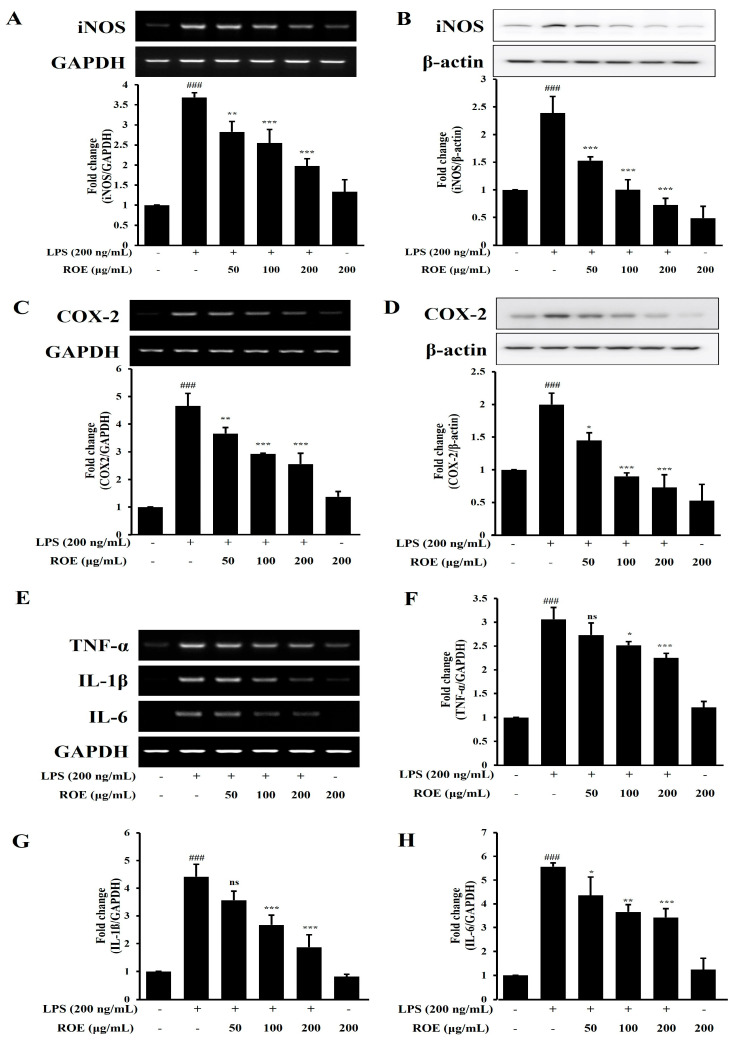
Effects of the ROE on iNOS, COX-2, and pro-inflammatory cytokine (TNF-α, IL-1β, IL-6) expression in LPS-stimulated BV-2 microglial cells. Cells were treated with LPS (200 ng/mL) and ROE (50, 100, 200 μg/mL) for 6 h. (**A**–**H**) LPS markedly increased protein and mRNA expression of iNOS, COX-2, and cytokines; ROE pretreatment significantly suppressed these responses in a dose-dependent manner. β-actin and GAPDH were used as internal controls for Western blotting and RT-PCR, respectively. Data are expressed as mean ± SD (n = 3). ^###^ *p* < 0.001 vs. control; * *p* < 0.05, ** *p* < 0.01, *** *p* < 0.001, and ^ns^ non-significant vs. LPS group. Statistical significance was set at *p* < 0.05.

To investigate the regulatory effects of ROE on inflammatory responses in LPS-stimulated BV-2 microglia cells, BV-2 microglia were pretreated with ROE at the indicated concentrations (50, 100, and 200 μg/mL), with or without LPS (200 ng/mL). COX-2, iNOS, and pro-inflammatory cytokine (TNF-α, IL-1β, IL-6) levels were evaluated using expression and Western blot analyses. As shown in Figure 2A,B, LPS treatment significantly increased iNOS mRNA and protein levels compared with the control group (*p* < 0.001). ROE pretreatment led to a dose-dependent suppression of iNOS expression (*p* < 0.01 and *p* < 0.001), consistent with the prior observations of concentration-dependent NO production inhibition (Figure 1C). Given the pivotal role of COX-2 in inflammation and the production of PGE2, COX-2 expression in BV-2 microglia cells was next examined. LPS significantly upregulated COX-2 mRNA and protein expression (Figure 2C,D; *p* < 0.001); ROE pretreatment (50, 100, and 200 μg/mL) significantly attenuated this induction in a dose-dependent manner (*p* < 0.05, *p* < 0.01, and *p* < 0.001, respectively).

The mRNA expression of TNF-α, IL-1β, and IL-6 was analyzed using RT-PCR. LPS stimulation markedly increased TNF-α, IL-1β, and IL-6 expression levels compared with control (*p* < 0.001; Figure 2E–H). ROE pretreatment significantly reduced their expression in a concentration-dependent manner: TNF-α (*p* < 0.05, *p* < 0.001); IL-1β (*p* < 0.001); and IL-6 (*p* < 0.05, *p* < 0.01, *p* < 0.001).

### 2.4. ROE Inhibited the Phosphorylation of MAPK Signaling in LPS-Stimulated BV-2 Microglia Cells

**Figure 3:**
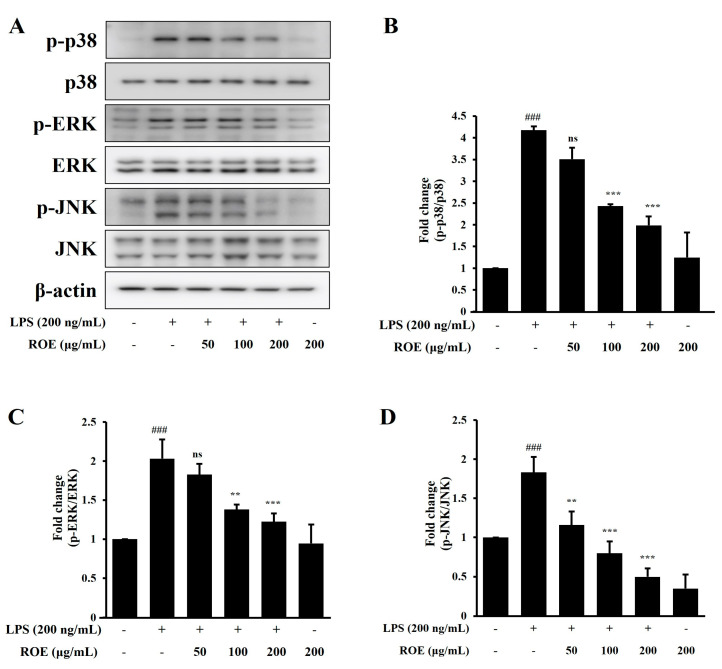
Effects of the ROE on MAPK phosphorylation in LPS-stimulated BV-2 microglia. BV-2 microglia were treated with ROE (50, 100, 200 μg/mL) for 30 min prior to LPS (200 ng/mL) stimulation. LPS significantly increased p38, ERK, and JNK phosphorylation (**A**–**D**). ROE pretreatment reduced the levels of phosphorylated p38, ERK, and JNK in a dose-dependent manner. Total p38, ERK, and JNK were measured alongside their phosphorylated forms using species-specific antibodies; β-actin was the internal control. Data are expressed as mean ± SD (n = 3). ^###^ *p* < 0.001 vs. control; ** *p* < 0.01, *** *p* < 0.001, and ^ns^ non-significant vs. LPS group. Statistical significance was set at *p* < 0.05.

The MAPK signaling pathway plays a key role in regulating pro-inflammatory cytokine production in activated BV-2 microglia cells. To determine whether ROE modulates this pathway, phosphorylation of p38, ERK, and JNK was evaluated following ROE treatment. BV-2 microglia cells were treated with ROE (50, 100, and 200 μg/mL) and LPS (200 ng/mL) for 30 min; the phosphorylation of MAPK-related proteins was assessed by Western blotting. LPS stimulation significantly increased p38, ERK, and JNK phosphorylation compared with untreated controls (*p* < 0.001; Figure 3A–D). ROE pretreatment markedly inhibited LPS-induced phosphorylation of all three proteins in a concentration-dependent manner: p38 (*p* < 0.001); ERK (*p* < 0.01, *p* < 0.001); and JNK (*p* < 0.01, *p* < 0.001). This indicates that the downregulation of iNOS and COX-2 expression by ROE may be mediated by the suppression of MAPK signaling, likely through interference with NF-κB activation.

### 2.5. ROE Inhibited IκB-α Degradation and Nuclear Translocation of NF-κB in LPS-Stimulated BV-2 Microglia Cells

**Figure 4:**
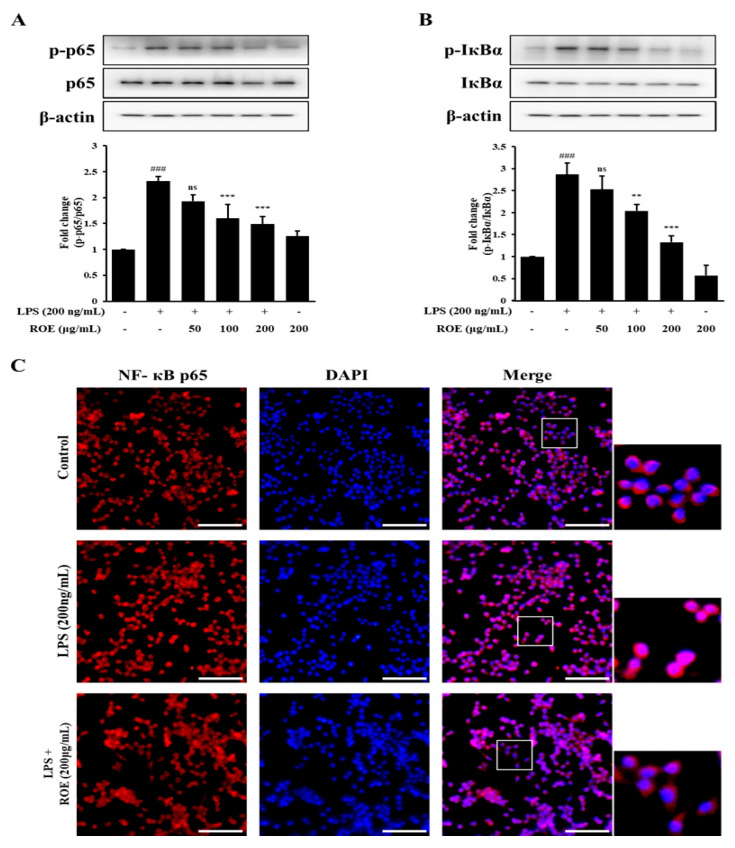
Effects of the ROE on NF-κB signaling in LPS-stimulated BV-2 microglia. BV-2 cells were pretreated with ROE (100, 200, 400 μg/mL) for 30 min before LPS (200 ng/mL) stimulation. (**A**,**B**) Western blot analysis showing phosphorylated and total NF-κB p65 and IκB-α protein levels, with β-actin as the loading control. (**C**) Nuclear translocation of NF-κB p65 assessed by immunocytochemistry following treatment with LPS and/or ROE (200 μg/mL); nuclei stained with DAPI. Scale bar: 100 μm. Data are expressed as mean ± SD (n = 3). ^###^ *p* < 0.001 vs. control; ** *p* < 0.01, *** *p* < 0.001, and ^ns^ non-significant vs. LPS group. Statistical significance was set at *p* < 0.05.

NF-κB is a key transcription factor which regulates the expression of inflammatory mediators such as iNOS and COX-2 in response to LPS or other pro-inflammatory stimuli. To determine whether ROE inhibits NF-κB nuclear translocation, the intracellular localization of NF-κB p65 and IκB-α was examined using Western blotting. Phosphorylation of NF-κB p65 and IκB-α was significantly increased in BV-2 microglia cells following LPS treatment (*p* < 0.001). ROE pretreatment (100, 200, and 400 μg/mL) significantly suppressed LPS-induced phosphorylation of NF-κB p65 (*p* < 0.001) and IκB-α (*p* < 0.01, *p* < 0.001) (Figure 4A,B). The impact of ROE on NF-κB activation was further investigated using immunocytochemistry to evaluate NF-κB p65 nuclear translocation. In unstimulated control cells, NF-κB p65 was predominantly located in the cytoplasm. LPS stimulation induced marked translocation of NF-κB into the nucleus. ROE pretreatment (200 μg/mL) effectively inhibited LPS-induced p65 nuclear translocation in BV-2 microglia cells (Figure 4C).

### 2.6. Effects of ROE on Spatial Learning and Cognitive Impairment in a Scopolamine-Treated Amnesic Mouse Model

**Figure 5:**
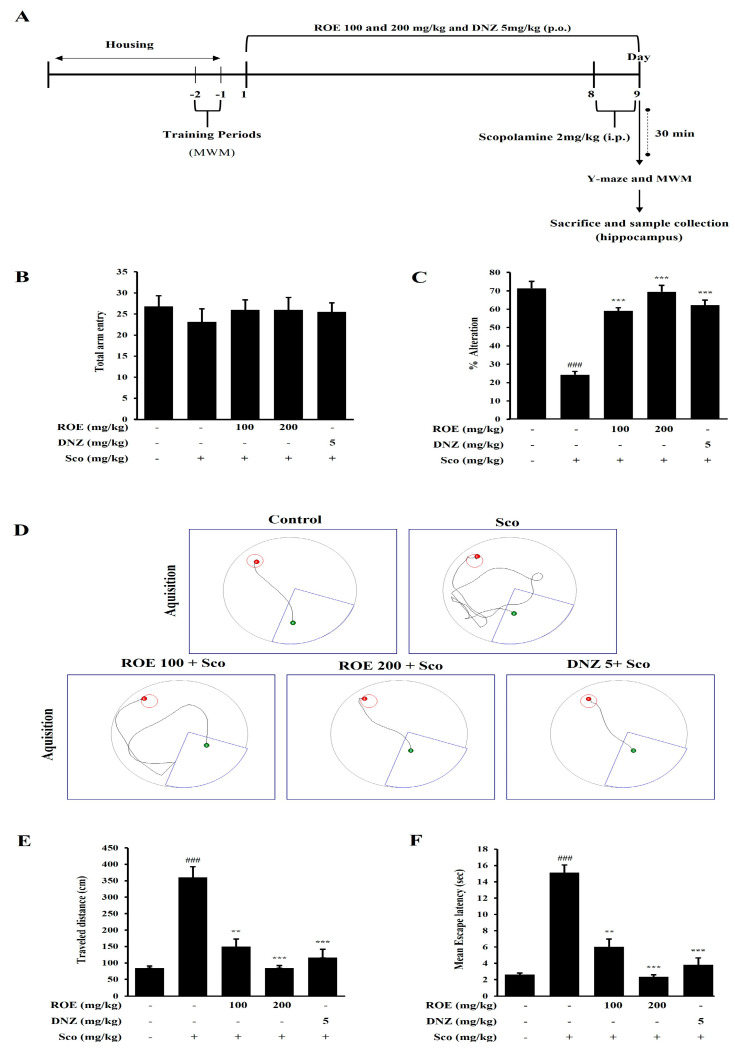
Effects of the ROE on cognitive and behavioral impairments in scopolamine-induced amnesic mice. (**A**) Experimental timeline for behavioral testing. (**B**,**C**) Y-maze test showing the total number of arm entries and percentage of spontaneous alternations. (**D**) Representative swimming paths during the probe trial of the Morris water maze (MWM) test. (**E**,**F**) MWM analysis of total distance traveled and escape latency. Data are expressed as mean ± SD (n = 8). ^###^ *p* < 0.001 vs. control; ** *p* < 0.01, *** *p* < 0.001 vs. scopolamine group. Statistical significance was set at *p* < 0.05.

MCI, which can progress to AD, is commonly associated with deficits in spatial memory learning and cognitive function. To evaluate the effects of ROE on cognitive performance, mice were orally administered ROE (100 or 200 mg/kg) or the positive control drug donepezil (DNZ, 5 mg/kg) for 9 days. Subsequent behavioral assessments were conducted using the Y-maze and Morris water maze (MWM) (Figure 5A). In the Y-maze, scopolamine injection significantly reduced the percentage of spontaneous alternations, indicating impaired working memory (*p* < 0.001). ROE (100 and 200 mg/kg) and DNZ pretreatment significantly restored the alternation percentage in a dose-dependent manner (*p* < 0.001) (Figure 5C). There were no significant between-group differences in the total number of arm entries, suggesting that ROE did not affect locomotor activity (Figure 5B). In the MWM, scopolamine-treated mice exhibited impairments in spatial learning and memory, characterized by significantly increased escape latency and total distance traveled to locate the hidden platform (*p* < 0.001) (Figure 5D–F). ROE (100 and 200 mg/kg) and DNZ pretreatment significantly reduced escape latency and distance traveled (*p* < 0.01 and *p* < 0.001), reflecting dose-dependent cognitive improvement. These findings suggest that ROE ameliorates the cognitive and behavioral deficits associated with scopolamine-induced MCI, underscoring its therapeutic potential for AD-related cognitive dysfunction.

### 2.7. ROE Modulated BDNF/CREB Signaling and Inflammatory Mediator Expression in the Hippocampus of Scopolamine-Treated Amnesic Mice

**Figure 6:**
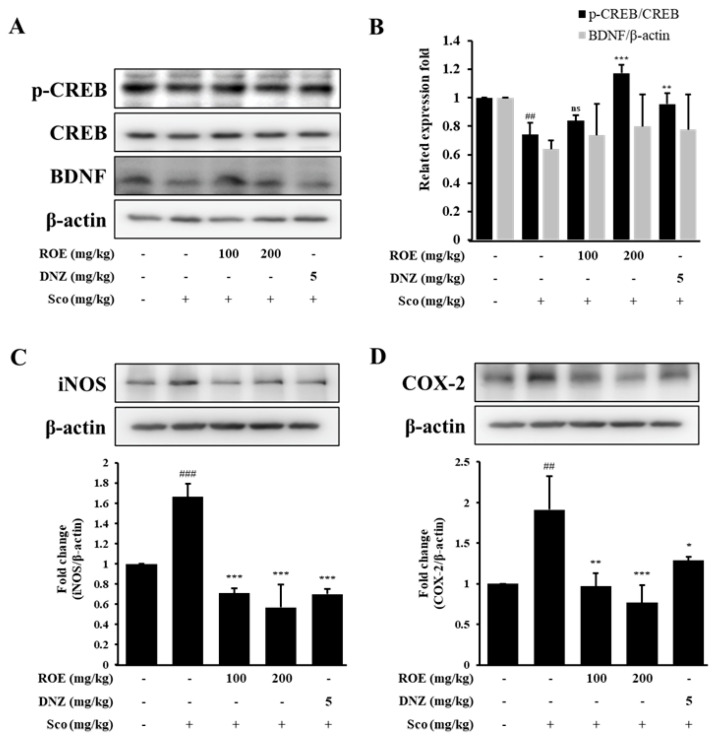
Effects of the ROE on hippocampal protein levels in scopolamine-treated amnesic mice. (**A**–**D**) Western blot analysis of neuroprotective markers (p-CREB, CREB, BDNF) and inflammatory mediators (iNOS, COX-2) in the hippocampus. β-actin was the loading control. Data are expressed as mean ± SD (n = 3). ^###^ *p* < 0.001, ^##^ *p* < 0.01, vs. control; * *p* < 0.05, ** *p* < 0.01, *** *p* < 0.001, and ^ns^ non-significant vs. scopolamine-treated group. Statistical significance was set at *p* < 0.05.

To further investigate the molecular mechanisms underpinning ROE’s protective effects on cognition, the expression of memory-related signaling molecules and inflammatory mediators was investigated in the hippocampus of scopolamine-induced amnesic mice. Western blot analysis revealed that BDNF and phosphorylated CREB (p-CREB) expression was reduced in scopolamine-treated mice compared with controls (*p* < 0.01) (Figure 6A,B). Pretreatment with ROE (100 or 200 mg/kg) or the positive control DNZ (5 mg/kg) restored BDNF and p-CREB expression levels (*p* < 0.05), suggesting that ROE promotes neuroprotective signaling associated with cognitive function. The expression of inflammatory markers was also evaluated to determine whether ROE could attenuate hippocampal neuroinflammation. Scopolamine administration significantly increased the protein expression of iNOS and COX-2 compared to controls (*p* < 0.01 and *p* < 0.001, respectively) (Figure 6C,D). Pretreatment with ROE (100 or 200 mg/kg) and DNZ (5 mg/kg) significantly inhibited iNOS (*p* < 0.001) and COX-2 expression (*p* < 0.01 and *p* < 0.001) compared with the scopolamine-only group. These findings indicate that ROE alleviates scopolamine-induced neuroinflammation and contributes to hippocampal protection by modulating inflammatory and neurotrophic pathways.

### 2.8. Analysis of Miquelianin Using High-Performance Liquid Chromatography

**Figure 7:**
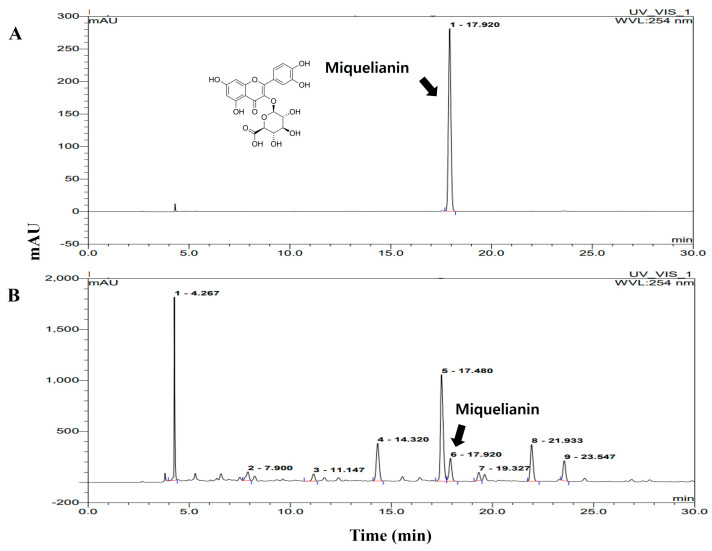
High-performance liquid chromatograms of the MQ standard (**A**) and the ROE (**B**) at 254 nm. MQ retention time was identified at approximately 17.92 min. Comparison with the standard confirmed the presence of MQ in the ROE sample.

The main bioactive components of ROE were investigated using liquid chromatography–mass spectrometry. Five components were identified, including bilobalide, ellagic acid, miquelianin (MQ), 3-O-methylellagic acid, and coumaroyl galloyl citric acid (Supplementary Figure S1). High-performance liquid chromatography (HPLC) confirmed that MQ, a flavonoid known for its antioxidant and anti-inflammatory effects, was a major bioactive component. Given its reported anti-inflammatory potential, subsequent analyses focused on MQ as a key potential contributor to ROE anti-neuroinflammatory activity. HPLC quantification revealed that MQ had a retention time of 17.92 ± 0.04 min compared with the standard chromatogram (Figure 7A vs. Figure 7B). The concentration of MQ in ROE was determined to be 0.37 ± 0.03% (*w/w*).

### 2.9. Anti-Inflammatory Effect of MQ in LPS-Stimulated BV-2 Microglia Cells

**Figure 8:**
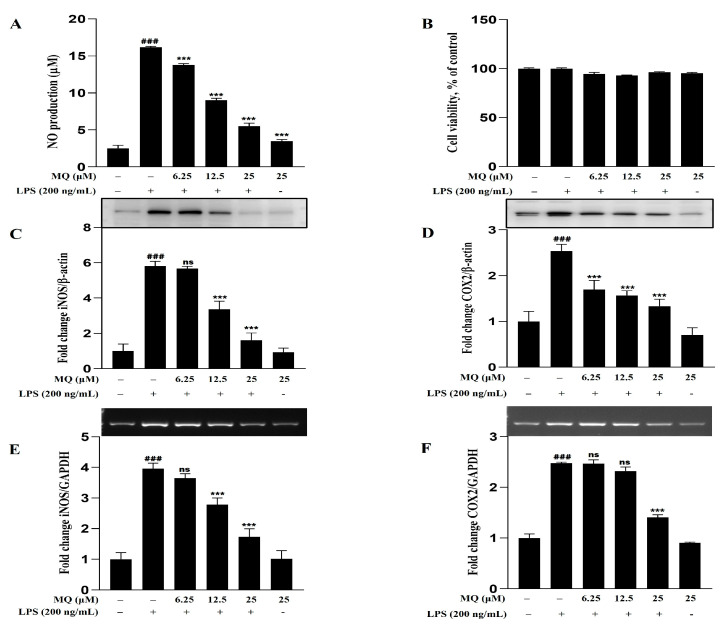
Effects of MQ on inflammation and cell viability in LPS-stimulated BV-2 microglia. BV-2 cells were treated with LPS (200 ng/mL) and MQ (6.25, 12.5, 25 μM) for 18 h. (**A**,**B**) Nitric oxide (NO) production and cell viability were measured. (**C**–**F**) Western blot and RT-PCR analysis of iNOS and COX-2 protein and mRNA levels. β-actin was the loading control. MQ significantly reduced NO production and downregulated iNOS and COX-2 expression. Data are expressed as mean ± SD (n = 3). ^###^ *p* < 0.001 vs. control; *** *p* < 0.001 and ^ns^ non-significant vs. LPS group. Statistical significance was set at *p* < 0.05.

The anti-neuroinflammatory efficacy of MQ was confirmed first by evaluating its ability to inhibit NO production, and then by assessing the protein and mRNA expression levels of iNOS and COX-2 in LPS-stimulated BV-2 microglia cells. As shown in Figure 8A,B, MQ significantly inhibited NO production in a dose-dependent manner (6.25–25 μM) without exhibiting cytotoxicity. Western blot and expression analyses were also conducted. MQ treatment markedly reduced iNOS and COX-2 expression levels in both protein (Figure 8C,D) and mRNA (Figure 8E,F) of LPS-induced BV-2 cells. These findings indicate that MQ exerts potent anti-neuroinflammatory effects and is likely a key active compound in ROE. However, the possibility cannot be excluded that other individual compounds (or synergistic interactions between multiple compounds) contribute to the anti-neuroinflammatory activity of ROE.

## 3. Discussion

Activated microglia-mediated neuroinflammation has been implicated in the early stages of AD and MCI. Natural products and their bioactive compounds have the potential to exert protective effects against various neurodegenerative conditions [18,19]. The current study reports that ROE reduces excessive pro-inflammatory cytokine production by inhibiting microglia-mediated neuroinflammation and blocking the MAPK/NF-κB pathway in BV-2 microglial cells. ROE also modulated neuroprotective and anti-inflammatory responses in C57BL/6J amnesic mice. These findings position ROE as a potential candidate for the prevention or treatment of MCI related to AD.

Microglia are the primary neuroimmune cells of the CNS and play a critical role in maintaining brain homeostasis by regulating immune responses [20]. However, excessive or prolonged activation leads to the continuous release of cytokines and NO, promoting neuronal damage and driving disease progression [21]. Previous research has demonstrated that the exposure of BV-2 microglial cells to LPS for 24 h induces a robust pro-inflammatory response [22]. The inflammatory mediators released by activated microglia can stimulate other nearby microglia, creating a positive feedback loop which fuels chronic neuroinflammation and neurodegeneration [23]. This knowledge has prompted the development of therapies targeting microglial activity in neurodegenerative disorders.

This study investigated the anti-inflammatory potential of ROE in LPS-stimulated BV-2 microglia and found that ROE significantly reduced LPS-induced NO production by inhibiting microglial activation, without affecting cell viability. Indeed, it significantly increased the mRNA and protein levels of pro-inflammatory cytokines (IL-6, IL-1β, and TNF-α) and inflammatory mediators (iNOS and COX-2) in LPS-stimulated BV-2 microglial cells [24]. Consistent with previous studies [25,26], treatment with LPS alone markedly raised the mRNA and protein levels of pro-inflammatory mediators, indicating activated inflammatory responses in BV-2 microglial cells; pretreatment with ROE selectively lowered these LPS-induced mRNA and protein levels. These findings suggest that ROE specifically downregulates the production of pro-inflammatory mediators by suppressing inflammatory responses, while sparing the proliferation of BV-2 microglial cells.

LPS-induced inflammation is primarily driven by activation of the NF-κB and MAPK signaling pathways. NF-κB is highly active in neuroinflammation-affected regions and regulates the expression of multiple pro-inflammatory cytokines [27]. Its activation involves IκK-mediated phosphorylation of p65 at Ser536, followed by nuclear translocation and acetylation. Under normal conditions, NF-κB remains inactive in the cytoplasm, bound to its inhibitor, IκB [28]. In BV-2 microglial cells, LPS stimulation activates both NF-κB and MAPK signaling, with the MAPK pathway playing a particularly important role [29]. MAPK signaling (involving ERK, JNK, and p38), regulates the production of inflammatory mediators and cytokines which drive neuroinflammation [30]. LPS activates p38 via TLR4, leading to increased cytokine release; JNK responds to cytokines and growth factors, contributing to tau pathology [31]. The MEK/ERK pathway also enhances cytokine expression [32]. Studies have demonstrated that treating BV-2 cells with LPS increases the protein expression levels of p-p38 MAPK and NF-κB genes approximately 1.5–80-fold compared with controls [33,34]. Consistent with previous findings in BV-2 cells [8,35], the current study observed that treatment with LPS alone significantly increased p-p38, p-Erk, p-JNK, p-IκB-α, and p-p65 protein levels and promoted NF-κB p65 translocation to the nucleus, suggesting that LPS activates MAPK and NF-κB signaling cascades. However, ROE suppressed the activation of key proteins in the NF-κB and MAPK signaling pathways in a dose-dependent manner, and altered the robust nuclear translocation of NF-κB subunit p65 in response to LPS stimulation.

Based on the in vitro findings, further investigations were conducted into the therapeutic potential of ROE for brain dysfunction and its ability to mimic MCI following scopolamine injection (2 mg/kg, i.p.) in a mouse model of memory impairment. Scopolamine impairs cholinergic signaling by antagonizing muscarinic acetylcholine receptors in the CNS, inducing memory and cognitive dysfunction [36]. One study found that administering scopolamine resulted in impaired hippocampus-dependent cognitive performance and memory decline in an AD mouse model [37]. The hippocampus, which plays a central role in memory stability and consolidation, is one of the first regions affected during AD progression [38]. Evidence from previous studies indicates that hippocampal myelin dysfunction is closely associated with cognitive decline in AD [39]. Therefore, antiamnesic potential was assessed in terms of both long- and short-term memory using the Y-maze and MWM tests.

The Y-maze test evaluated hippocampus-dependent working memory and exploration behavior. Scopolamine-treated mice exhibited a markedly reduced percentage of spontaneous alternations compared with the control group. Notably, ROE administration (100 or 200 mg/kg) significantly improved spontaneous alternation behavior in a dose-dependent manner, indicating enhanced cognitive performance. Total arm entries were consistent across all groups, confirming that the results were not influenced by locomotor activity. Regarding spatial memory, as evaluated by the hippocampus-dependent MWM, scopolamine caused significant memory deficits reflected in increased escape latency and total distance traveled. ROE administration at all tested doses markedly improved performance, indicating an effective normalization of scopolamine-induced cognitive dysfunction. This aligns with previous reports [40], wherein the administration of ashwagandha root extract (300 mg twice daily for 8 weeks) resulted in significant improvements in memory and cognitive function. Collectively, these findings demonstrate the potential of ROE as a promising neuroprotective agent for memory impairment related to cholinergic dysfunction and MCI.

The p-CREB/BDNF signaling axis plays a crucial role in neurodevelopment, synaptic plasticity, neuronal survival, and the formation and storage of long-term memory [41]. BDNF, a key neurotrophic factor in the hippocampus, is transcriptionally regulated by CREB, a transcription factor essential for learning and memory-related gene expression [42]. Previous studies have shown that scopolamine administration decreases p-CREB and BDNF expression in the hippocampus, resulting in impaired memory and cognitive function [43]. Consistent with these findings, Western blot analysis conducted to evaluate hippocampal CREB and BDNF expression revealed that scopolamine reduced p-CREB and BDNF expression levels. Notably, ROE (100 or 200 mg/kg) reversed the scopolamine-induced downregulation of p-CREB and BDNF expression in the hippocampus, suggesting that ROE may enhance cognitive function by activating the CREB/BDNF signaling pathway. Previously, a randomized, double-blind, placebo-controlled study reported that a polyphenolic-rich herbal extract (100 or 300 mg/kg) taken for 4 weeks increased serum BDNF levels and improved neurocognitive function when compared with a placebo [44]. While ROE administration appeared to restore hippocampal p-CREB and BDNF expression levels in scopolamine-treated mice, further studies with increased biological replicates are necessary to validate this observation.

Neuroinflammation is a major contributor to cognitive decline. Scopolamine has been shown to trigger the excessive release of pro-inflammatory mediators [45] and promote neuroinflammation by increasing oxidative stress and elevating inflammatory factors in the hippocampus and cortex [46]. Among these factors, iNOS and COX-2 are key neurotoxic mediators regulated by NF-κB signaling and are strongly linked to brain disorders [47]. This study investigated the anti-neuroinflammatory effects of ROE in vivo by analyzing hippocampal iNOS and COX-2 expression; ROE treatment significantly reduced scopolamine-induced upregulation of these proteins. Altogether, the findings indicate that ROE restores hippocampal CREB/BDNF signaling and mitigates neuroinflammation, positioning it as a promising candidate for managing cognitive impairment related to neuroinflammation and neurodegenerative conditions.

Further analysis identified MQ as a major constituent of ROE [48,49]. MQ also exhibited anti-inflammatory effects in LPS-stimulated BV-2 microglial cells, reducing NO production and downregulating iNOS and COX-2 mRNA and protein expression levels in a dose-dependent manner. This suggests that MQ may be a key mediator of ROE’s anti-neuroinflammatory activity and highlights its potential as a bioactive dietary polyphenol for preventing or managing neurodegeneration associated with chronic inflammation.

## 4. Materials and Methods

### 4.1. Reagents

*R. occidentalis* was acquired from Gochang (Jeonbuk, Korea). Dimethyl sulfoxide (DMSO), LPS, chloroform, isopropanol, 2-mercaptoethanol, scopolamine hydrobromide, MTT reagent, and DNZ were obtained from Sigma-Aldrich (St. Louis, MO, USA). Dulbecco’s modified Eagle’s medium (DMEM) and phosphate-buffered saline (PBS) were acquired from Welgene (Gyeongsang-do, South Korea). TRIzol was obtained from Invitrogen (Carlsbad, CA, USA). PVDF membranes (Millipore, Bedford, MA, USA) and protease and phosphatase inhibitors (Boston, MA, USA). All other analytical-grade chemicals and reagents were purchased from local suppliers.

### 4.2. Preparation of R. occidentalis Extracts

The *R. occidentalis* (black raspberry) fruit used in this experiment was grown in June in Gochang. The fruits, harvested 28 days after flowering, were categorized as premature, or mature after 35 days, and stored in a freezer at −20 °C. Premature fruits were dried with hot air; mature fruits were used raw. After washing the fruits, 1 L ethanol per 100 g was added at concentrations of 25%, 50%, 75%, and 100%, then extracted using ultrasonic waves three times for 1 h. The water extract was decocted at 100 °C for 2 h with the same amount of distilled water. Each extract was passed through Whatman No. 41 filter paper, concentrated under reduced pressure with a rotary vacuum evaporator (N-1000, EYELA Co., Tokyo, Japan), and subsequently freeze-dried to obtain a powdered product.

### 4.3. Total Phenolic and Flavonoid Content

The total phenolic content of *R. occidentalis* leaves was determined using the Folin–Ciocalteu phenol reagent method. The total flavonoid content was assessed by the aluminum chloride colorimetric assay. Gallic acid and catechin were the respective standards. Results were expressed as milligrams of gallic acid/catechin per gram of extract.

### 4.4. HPLC Analysis of MQ from ROE

Analysis was performed on an Acquity UPLC system (Waters, MA, USA) with a binary pump, autosampler, and PDA-UV detector. Separation used a BEH C18 column (2.1 × 50 mm, 1.7 μm) with 0.08% TFA (A) and acetonitrile (B) under the following gradient: 0–4.59 min, 90% A; 5–5.59 min, 80% A; 6–6.59 min, 79% A; 7–7.59 min, 78% A; 8–8.59 min, 77% A; 9–9.59 min, 76% A; 10–12.29 min, 75% A; 12.30–12.59 min, 10% A; 13–16 min, 90% A. The flow rate was 0.11 mL/min, the injection volume was 2 μL, the column temperature was 25 ± 1 °C, and detection was at 356 nm. Mobile phases and samples were filtered through a 0.22-μm membrane prior to use. Data were acquired with Empower 3 software.

#### 4.4.1. Cell Culture and Treatment

BV-2 mouse microglia cells were cultured in DMEM with 5% FBS and kept at 37 °C in a controlled atmosphere with 5% CO_2_. The cells were grown until they reached approximately 70–80% confluence, then were cultured in 24-well plates for further experiments.

#### 4.4.2. Cell Viability and NO Assay

BV-2 microglia cells were seeded at a density of 10 × 10^4^ cells/well in 24-well plates for 24 h. Cells were then pretreated with different concentrations of *R. occidentalis* (premature and mature) extracts, with or without LPS (200 ng/mL) for 24 h. For the NO assay, 100 μL of supernatant was combined with an equal volume of Griess reagent in 96-well plates. This mixture was incubated at room temperature for 30 min to facilitate the reaction. The absorbance of the reaction mixture was obtained at 540 nm using a microplate reader. For cell viability, 0.5 mg/mL of MTT solution was added, with incubation for 1 h in the dark. The supernatant was then discarded, and 400 μL of DMSO solution was added for 2 h. The absorbance was measured at 552 nm using a microplate reader [30].

#### 4.4.3. Total RNA Isolation and RT-PCR

TRIzol reagent was used according to the manufacturer’s protocol to isolate total RNA from BV-2 microglia cells. Reverse transcription was subsequently carried out using the appropriate reagent (Promega, Madison, WI, USA) following the manufacturer’s guidelines. The specific primers used for RT-PCR were obtained from Geneer (Daejeon, South Korea) and are presented in Supplementary Table S1. Electrophoresis was performed using a 1.2% agarose gel (Davinch-K); each experiment was performed in triplicate [50].

#### 4.4.4. Immunofluorescence

Nuclear translocation of the p-NF-κB subunit in BV-2 microglial cells was investigated using immunofluorescence. Cells were seeded into 24-well plates, allowed to adhere overnight, then treated with ROE (200 μg/mL) and LPS (200 ng/mL). Following treatment, cells were fixed, permeabilized with 0.1% Triton X-100 for 10 min at room temperature, and rinsed twice with PBS (5 min each). Cells were incubated overnight at 4 °C with an anti-p-NF-κB primary antibody, washed again with PBS, and exposed to a chicken anti-mouse secondary antibody (CAR-594; A21201, Invitrogen) for 1 h at room temperature. After two additional PBS washes (10 min each), cells were counterstained with DAPI (2 μg/mL). Images were obtained using a fluorescence microscope (NIS-Elements, Melville, NY, USA).

### 4.5. Animals

#### 4.5.1. Animal Handling and Treatment

Animal experiments were approved by the Institutional Animal Care and Use Committee of Konkuk University (approval no. KU23155). Male C57BL/6N mice (8 weeks old, 25–27 g; n = 9 per group) were obtained from Daehan Bio-Link (Korea). Mice were housed under controlled temperatures and a 12 h light/dark cycle with unrestricted access to food and water. After a 7-day acclimatization period, 45 animals were randomly assigned to five groups: (i) control (0.9% saline); (ii) scopolamine (2 mg/kg); (iii) ROE (100 mg/kg) + scopolamine; (iv) ROE (200 mg/kg) + scopolamine; and (v) DNZ (5 mg/kg) + scopolamine. ROE was administered orally for 9 days. Scopolamine injection (i.p.) was administered on Days 8 and 9. ROE dosages were selected based on published data [51,52]. Behavioral performance was assessed 30 min after the final scopolamine injection using the Y-maze and MWM tests. Mice were anesthetized following behavioral evaluation. Hippocampal tissues were collected and stored at −80 °C for Western blot analysis. The treatment schedule is summarized in Figure 5A.

#### 4.5.2. The Y-Maze Test

The Y-maze test was used to evaluate spatial and short-term working memory. The apparatus consisted of three identical arms arranged at 120° angles, each 15 cm long, 3.5 cm wide, and enclosed by 3 cm walls. Mice were placed at the center and allowed 1 min to explore before testing. Spontaneous alternations and latency to enter different arms were recorded over 5 min. The alternation percentage was calculated as [number of alternations/(total arm entries − 2)] × 100 [8].

#### 4.5.3. The MWM Test

The MWM was used to evaluate spatial learning and memory. The test was conducted in a circular pool (122 cm diameter, 35 cm height, 20 cm water depth) with water maintained at 22 ± 2 °C. The water was rendered opaque with a nontoxic white paint solution to hide the submerged platform. Mice were allowed to freely navigate the pool to locate the hidden platform. Prior to treatment, animals underwent training for 2 days with two consecutive trials per session per day; after reaching the platform, they were allowed to rest for 7 s. At the end of the experiment, spatial learning and memory performance were analyzed using SMART 3.0 software (Harvard Apparatus, Holliston, MA, USA) [8].

#### 4.5.4. Western Blot Analysis

Hippocampal tissue and BV-2 microglia were lysed in RIPA buffer containing protease and phosphatase inhibitors. The homogenates were centrifuged at 13,000 rpm for 15 min at 4 °C to obtain the supernatant. Protein levels were measured using the Bio-Rad DC assay kit. Equal amounts of protein were separated on 8–15% SDS-PAGE gels, transferred to PVDF membranes, and blocked for 1 h with either 5% skim milk or 3% BSA. The membranes were incubated overnight at 4 °C with primary antibodies and subsequently with anti-mouse or anti-rabbit secondary antibodies for 1 h at room temperature. Protein bands were visualized using the Immobilon Forte HRP substrate, detected by chemiluminescence, and quantified with NIH ImageJ software (Version 1.53).

### 4.6. Statistical Analysis

Data were analyzed using GraphPad Prism 8.0.1 (Dotmatics, La Jolla, CA, USA) and expressed as mean ± SD. Comparisons were performed using one-way ANOVA with Tukey’s multiple comparison test. All in vitro and in vivo experiments were independently repeated at least three times. A *p*-value < 0.05 was considered statistically significant.

## 5. Conclusions

This study demonstrates that ROE exerts anti-neuroinflammatory effects in LPS-stimulated BV-2 microglial cells by suppressing the MAPK and NF-κB signaling pathways. ROE reduced NO production and inhibited the expression of pro-inflammatory mediators, including iNOS and COX-2, at both the mRNA and protein levels. In vivo, ROE alleviated scopolamine-induced cognitive deficits in mice, improving spatial memory and learning. ROE treatment also restored the expression of memory-related proteins such as p-CREB and BDNF, while decreasing inflammation-associated markers, including iNOS and COX-2. Moreover, MQ—a major bioactive compound in ROE—reduced NO production and downregulated iNOS and COX-2 in LPS-activated BV-2 microglial cells, indicating a significant contribution to ROE’s pharmacological effects. Collectively, these in vivo and in vitro findings demonstrate that ROE and one of its active constituents, MQ, hold therapeutic potential in the management of neuroinflammatory conditions associated with cognitive impairment. Further studies are needed to confirm these effects in clinical settings and clarify the potential contributions of other bioactive compounds in ROE.

## Figures and Tables

**Table 1:** Total polyphenol and flavonoid contents of *R. occidentalis* extracts.

Samples	Total Polyphenol Content ^1^	Total Flavonoid Content ^2^	Samples	Total Polyphenol Content ^1^	Total Flavonoid Content ^2^
Mat; W	61.116 ± 2.714	13.690 ± 0.412	Pre; W	115.243 ± 0.763	16.310 ± 0.412
Mat; 30E	48.683 ± 0.346	9.405 ± 0.412	Pre; 30E	91.593 ± 0.229	12.976 ± 0.412
Mat; 50E	49.556 ± 0.079	8.452 ± 0.412	Pre; 50E	103.497 ± 0.874	17.024 ± 0.412
Mat; 70E	48.656 ± 0.255	9.167 ± 0.412	Pre; 70E	175.548 ± 3.087	13.929 ± 0.000
Mat; 100E	51.884 ± 0.200	9.881 ± 0.412	Pre; 100E	186.063 ± 0.281	17.738 ± 0.412

^1^ Total polyphenol content (mg GAE/g extract), ^2^ Total flavonoid content (mg CE/g extract). Mat: mature fruits, Pre: premature fruits, W: water extraction, 30E: 30% ethanol extraction, 50E: 50% ethanol extraction, 70E: 70% ethanol extraction, 100E: 100% ethanol extraction.

## Data Availability

Data is contained in the paper.

## Supplementary Materials

The following supporting information can be downloaded at: https://www.mdpi.com/article/10.3390/ph18101557/s1, Supplement Material S1: Figure S1: LC-MS chromatograms (A) and chemical composition of ROE (B). Supplement Material S2: Table S1: The specific primer sequences used in this current study; Supplement Material S3—Raw data of in vitro and in vivo studies; Supplement Material S4—Escape latency (MWM); Supplement Material S5—Traveled distance (MWM).
