# Supplementary material for: Rubus occidentalis Ethanol Extract Attenuates Neuroinflammation and Cognitive Impairment in Lipopolysaccharide-Stimulated Microglia and Scopolamine-Induced Amnesic Mice
Source: Pharmaceuticals (Basel). 2025 Oct 16;18(10):1557. doi: 10.3390/ph18101557 (PMC12566997; doi:10.3390/ph18101557)
Supplement: Supplementary file 1 [file pharmaceuticals-18-01557-s001.zip › Supplement Material S1; Figure S1.pdf]

LC-MS analysis

A

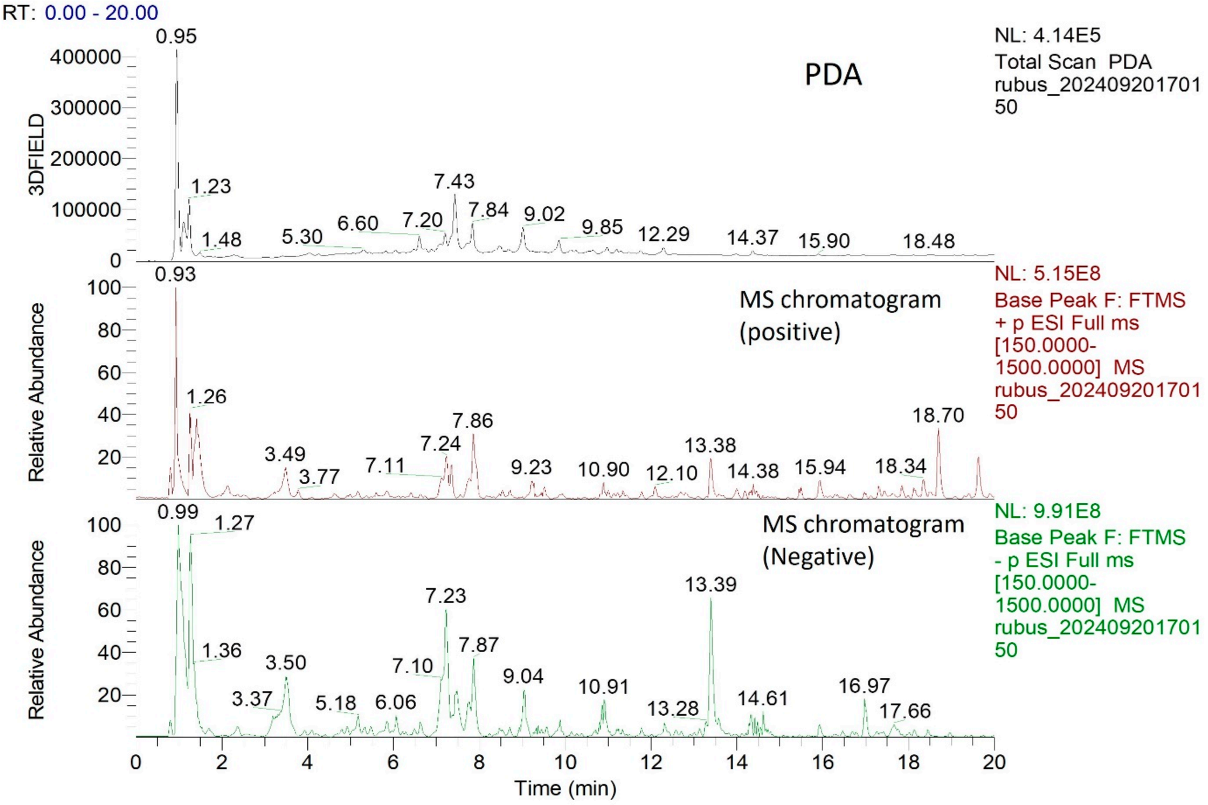

B

RT(min)	m/z ([M+H]$^+$)	Formula ([M+H]$^+$)	Δ ppm	Compound
7.22	327.1071	C15 H19 O8	-1.082	Bilobalide
7.47	300.9991 ([M-H]$^-$)	C14 H5 O8 ([M-H]$^-$)	4.041	Ellagic acid
7.86	479.0814	C21 H19 O13	-1.371	Miquelianin
9.04	315.0147 ([M-H]$^-$)	C15 H7 O8 ([M-H]$^-$)	3.703	3-O-methylellagic acid
9.94	491.0817	C22 H19 O13	-0.707	coumaroyl galloyl citric acid

Figure S1. LC-MS chromatograms (A) and chemical composition of ROE (B)
