# Supplementary material for: Rubus occidentalis Ethanol Extract Attenuates Neuroinflammation and Cognitive Impairment in Lipopolysaccharide-Stimulated Microglia and Scopolamine-Induced Amnesic Mice
Source: Pharmaceuticals (Basel). 2025 Oct 16;18(10):1557. doi: 10.3390/ph18101557 (PMC12566997; doi:10.3390/ph18101557)
Supplement: Supplementary file 1 [file pharmaceuticals-18-01557-s001.zip › Supplement Material S2; Table S1.pdf]

Table S1. Detailed forward and backward primer sequences used in this current study.

Gene		Sequence (5'→3')	Accession No.	bp
iNOS	Forward	GAGGTACTCAGCGTGCTCCA	NM_010927	444
	Reverse	AGGGAGGAAAGGGAGAGAGG		
COX-2	Forward	TGAGTGGTAGCCAGCAAAGC	NM_011198	319
	Reverse	CTGCAGTCCAGGTTCAATGG		
IL-1β	Forward	CAAGGAGAACCAAGCAACGA	NM_008361	428
	Reverse	TTGGCCGAGGACTAAGGAGT		
IL-6	Forward	GGAGGCTTAATTACACATGTT	NM_031168	435
	Reverse	TGATTTCAAGATGAATTGGAT		
TNF-α	Forward	AGGGAGAGTGGTCAGGTTGC	NM_013693	392
	Reverse	CAGCCTGGTCACCAAATCAG		
