# Supplementary material for: Rubus occidentalis Ethanol Extract Attenuates Neuroinflammation and Cognitive Impairment in Lipopolysaccharide-Stimulated Microglia and Scopolamine-Induced Amnesic Mice
Source: Pharmaceuticals (Basel). 2025 Oct 16;18(10):1557. doi: 10.3390/ph18101557 (PMC12566997; doi:10.3390/ph18101557)
Supplement: Supplementary file 1 [file pharmaceuticals-18-01557-s001.zip › Supplement Material S3; Raw data of in vitro and in vivo studies.pptx]

## Slide 1
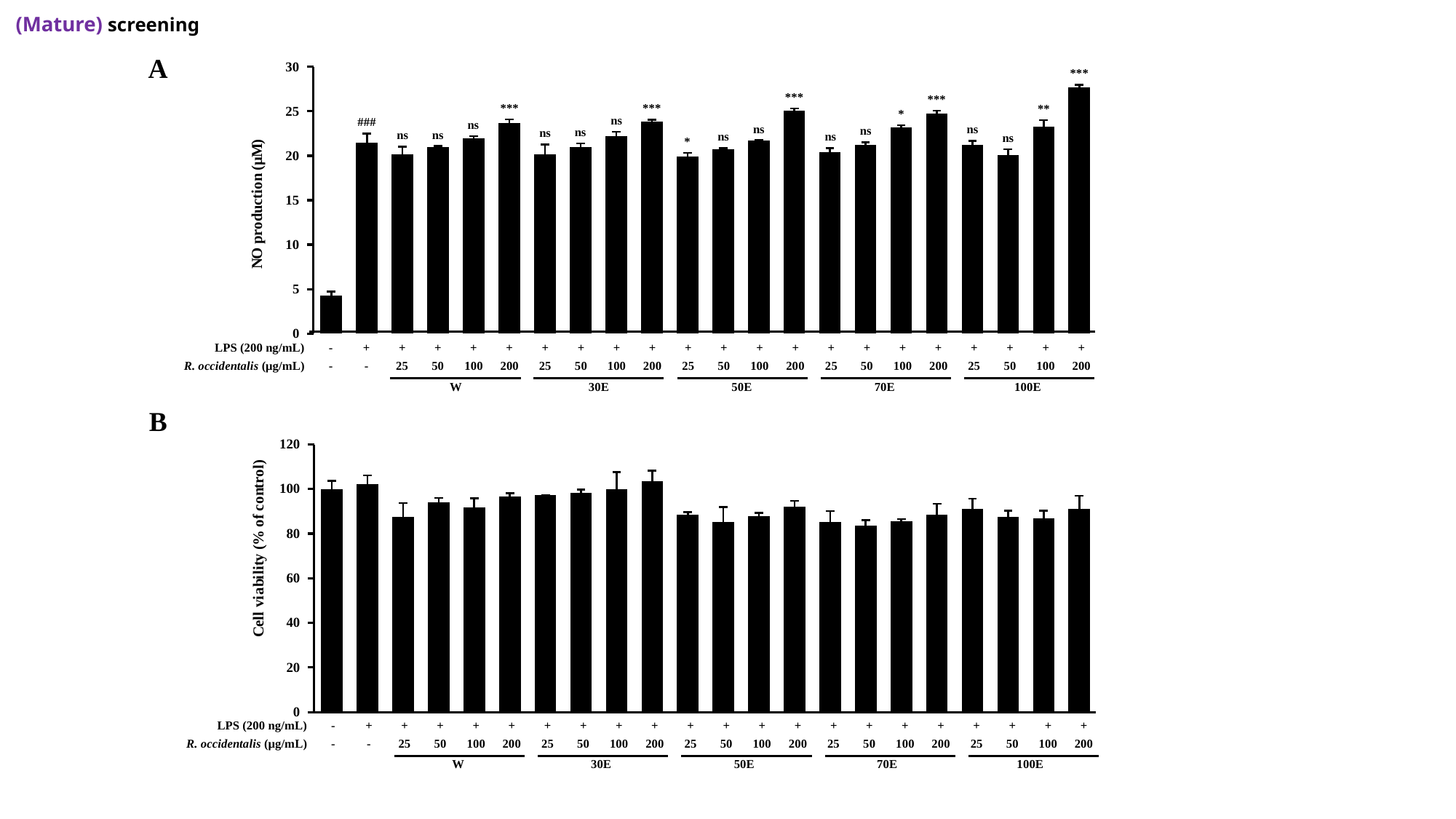

# (Mature) screening
A
### Chart
| Category | |
|---|---|
| NT | 4.286231884057972 |
| LPS | 21.496376811594203 |
| 25 | 20.192028985507246 |
| 50 | 20.98913043478261 |
| 100 | 21.931159420289855 |
| 200 | 23.670289855072465 |
| 25 | 20.155797101449277 |
| 50 | 20.98913043478261 |
| 100 | 22.221014492753625 |
| 200 | 23.887681159420293 |
| 25 | 19.902173913043477 |
| 50 | 20.69927536231884 |
| 100 | 21.67753623188406 |
| 200 | 25.083333333333332 |
| 25 | 20.445652173913043 |
| 50 | 21.206521739130434 |
| 100 | 23.16304347826087 |
| 200 | 24.757246376811594 |
| 25 | 21.242753623188406 |
| 50 | 20.083333333333332 |
| 100 | 23.30797101449275 |
| 200 | 27.69202898550725 |***
***
***
***
***
**
*
ns
###
ns
ns
ns
ns
ns
ns
ns
ns
ns
ns
ns
*
| LPS (200 ng/mL) | - | + | + | + | + | + | + | + | + | + | + | + | + | + | + | + | + | + | + | + | + | + |
| --- | --- | --- | --- | --- | --- | --- | --- | --- | --- | --- | --- | --- | --- | --- | --- | --- | --- | --- | --- | --- | --- | --- |
| R. occidentalis (μg/mL) | - | - | 25 | 50 | 100 | 200 | 25 | 50 | 100 | 200 | 25 | 50 | 100 | 200 | 25 | 50 | 100 | 200 | 25 | 50 | 100 | 200 |
| | | | W | | | | 30E | | | | 50E | | | | 70E | | | | 100E | | | |
### Chart
| Category | |
|---|---|
| NT | 100.0 |
| LPS | 102.21139430284856 |
| 25 | 87.51874062968514 |
| 50 | 94.07796101949025 |
| 100 | 91.6791604197901 |
| 200 | 96.70164917541229 |
| 25 | 97.22638680659671 |
| 50 | 98.20089955022488 |
| 100 | 100.07496251874062 |
| 200 | 103.44827586206895 |
| 25 | 88.49325337331334 |
| 50 | 85.34482758620689 |
| 100 | 87.70614692653673 |
| 200 | 92.05397301349325 |
| 25 | 85.26986506746627 |
| 50 | 83.58320839580209 |
| 100 | 85.60719640179909 |
| 200 | 88.6056971514243 |
| 25 | 91.22938530734632 |
| 50 | 87.44377811094452 |
| 100 | 86.84407796101948 |
| 200 | 91.26686656671663 |B
| LPS (200 ng/mL) | - | + | + | + | + | + | + | + | + | + | + | + | + | + | + | + | + | + | + | + | + | + |
| --- | --- | --- | --- | --- | --- | --- | --- | --- | --- | --- | --- | --- | --- | --- | --- | --- | --- | --- | --- | --- | --- | --- |
| R. occidentalis (μg/mL) | - | - | 25 | 50 | 100 | 200 | 25 | 50 | 100 | 200 | 25 | 50 | 100 | 200 | 25 | 50 | 100 | 200 | 25 | 50 | 100 | 200 |
| | | | W | | | | 30E | | | | 50E | | | | 70E | | | | 100E | | | |

## Slide 2
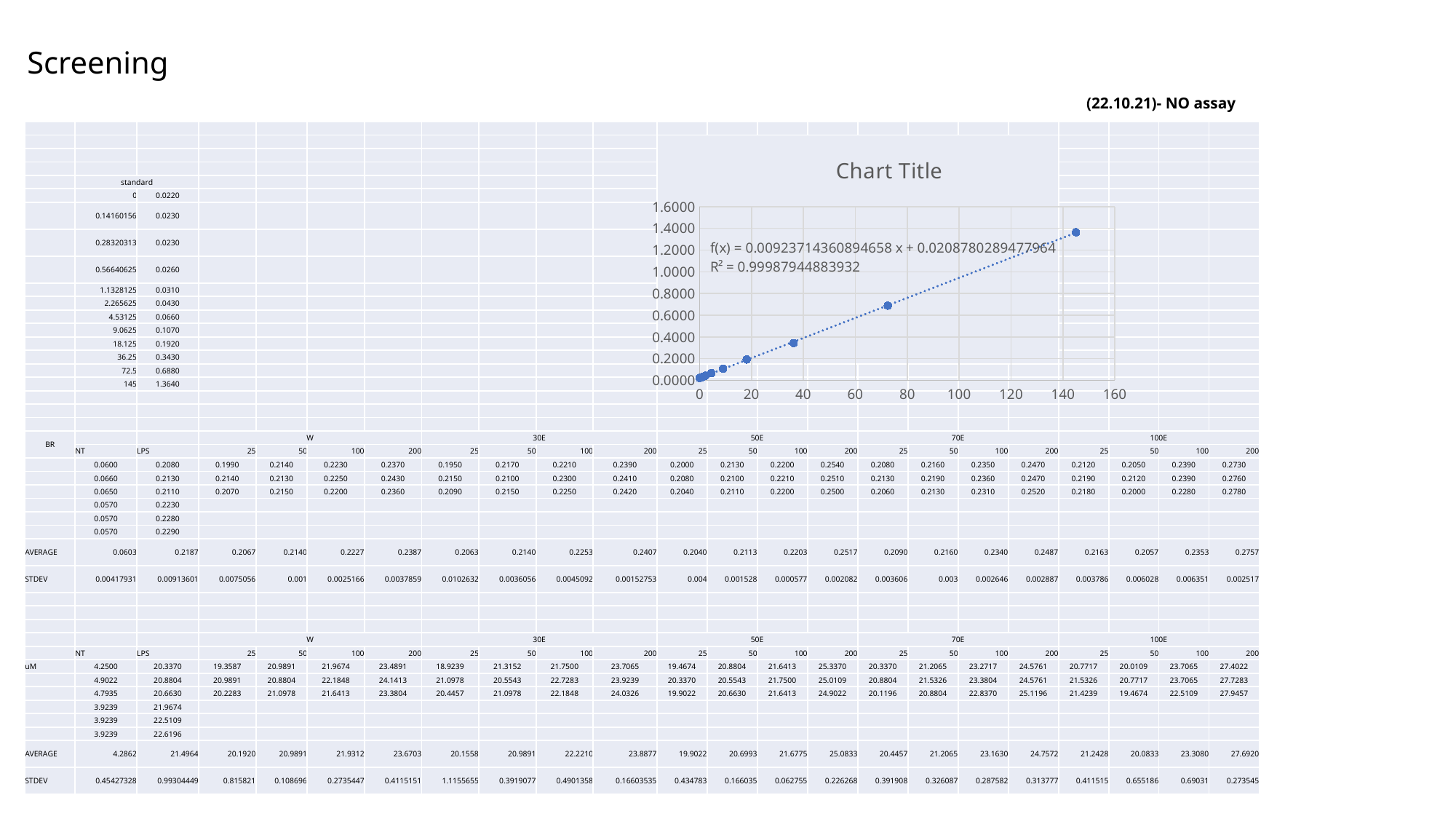

# Screening
(22.10.21)- NO assay
| | | | | | | | | | | | | | | | | | | | | | | |
| --- | --- | --- | --- | --- | --- | --- | --- | --- | --- | --- | --- | --- | --- | --- | --- | --- | --- | --- | --- | --- | --- | --- |
| | | | | | | | | | | | | | | | | | | | | | | |
| | | | | | | | | | | | | | | | | | | | | | | |
| | | | | | | | | | | | | | | | | | | | | | | |
| | standard | | | | | | | | | | | | | | | | | | | | | |
| | 0 | 0.0220 | | | | | | | | | | | | | | | | | | | | |
| | 0.14160156 | 0.0230 | | | | | | | | | | | | | | | | | | | | |
| | 0.28320313 | 0.0230 | | | | | | | | | | | | | | | | | | | | |
| | 0.56640625 | 0.0260 | | | | | | | | | | | | | | | | | | | | |
| | 1.1328125 | 0.0310 | | | | | | | | | | | | | | | | | | | | |
| | 2.265625 | 0.0430 | | | | | | | | | | | | | | | | | | | | |
| | 4.53125 | 0.0660 | | | | | | | | | | | | | | | | | | | | |
| | 9.0625 | 0.1070 | | | | | | | | | | | | | | | | | | | | |
| | 18.125 | 0.1920 | | | | | | | | | | | | | | | | | | | | |
| | 36.25 | 0.3430 | | | | | | | | | | | | | | | | | | | | |
| | 72.5 | 0.6880 | | | | | | | | | | | | | | | | | | | | |
| | 145 | 1.3640 | | | | | | | | | | | | | | | | | | | | |
| | | | | | | | | | | | | | | | | | | | | | | |
| | | | | | | | | | | | | | | | | | | | | | | |
| | | | | | | | | | | | | | | | | | | | | | | |
| BR | | | W | | | | 30E | | | | 50E | | | | 70E | | | | 100E | | | |
| | NT | LPS | 25 | 50 | 100 | 200 | 25 | 50 | 100 | 200 | 25 | 50 | 100 | 200 | 25 | 50 | 100 | 200 | 25 | 50 | 100 | 200 |
| | 0.0600 | 0.2080 | 0.1990 | 0.2140 | 0.2230 | 0.2370 | 0.1950 | 0.2170 | 0.2210 | 0.2390 | 0.2000 | 0.2130 | 0.2200 | 0.2540 | 0.2080 | 0.2160 | 0.2350 | 0.2470 | 0.2120 | 0.2050 | 0.2390 | 0.2730 |
| | 0.0660 | 0.2130 | 0.2140 | 0.2130 | 0.2250 | 0.2430 | 0.2150 | 0.2100 | 0.2300 | 0.2410 | 0.2080 | 0.2100 | 0.2210 | 0.2510 | 0.2130 | 0.2190 | 0.2360 | 0.2470 | 0.2190 | 0.2120 | 0.2390 | 0.2760 |
| | 0.0650 | 0.2110 | 0.2070 | 0.2150 | 0.2200 | 0.2360 | 0.2090 | 0.2150 | 0.2250 | 0.2420 | 0.2040 | 0.2110 | 0.2200 | 0.2500 | 0.2060 | 0.2130 | 0.2310 | 0.2520 | 0.2180 | 0.2000 | 0.2280 | 0.2780 |
| | 0.0570 | 0.2230 | | | | | | | | | | | | | | | | | | | | |
| | 0.0570 | 0.2280 | | | | | | | | | | | | | | | | | | | | |
| | 0.0570 | 0.2290 | | | | | | | | | | | | | | | | | | | | |
| AVERAGE | 0.0603 | 0.2187 | 0.2067 | 0.2140 | 0.2227 | 0.2387 | 0.2063 | 0.2140 | 0.2253 | 0.2407 | 0.2040 | 0.2113 | 0.2203 | 0.2517 | 0.2090 | 0.2160 | 0.2340 | 0.2487 | 0.2163 | 0.2057 | 0.2353 | 0.2757 |
| STDEV | 0.00417931 | 0.00913601 | 0.0075056 | 0.001 | 0.0025166 | 0.0037859 | 0.0102632 | 0.0036056 | 0.0045092 | 0.00152753 | 0.004 | 0.001528 | 0.000577 | 0.002082 | 0.003606 | 0.003 | 0.002646 | 0.002887 | 0.003786 | 0.006028 | 0.006351 | 0.002517 |
| | | | | | | | | | | | | | | | | | | | | | | |
| | | | | | | | | | | | | | | | | | | | | | | |
| | | | | | | | | | | | | | | | | | | | | | | |
| | | | W | | | | 30E | | | | 50E | | | | 70E | | | | 100E | | | |
| | NT | LPS | 25 | 50 | 100 | 200 | 25 | 50 | 100 | 200 | 25 | 50 | 100 | 200 | 25 | 50 | 100 | 200 | 25 | 50 | 100 | 200 |
| uM | 4.2500 | 20.3370 | 19.3587 | 20.9891 | 21.9674 | 23.4891 | 18.9239 | 21.3152 | 21.7500 | 23.7065 | 19.4674 | 20.8804 | 21.6413 | 25.3370 | 20.3370 | 21.2065 | 23.2717 | 24.5761 | 20.7717 | 20.0109 | 23.7065 | 27.4022 |
| | 4.9022 | 20.8804 | 20.9891 | 20.8804 | 22.1848 | 24.1413 | 21.0978 | 20.5543 | 22.7283 | 23.9239 | 20.3370 | 20.5543 | 21.7500 | 25.0109 | 20.8804 | 21.5326 | 23.3804 | 24.5761 | 21.5326 | 20.7717 | 23.7065 | 27.7283 |
| | 4.7935 | 20.6630 | 20.2283 | 21.0978 | 21.6413 | 23.3804 | 20.4457 | 21.0978 | 22.1848 | 24.0326 | 19.9022 | 20.6630 | 21.6413 | 24.9022 | 20.1196 | 20.8804 | 22.8370 | 25.1196 | 21.4239 | 19.4674 | 22.5109 | 27.9457 |
| | 3.9239 | 21.9674 | | | | | | | | | | | | | | | | | | | | |
| | 3.9239 | 22.5109 | | | | | | | | | | | | | | | | | | | | |
| | 3.9239 | 22.6196 | | | | | | | | | | | | | | | | | | | | |
| AVERAGE | 4.2862 | 21.4964 | 20.1920 | 20.9891 | 21.9312 | 23.6703 | 20.1558 | 20.9891 | 22.2210 | 23.8877 | 19.9022 | 20.6993 | 21.6775 | 25.0833 | 20.4457 | 21.2065 | 23.1630 | 24.7572 | 21.2428 | 20.0833 | 23.3080 | 27.6920 |
| STDEV | 0.45427328 | 0.99304449 | 0.815821 | 0.108696 | 0.2735447 | 0.4115151 | 1.1155655 | 0.3919077 | 0.4901358 | 0.16603535 | 0.434783 | 0.166035 | 0.062755 | 0.226268 | 0.391908 | 0.326087 | 0.287582 | 0.313777 | 0.411515 | 0.655186 | 0.69031 | 0.273545 |
### Chart:
| Category | |
|---|---|

## Slide 3
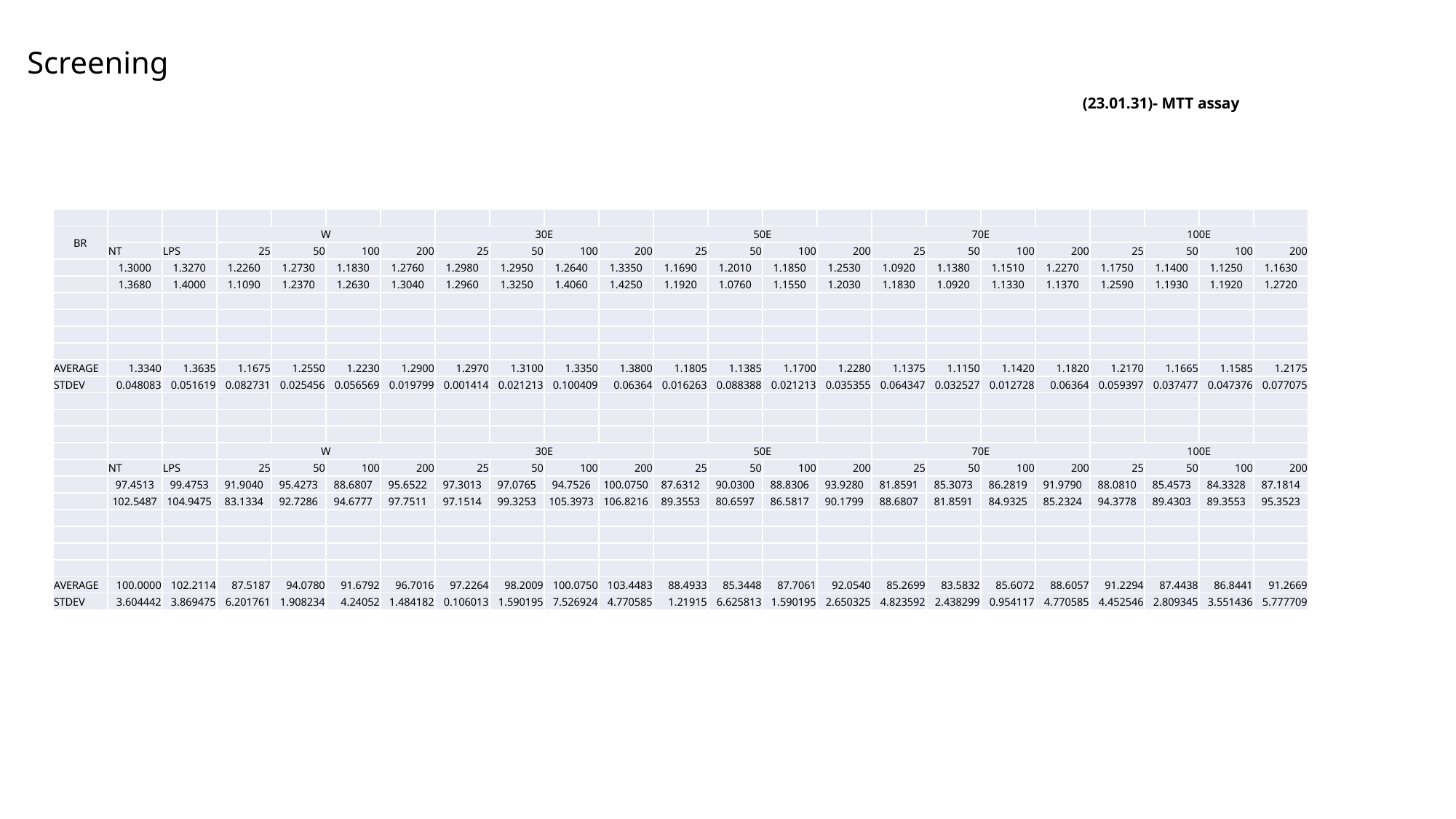

# Screening
(23.01.31)- MTT assay
| | | | | | | | | | | | | | | | | | | | | | | |
| --- | --- | --- | --- | --- | --- | --- | --- | --- | --- | --- | --- | --- | --- | --- | --- | --- | --- | --- | --- | --- | --- | --- |
| BR | | | W | | | | 30E | | | | 50E | | | | 70E | | | | 100E | | | |
| | NT | LPS | 25 | 50 | 100 | 200 | 25 | 50 | 100 | 200 | 25 | 50 | 100 | 200 | 25 | 50 | 100 | 200 | 25 | 50 | 100 | 200 |
| | 1.3000 | 1.3270 | 1.2260 | 1.2730 | 1.1830 | 1.2760 | 1.2980 | 1.2950 | 1.2640 | 1.3350 | 1.1690 | 1.2010 | 1.1850 | 1.2530 | 1.0920 | 1.1380 | 1.1510 | 1.2270 | 1.1750 | 1.1400 | 1.1250 | 1.1630 |
| | 1.3680 | 1.4000 | 1.1090 | 1.2370 | 1.2630 | 1.3040 | 1.2960 | 1.3250 | 1.4060 | 1.4250 | 1.1920 | 1.0760 | 1.1550 | 1.2030 | 1.1830 | 1.0920 | 1.1330 | 1.1370 | 1.2590 | 1.1930 | 1.1920 | 1.2720 |
| | | | | | | | | | | | | | | | | | | | | | | |
| | | | | | | | | | | | | | | | | | | | | | | |
| | | | | | | | | | | | | | | | | | | | | | | |
| | | | | | | | | | | | | | | | | | | | | | | |
| AVERAGE | 1.3340 | 1.3635 | 1.1675 | 1.2550 | 1.2230 | 1.2900 | 1.2970 | 1.3100 | 1.3350 | 1.3800 | 1.1805 | 1.1385 | 1.1700 | 1.2280 | 1.1375 | 1.1150 | 1.1420 | 1.1820 | 1.2170 | 1.1665 | 1.1585 | 1.2175 |
| STDEV | 0.048083 | 0.051619 | 0.082731 | 0.025456 | 0.056569 | 0.019799 | 0.001414 | 0.021213 | 0.100409 | 0.06364 | 0.016263 | 0.088388 | 0.021213 | 0.035355 | 0.064347 | 0.032527 | 0.012728 | 0.06364 | 0.059397 | 0.037477 | 0.047376 | 0.077075 |
| | | | | | | | | | | | | | | | | | | | | | | |
| | | | | | | | | | | | | | | | | | | | | | | |
| | | | | | | | | | | | | | | | | | | | | | | |
| | | | W | | | | 30E | | | | 50E | | | | 70E | | | | 100E | | | |
| | NT | LPS | 25 | 50 | 100 | 200 | 25 | 50 | 100 | 200 | 25 | 50 | 100 | 200 | 25 | 50 | 100 | 200 | 25 | 50 | 100 | 200 |
| | 97.4513 | 99.4753 | 91.9040 | 95.4273 | 88.6807 | 95.6522 | 97.3013 | 97.0765 | 94.7526 | 100.0750 | 87.6312 | 90.0300 | 88.8306 | 93.9280 | 81.8591 | 85.3073 | 86.2819 | 91.9790 | 88.0810 | 85.4573 | 84.3328 | 87.1814 |
| | 102.5487 | 104.9475 | 83.1334 | 92.7286 | 94.6777 | 97.7511 | 97.1514 | 99.3253 | 105.3973 | 106.8216 | 89.3553 | 80.6597 | 86.5817 | 90.1799 | 88.6807 | 81.8591 | 84.9325 | 85.2324 | 94.3778 | 89.4303 | 89.3553 | 95.3523 |
| | | | | | | | | | | | | | | | | | | | | | | |
| | | | | | | | | | | | | | | | | | | | | | | |
| | | | | | | | | | | | | | | | | | | | | | | |
| | | | | | | | | | | | | | | | | | | | | | | |
| AVERAGE | 100.0000 | 102.2114 | 87.5187 | 94.0780 | 91.6792 | 96.7016 | 97.2264 | 98.2009 | 100.0750 | 103.4483 | 88.4933 | 85.3448 | 87.7061 | 92.0540 | 85.2699 | 83.5832 | 85.6072 | 88.6057 | 91.2294 | 87.4438 | 86.8441 | 91.2669 |
| STDEV | 3.604442 | 3.869475 | 6.201761 | 1.908234 | 4.24052 | 1.484182 | 0.106013 | 1.590195 | 7.526924 | 4.770585 | 1.21915 | 6.625813 | 1.590195 | 2.650325 | 4.823592 | 2.438299 | 0.954117 | 4.770585 | 4.452546 | 2.809345 | 3.551436 | 5.777709 |

## Slide 4
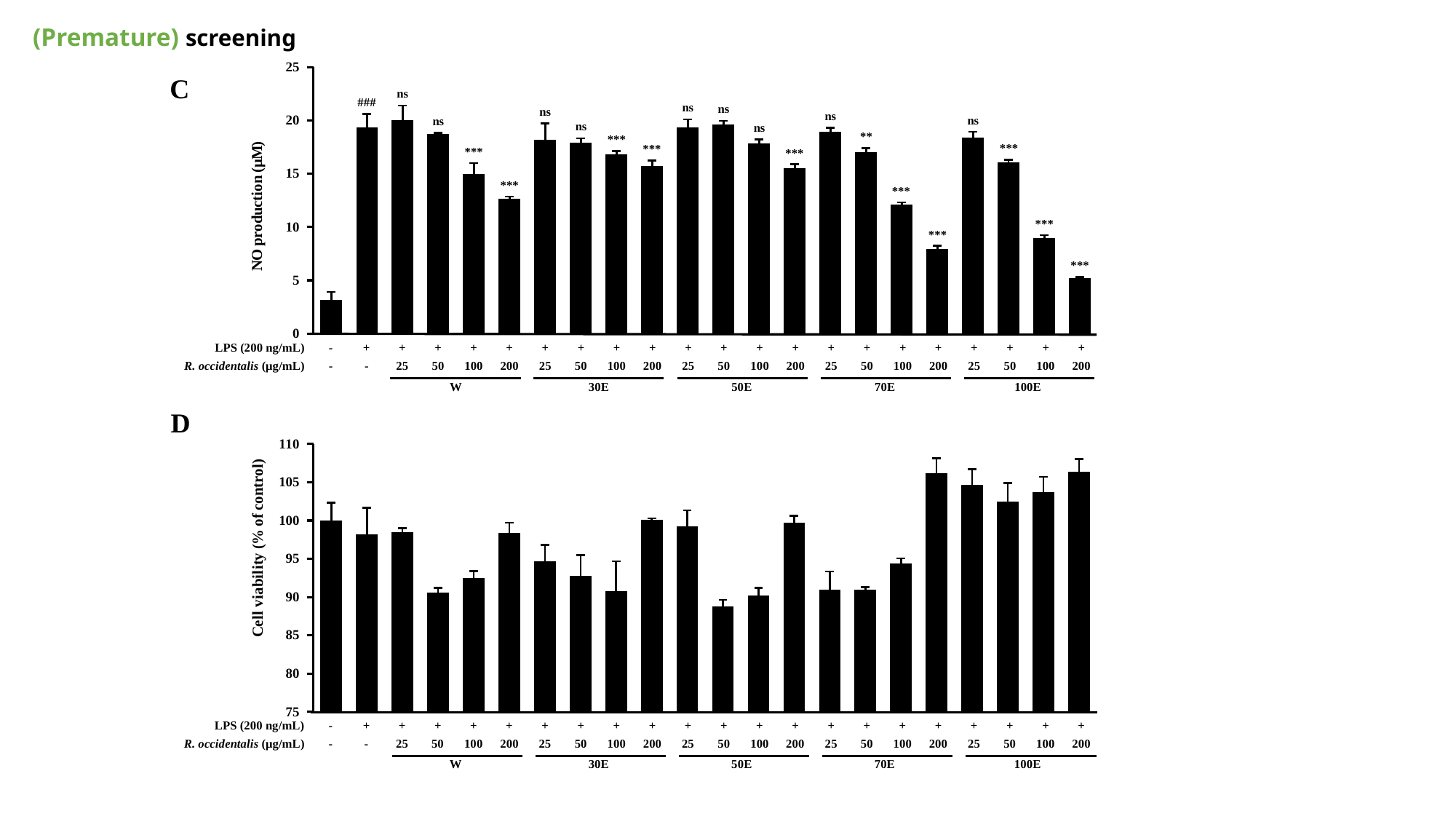

# (Premature) screening
### Chart
| Category | |
|---|---|
| NT | 3.174796747967479 |
| LPS | 19.353658536585368 |
| 25 | 20.04471544715447 |
| 50 | 18.703252032520325 |
| 100 | 15.004065040650405 |
| 200 | 12.64634146341463 |
| 25 | 18.174796747967477 |
| 50 | 17.89024390243902 |
| 100 | 16.79268292682927 |
| 200 | 15.735772357723576 |
| 25 | 19.313008130081297 |
| 50 | 19.597560975609753 |
| 100 | 17.849593495934958 |
| 200 | 15.53252032520325 |
| 25 | 18.947154471544714 |
| 50 | 16.995934959349594 |
| 100 | 12.117886178861786 |
| 200 | 7.971544715447152 |
| 25 | 18.378048780487802 |
| 50 | 16.060975609756095 |
| 100 | 8.947154471544716 |
| 200 | 5.2073170731707314 |ns
###
ns
ns
ns
ns
ns
ns
ns
ns
**
***
***
***
***
***
***
***
***
***
***
C
| LPS (200 ng/mL) | - | + | + | + | + | + | + | + | + | + | + | + | + | + | + | + | + | + | + | + | + | + |
| --- | --- | --- | --- | --- | --- | --- | --- | --- | --- | --- | --- | --- | --- | --- | --- | --- | --- | --- | --- | --- | --- | --- |
| R. occidentalis (μg/mL) | - | - | 25 | 50 | 100 | 200 | 25 | 50 | 100 | 200 | 25 | 50 | 100 | 200 | 25 | 50 | 100 | 200 | 25 | 50 | 100 | 200 |
| | | | W | | | | 30E | | | | 50E | | | | 70E | | | | 100E | | | |
D
### Chart
| Category | |
|---|---|
| NT | 100.0 |
| LPS | 98.22665267576076 |
| 25 | 98.48898216159496 |
| 50 | 90.61909758656873 |
| 100 | 92.46589716684157 |
| 200 | 98.40503672612802 |
| 25 | 94.66946484784891 |
| 50 | 92.75970619097586 |
| 100 | 90.76600209863591 |
| 200 | 100.08394543546694 |
| 25 | 99.2444910807975 |
| 50 | 88.75131164742918 |
| 100 | 90.22035676810076 |
| 200 | 99.74816369359917 |
| 25 | 90.9338929695698 |
| 50 | 90.95487932843652 |
| 100 | 94.41762854144808 |
| 200 | 106.16998950682058 |
| 25 | 104.70094438614903 |
| 50 | 102.43441762854145 |
| 100 | 103.73557187827912 |
| 200 | 106.35886673662121 || LPS (200 ng/mL) | - | + | + | + | + | + | + | + | + | + | + | + | + | + | + | + | + | + | + | + | + | + |
| --- | --- | --- | --- | --- | --- | --- | --- | --- | --- | --- | --- | --- | --- | --- | --- | --- | --- | --- | --- | --- | --- | --- |
| R. occidentalis (μg/mL) | - | - | 25 | 50 | 100 | 200 | 25 | 50 | 100 | 200 | 25 | 50 | 100 | 200 | 25 | 50 | 100 | 200 | 25 | 50 | 100 | 200 |
| | | | W | | | | 30E | | | | 50E | | | | 70E | | | | 100E | | | |

## Slide 5
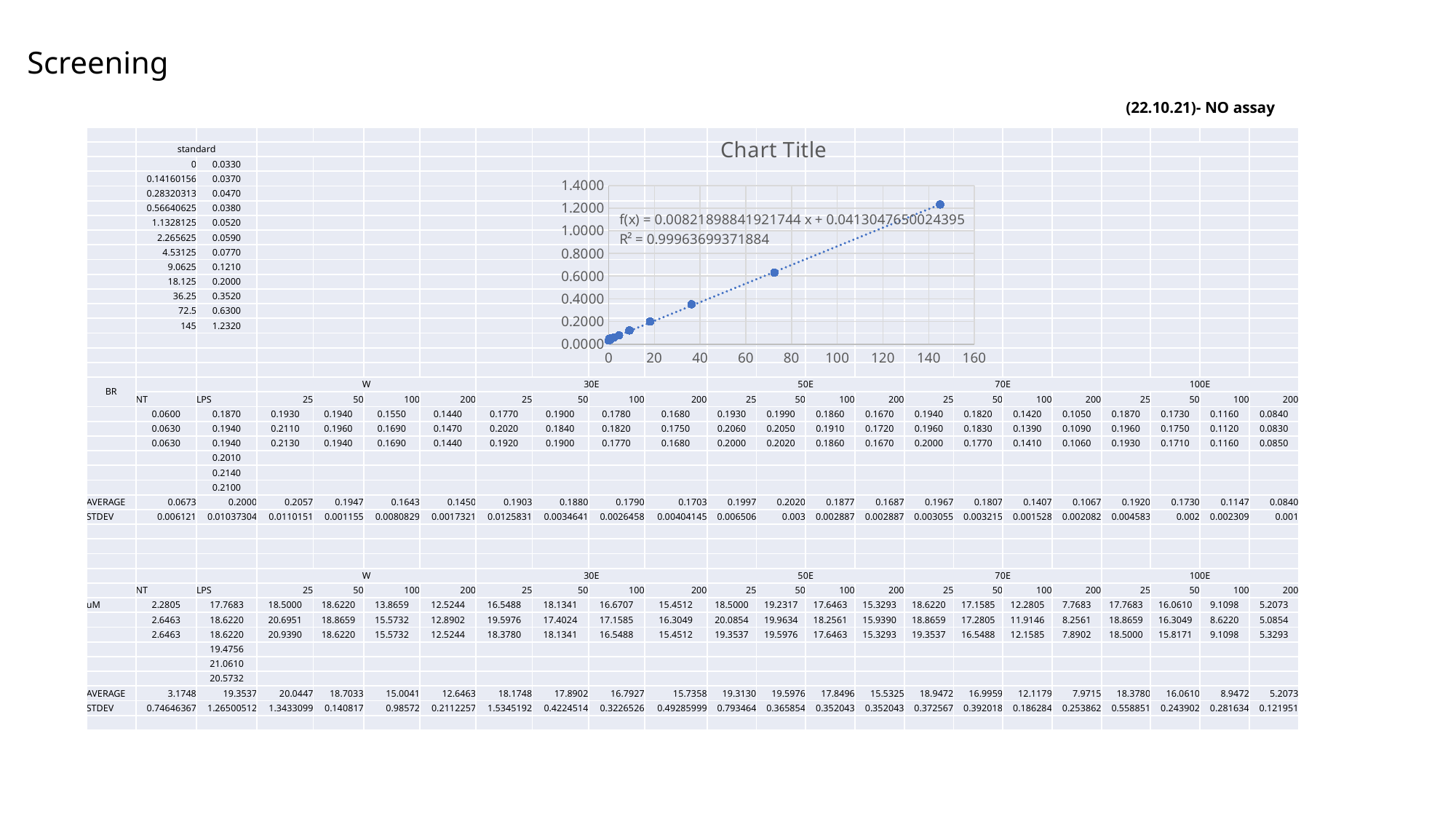

# Screening
(22.10.21)- NO assay
### Chart:
| Category | |
|---|---|| | | | | | | | | | | | | | | | | | | | | | | |
| --- | --- | --- | --- | --- | --- | --- | --- | --- | --- | --- | --- | --- | --- | --- | --- | --- | --- | --- | --- | --- | --- | --- |
| | standard | | | | | | | | | | | | | | | | | | | | | |
| | 0 | 0.0330 | | | | | | | | | | | | | | | | | | | | |
| | 0.14160156 | 0.0370 | | | | | | | | | | | | | | | | | | | | |
| | 0.28320313 | 0.0470 | | | | | | | | | | | | | | | | | | | | |
| | 0.56640625 | 0.0380 | | | | | | | | | | | | | | | | | | | | |
| | 1.1328125 | 0.0520 | | | | | | | | | | | | | | | | | | | | |
| | 2.265625 | 0.0590 | | | | | | | | | | | | | | | | | | | | |
| | 4.53125 | 0.0770 | | | | | | | | | | | | | | | | | | | | |
| | 9.0625 | 0.1210 | | | | | | | | | | | | | | | | | | | | |
| | 18.125 | 0.2000 | | | | | | | | | | | | | | | | | | | | |
| | 36.25 | 0.3520 | | | | | | | | | | | | | | | | | | | | |
| | 72.5 | 0.6300 | | | | | | | | | | | | | | | | | | | | |
| | 145 | 1.2320 | | | | | | | | | | | | | | | | | | | | |
| | | | | | | | | | | | | | | | | | | | | | | |
| | | | | | | | | | | | | | | | | | | | | | | |
| | | | | | | | | | | | | | | | | | | | | | | |
| BR | | | W | | | | 30E | | | | 50E | | | | 70E | | | | 100E | | | |
| | NT | LPS | 25 | 50 | 100 | 200 | 25 | 50 | 100 | 200 | 25 | 50 | 100 | 200 | 25 | 50 | 100 | 200 | 25 | 50 | 100 | 200 |
| | 0.0600 | 0.1870 | 0.1930 | 0.1940 | 0.1550 | 0.1440 | 0.1770 | 0.1900 | 0.1780 | 0.1680 | 0.1930 | 0.1990 | 0.1860 | 0.1670 | 0.1940 | 0.1820 | 0.1420 | 0.1050 | 0.1870 | 0.1730 | 0.1160 | 0.0840 |
| | 0.0630 | 0.1940 | 0.2110 | 0.1960 | 0.1690 | 0.1470 | 0.2020 | 0.1840 | 0.1820 | 0.1750 | 0.2060 | 0.2050 | 0.1910 | 0.1720 | 0.1960 | 0.1830 | 0.1390 | 0.1090 | 0.1960 | 0.1750 | 0.1120 | 0.0830 |
| | 0.0630 | 0.1940 | 0.2130 | 0.1940 | 0.1690 | 0.1440 | 0.1920 | 0.1900 | 0.1770 | 0.1680 | 0.2000 | 0.2020 | 0.1860 | 0.1670 | 0.2000 | 0.1770 | 0.1410 | 0.1060 | 0.1930 | 0.1710 | 0.1160 | 0.0850 |
| | | 0.2010 | | | | | | | | | | | | | | | | | | | | |
| | | 0.2140 | | | | | | | | | | | | | | | | | | | | |
| | | 0.2100 | | | | | | | | | | | | | | | | | | | | |
| AVERAGE | 0.0673 | 0.2000 | 0.2057 | 0.1947 | 0.1643 | 0.1450 | 0.1903 | 0.1880 | 0.1790 | 0.1703 | 0.1997 | 0.2020 | 0.1877 | 0.1687 | 0.1967 | 0.1807 | 0.1407 | 0.1067 | 0.1920 | 0.1730 | 0.1147 | 0.0840 |
| STDEV | 0.006121 | 0.01037304 | 0.0110151 | 0.001155 | 0.0080829 | 0.0017321 | 0.0125831 | 0.0034641 | 0.0026458 | 0.00404145 | 0.006506 | 0.003 | 0.002887 | 0.002887 | 0.003055 | 0.003215 | 0.001528 | 0.002082 | 0.004583 | 0.002 | 0.002309 | 0.001 |
| | | | | | | | | | | | | | | | | | | | | | | |
| | | | | | | | | | | | | | | | | | | | | | | |
| | | | | | | | | | | | | | | | | | | | | | | |
| | | | W | | | | 30E | | | | 50E | | | | 70E | | | | 100E | | | |
| | NT | LPS | 25 | 50 | 100 | 200 | 25 | 50 | 100 | 200 | 25 | 50 | 100 | 200 | 25 | 50 | 100 | 200 | 25 | 50 | 100 | 200 |
| uM | 2.2805 | 17.7683 | 18.5000 | 18.6220 | 13.8659 | 12.5244 | 16.5488 | 18.1341 | 16.6707 | 15.4512 | 18.5000 | 19.2317 | 17.6463 | 15.3293 | 18.6220 | 17.1585 | 12.2805 | 7.7683 | 17.7683 | 16.0610 | 9.1098 | 5.2073 |
| | 2.6463 | 18.6220 | 20.6951 | 18.8659 | 15.5732 | 12.8902 | 19.5976 | 17.4024 | 17.1585 | 16.3049 | 20.0854 | 19.9634 | 18.2561 | 15.9390 | 18.8659 | 17.2805 | 11.9146 | 8.2561 | 18.8659 | 16.3049 | 8.6220 | 5.0854 |
| | 2.6463 | 18.6220 | 20.9390 | 18.6220 | 15.5732 | 12.5244 | 18.3780 | 18.1341 | 16.5488 | 15.4512 | 19.3537 | 19.5976 | 17.6463 | 15.3293 | 19.3537 | 16.5488 | 12.1585 | 7.8902 | 18.5000 | 15.8171 | 9.1098 | 5.3293 |
| | | 19.4756 | | | | | | | | | | | | | | | | | | | | |
| | | 21.0610 | | | | | | | | | | | | | | | | | | | | |
| | | 20.5732 | | | | | | | | | | | | | | | | | | | | |
| AVERAGE | 3.1748 | 19.3537 | 20.0447 | 18.7033 | 15.0041 | 12.6463 | 18.1748 | 17.8902 | 16.7927 | 15.7358 | 19.3130 | 19.5976 | 17.8496 | 15.5325 | 18.9472 | 16.9959 | 12.1179 | 7.9715 | 18.3780 | 16.0610 | 8.9472 | 5.2073 |
| STDEV | 0.74646367 | 1.26500512 | 1.3433099 | 0.140817 | 0.98572 | 0.2112257 | 1.5345192 | 0.4224514 | 0.3226526 | 0.49285999 | 0.793464 | 0.365854 | 0.352043 | 0.352043 | 0.372567 | 0.392018 | 0.186284 | 0.253862 | 0.558851 | 0.243902 | 0.281634 | 0.121951 |
| | | | | | | | | | | | | | | | | | | | | | | |

## Slide 6
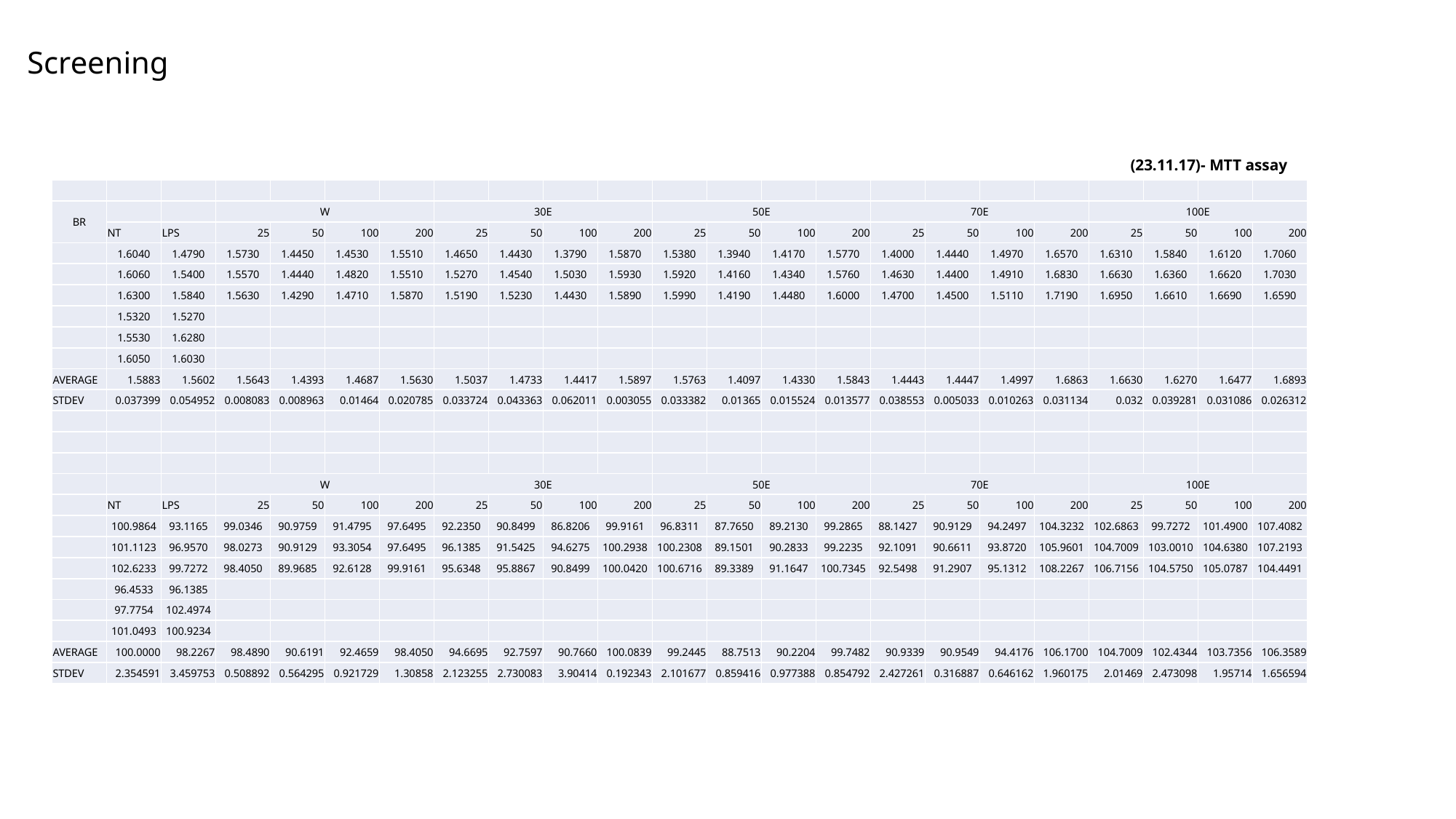

Screening
(23.11.17)- MTT assay
| | | | | | | | | | | | | | | | | | | | | | | |
| --- | --- | --- | --- | --- | --- | --- | --- | --- | --- | --- | --- | --- | --- | --- | --- | --- | --- | --- | --- | --- | --- | --- |
| BR | | | W | | | | 30E | | | | 50E | | | | 70E | | | | 100E | | | |
| | NT | LPS | 25 | 50 | 100 | 200 | 25 | 50 | 100 | 200 | 25 | 50 | 100 | 200 | 25 | 50 | 100 | 200 | 25 | 50 | 100 | 200 |
| | 1.6040 | 1.4790 | 1.5730 | 1.4450 | 1.4530 | 1.5510 | 1.4650 | 1.4430 | 1.3790 | 1.5870 | 1.5380 | 1.3940 | 1.4170 | 1.5770 | 1.4000 | 1.4440 | 1.4970 | 1.6570 | 1.6310 | 1.5840 | 1.6120 | 1.7060 |
| | 1.6060 | 1.5400 | 1.5570 | 1.4440 | 1.4820 | 1.5510 | 1.5270 | 1.4540 | 1.5030 | 1.5930 | 1.5920 | 1.4160 | 1.4340 | 1.5760 | 1.4630 | 1.4400 | 1.4910 | 1.6830 | 1.6630 | 1.6360 | 1.6620 | 1.7030 |
| | 1.6300 | 1.5840 | 1.5630 | 1.4290 | 1.4710 | 1.5870 | 1.5190 | 1.5230 | 1.4430 | 1.5890 | 1.5990 | 1.4190 | 1.4480 | 1.6000 | 1.4700 | 1.4500 | 1.5110 | 1.7190 | 1.6950 | 1.6610 | 1.6690 | 1.6590 |
| | 1.5320 | 1.5270 | | | | | | | | | | | | | | | | | | | | |
| | 1.5530 | 1.6280 | | | | | | | | | | | | | | | | | | | | |
| | 1.6050 | 1.6030 | | | | | | | | | | | | | | | | | | | | |
| AVERAGE | 1.5883 | 1.5602 | 1.5643 | 1.4393 | 1.4687 | 1.5630 | 1.5037 | 1.4733 | 1.4417 | 1.5897 | 1.5763 | 1.4097 | 1.4330 | 1.5843 | 1.4443 | 1.4447 | 1.4997 | 1.6863 | 1.6630 | 1.6270 | 1.6477 | 1.6893 |
| STDEV | 0.037399 | 0.054952 | 0.008083 | 0.008963 | 0.01464 | 0.020785 | 0.033724 | 0.043363 | 0.062011 | 0.003055 | 0.033382 | 0.01365 | 0.015524 | 0.013577 | 0.038553 | 0.005033 | 0.010263 | 0.031134 | 0.032 | 0.039281 | 0.031086 | 0.026312 |
| | | | | | | | | | | | | | | | | | | | | | | |
| | | | | | | | | | | | | | | | | | | | | | | |
| | | | | | | | | | | | | | | | | | | | | | | |
| | | | W | | | | 30E | | | | 50E | | | | 70E | | | | 100E | | | |
| | NT | LPS | 25 | 50 | 100 | 200 | 25 | 50 | 100 | 200 | 25 | 50 | 100 | 200 | 25 | 50 | 100 | 200 | 25 | 50 | 100 | 200 |
| | 100.9864 | 93.1165 | 99.0346 | 90.9759 | 91.4795 | 97.6495 | 92.2350 | 90.8499 | 86.8206 | 99.9161 | 96.8311 | 87.7650 | 89.2130 | 99.2865 | 88.1427 | 90.9129 | 94.2497 | 104.3232 | 102.6863 | 99.7272 | 101.4900 | 107.4082 |
| | 101.1123 | 96.9570 | 98.0273 | 90.9129 | 93.3054 | 97.6495 | 96.1385 | 91.5425 | 94.6275 | 100.2938 | 100.2308 | 89.1501 | 90.2833 | 99.2235 | 92.1091 | 90.6611 | 93.8720 | 105.9601 | 104.7009 | 103.0010 | 104.6380 | 107.2193 |
| | 102.6233 | 99.7272 | 98.4050 | 89.9685 | 92.6128 | 99.9161 | 95.6348 | 95.8867 | 90.8499 | 100.0420 | 100.6716 | 89.3389 | 91.1647 | 100.7345 | 92.5498 | 91.2907 | 95.1312 | 108.2267 | 106.7156 | 104.5750 | 105.0787 | 104.4491 |
| | 96.4533 | 96.1385 | | | | | | | | | | | | | | | | | | | | |
| | 97.7754 | 102.4974 | | | | | | | | | | | | | | | | | | | | |
| | 101.0493 | 100.9234 | | | | | | | | | | | | | | | | | | | | |
| AVERAGE | 100.0000 | 98.2267 | 98.4890 | 90.6191 | 92.4659 | 98.4050 | 94.6695 | 92.7597 | 90.7660 | 100.0839 | 99.2445 | 88.7513 | 90.2204 | 99.7482 | 90.9339 | 90.9549 | 94.4176 | 106.1700 | 104.7009 | 102.4344 | 103.7356 | 106.3589 |
| STDEV | 2.354591 | 3.459753 | 0.508892 | 0.564295 | 0.921729 | 1.30858 | 2.123255 | 2.730083 | 3.90414 | 0.192343 | 2.101677 | 0.859416 | 0.977388 | 0.854792 | 2.427261 | 0.316887 | 0.646162 | 1.960175 | 2.01469 | 2.473098 | 1.95714 | 1.656594 |

## Slide 7
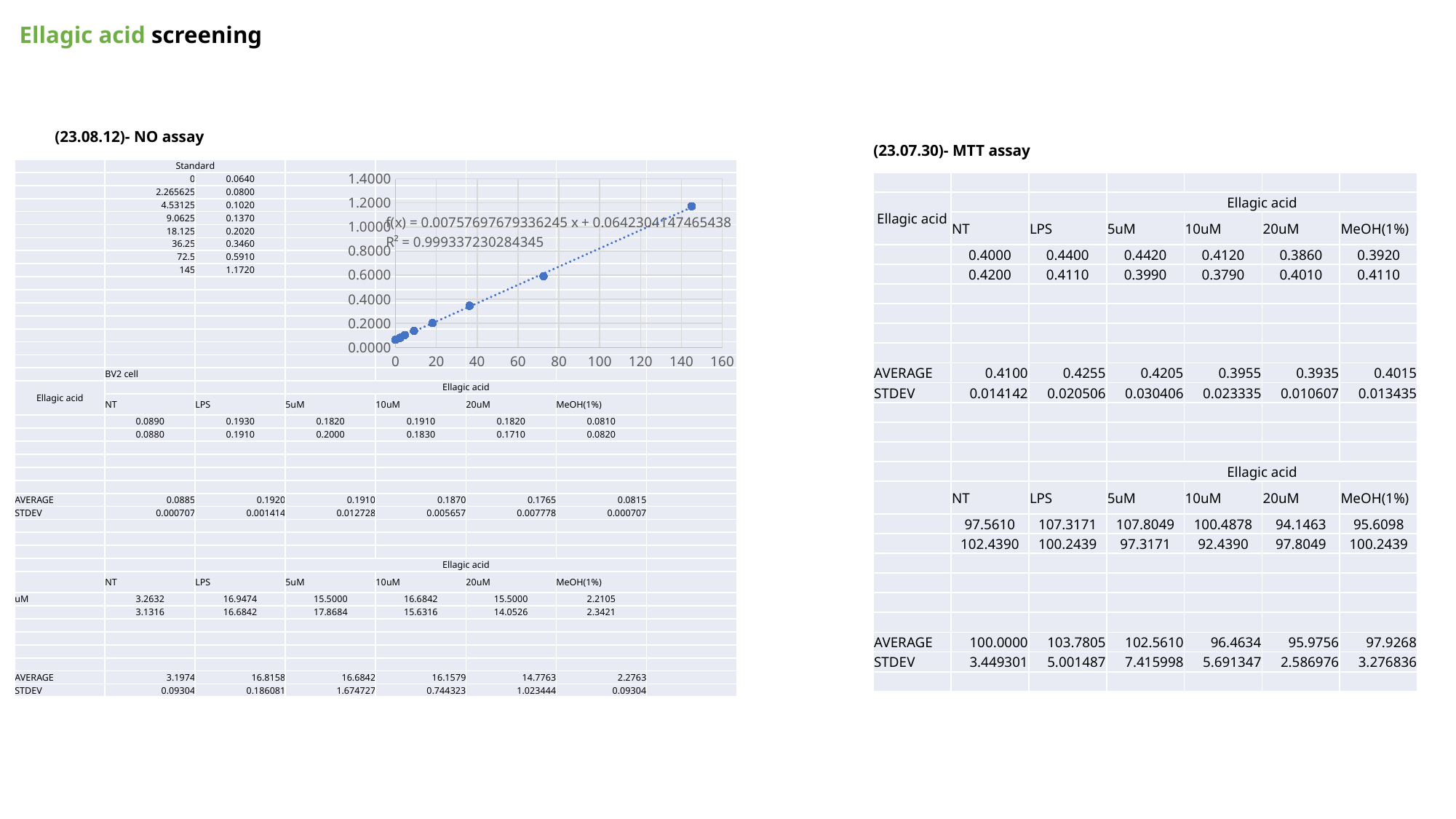

# Ellagic acid screening
(23.08.12)- NO assay
(23.07.30)- MTT assay
| | Standard | | | | | | |
| --- | --- | --- | --- | --- | --- | --- | --- |
| | 0 | 0.0640 | | | | | |
| | 2.265625 | 0.0800 | | | | | |
| | 4.53125 | 0.1020 | | | | | |
| | 9.0625 | 0.1370 | | | | | |
| | 18.125 | 0.2020 | | | | | |
| | 36.25 | 0.3460 | | | | | |
| | 72.5 | 0.5910 | | | | | |
| | 145 | 1.1720 | | | | | |
| | | | | | | | |
| | | | | | | | |
| | | | | | | | |
| | | | | | | | |
| | | | | | | | |
| | | | | | | | |
| | | | | | | | |
| | BV2 cell | | | | | | |
| Ellagic acid | | | Ellagic acid | | | | |
| | NT | LPS | 5uM | 10uM | 20uM | MeOH(1%) | |
| | 0.0890 | 0.1930 | 0.1820 | 0.1910 | 0.1820 | 0.0810 | |
| | 0.0880 | 0.1910 | 0.2000 | 0.1830 | 0.1710 | 0.0820 | |
| | | | | | | | |
| | | | | | | | |
| | | | | | | | |
| | | | | | | | |
| AVERAGE | 0.0885 | 0.1920 | 0.1910 | 0.1870 | 0.1765 | 0.0815 | |
| STDEV | 0.000707 | 0.001414 | 0.012728 | 0.005657 | 0.007778 | 0.000707 | |
| | | | | | | | |
| | | | | | | | |
| | | | | | | | |
| | | | Ellagic acid | | | | |
| | NT | LPS | 5uM | 10uM | 20uM | MeOH(1%) | |
| uM | 3.2632 | 16.9474 | 15.5000 | 16.6842 | 15.5000 | 2.2105 | |
| | 3.1316 | 16.6842 | 17.8684 | 15.6316 | 14.0526 | 2.3421 | |
| | | | | | | | |
| | | | | | | | |
| | | | | | | | |
| | | | | | | | |
| AVERAGE | 3.1974 | 16.8158 | 16.6842 | 16.1579 | 14.7763 | 2.2763 | |
| STDEV | 0.09304 | 0.186081 | 1.674727 | 0.744323 | 1.023444 | 0.09304 | |
### Chart
| Category | |
|---|---|| | | | | | | |
| --- | --- | --- | --- | --- | --- | --- |
| Ellagic acid | | | Ellagic acid | | | |
| | NT | LPS | 5uM | 10uM | 20uM | MeOH(1%) |
| | 0.4000 | 0.4400 | 0.4420 | 0.4120 | 0.3860 | 0.3920 |
| | 0.4200 | 0.4110 | 0.3990 | 0.3790 | 0.4010 | 0.4110 |
| | | | | | | |
| | | | | | | |
| | | | | | | |
| | | | | | | |
| AVERAGE | 0.4100 | 0.4255 | 0.4205 | 0.3955 | 0.3935 | 0.4015 |
| STDEV | 0.014142 | 0.020506 | 0.030406 | 0.023335 | 0.010607 | 0.013435 |
| | | | | | | |
| | | | | | | |
| | | | | | | |
| | | | Ellagic acid | | | |
| | NT | LPS | 5uM | 10uM | 20uM | MeOH(1%) |
| | 97.5610 | 107.3171 | 107.8049 | 100.4878 | 94.1463 | 95.6098 |
| | 102.4390 | 100.2439 | 97.3171 | 92.4390 | 97.8049 | 100.2439 |
| | | | | | | |
| | | | | | | |
| | | | | | | |
| | | | | | | |
| AVERAGE | 100.0000 | 103.7805 | 102.5610 | 96.4634 | 95.9756 | 97.9268 |
| STDEV | 3.449301 | 5.001487 | 7.415998 | 5.691347 | 2.586976 | 3.276836 |
| | | | | | | |

## Slide 8
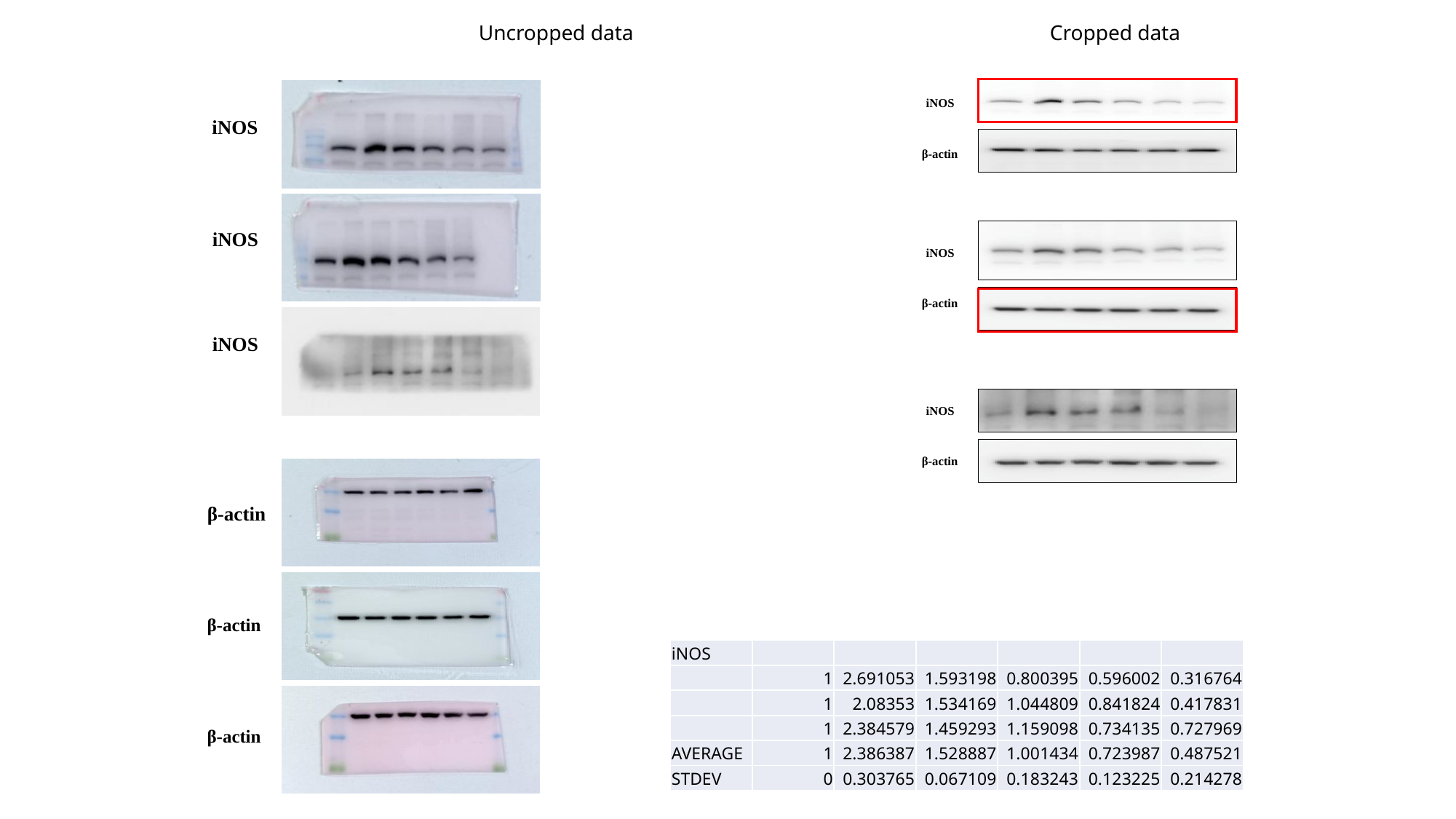

Uncropped data
Cropped data
iNOS
iNOS
β-actin
iNOS
iNOS
β-actin
iNOS
iNOS
β-actin
β-actin
β-actin
| iNOS | | | | | | |
| --- | --- | --- | --- | --- | --- | --- |
| | 1 | 2.691053 | 1.593198 | 0.800395 | 0.596002 | 0.316764 |
| | 1 | 2.08353 | 1.534169 | 1.044809 | 0.841824 | 0.417831 |
| | 1 | 2.384579 | 1.459293 | 1.159098 | 0.734135 | 0.727969 |
| AVERAGE | 1 | 2.386387 | 1.528887 | 1.001434 | 0.723987 | 0.487521 |
| STDEV | 0 | 0.303765 | 0.067109 | 0.183243 | 0.123225 | 0.214278 |
β-actin

## Slide 9
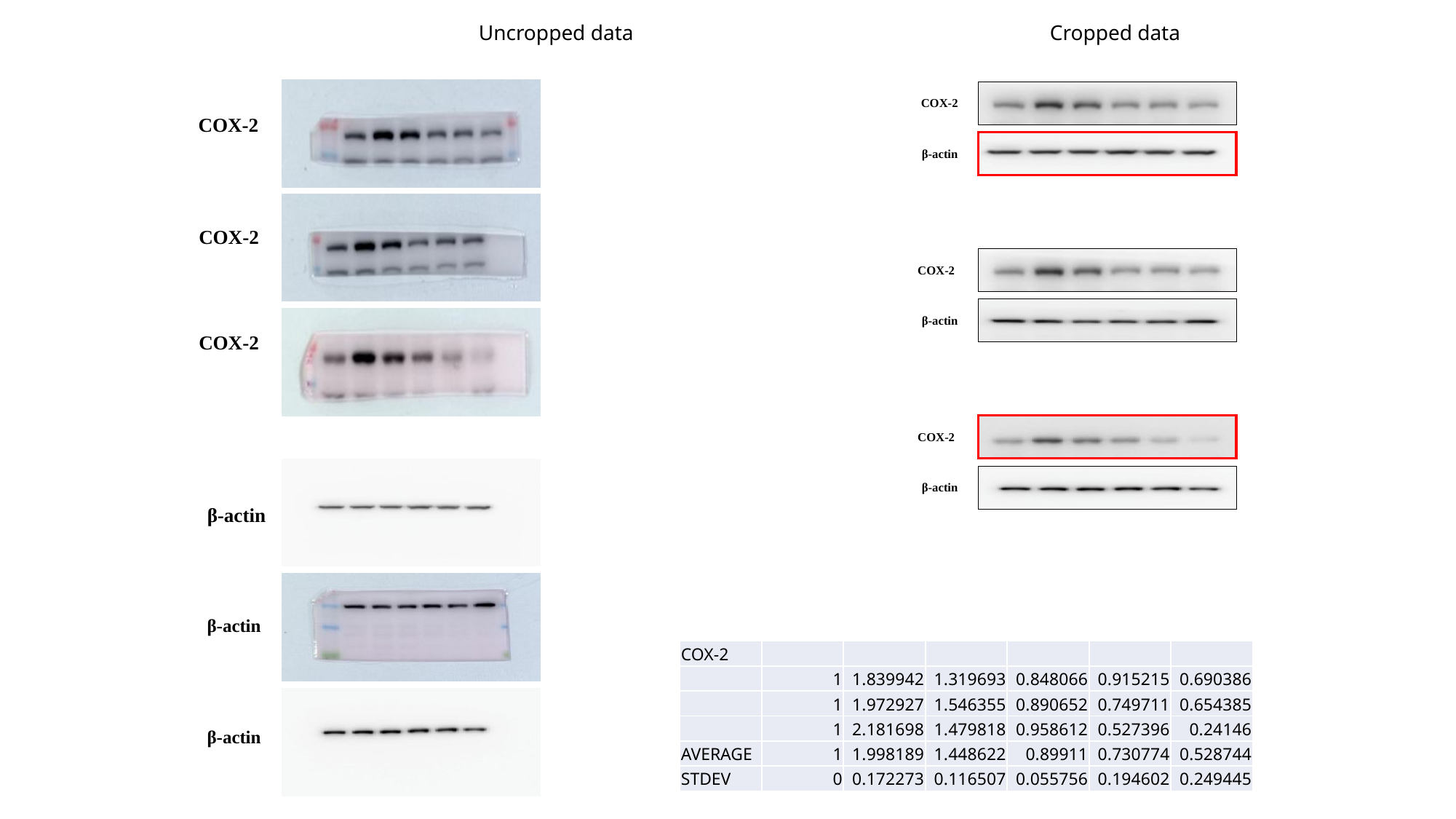

Uncropped data
Cropped data
COX-2
COX-2
β-actin
COX-2
COX-2
β-actin
COX-2
COX-2
β-actin
β-actin
β-actin
| COX-2 | | | | | | |
| --- | --- | --- | --- | --- | --- | --- |
| | 1 | 1.839942 | 1.319693 | 0.848066 | 0.915215 | 0.690386 |
| | 1 | 1.972927 | 1.546355 | 0.890652 | 0.749711 | 0.654385 |
| | 1 | 2.181698 | 1.479818 | 0.958612 | 0.527396 | 0.24146 |
| AVERAGE | 1 | 1.998189 | 1.448622 | 0.89911 | 0.730774 | 0.528744 |
| STDEV | 0 | 0.172273 | 0.116507 | 0.055756 | 0.194602 | 0.249445 |
β-actin

## Slide 10
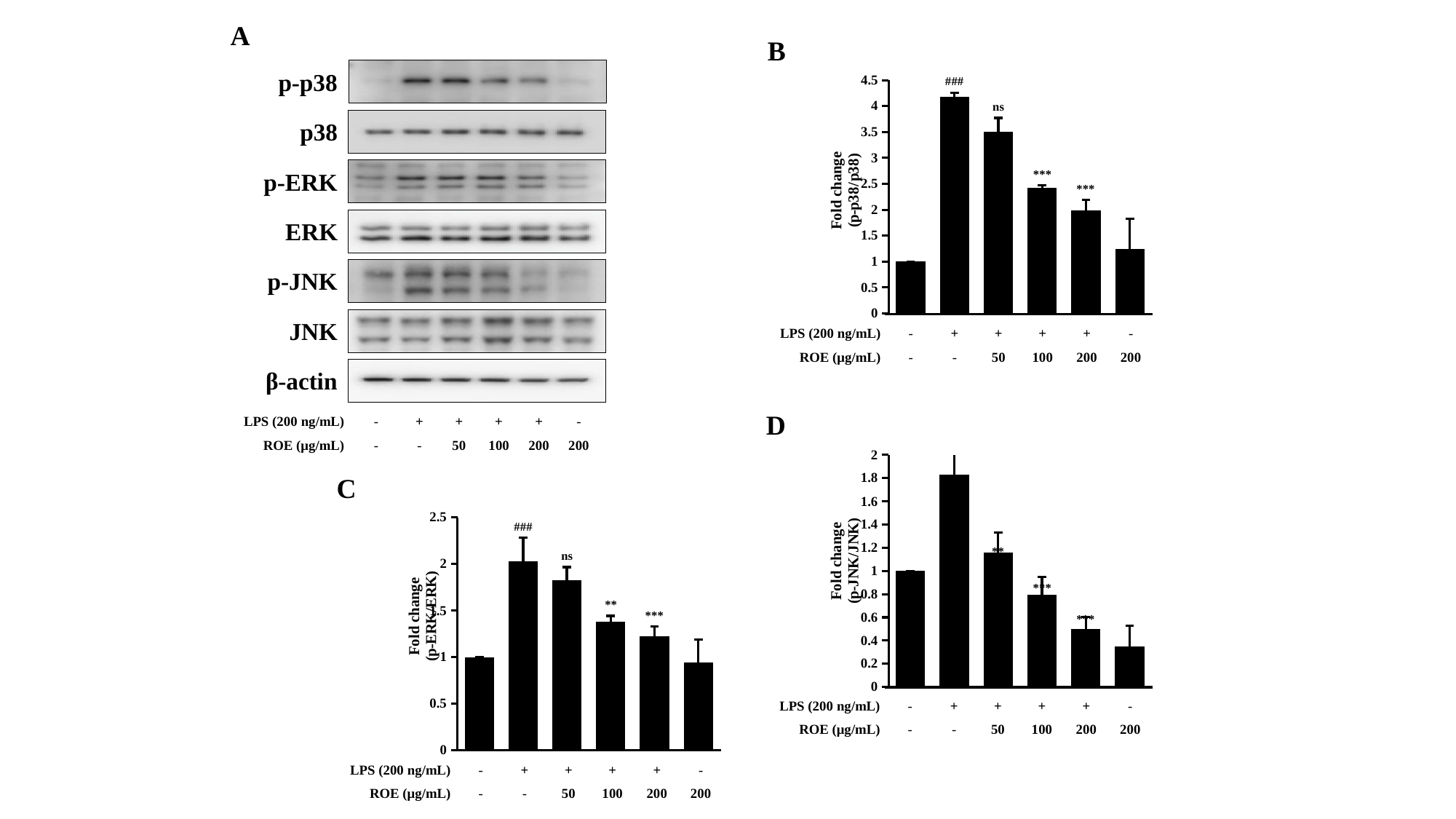

A
B
p-p38
### Chart
| Category | |
|---|---|
| | 1.0 |
| LPS | 4.175508963836182 |
| L50 | 3.50886211378935 |
| L100 | 2.4288386324658906 |
| L200 | 1.9850378684211558 |
| 200 | 1.246256621841048 |###
ns
p38
***
p-ERK
***
ERK
p-JNK
JNK
| LPS (200 ng/mL) | - | + | + | + | + | - |
| --- | --- | --- | --- | --- | --- | --- |
| ROE (μg/mL) | - | - | 50 | 100 | 200 | 200 |
β-actin
D
| LPS (200 ng/mL) | - | + | + | + | + | - |
| --- | --- | --- | --- | --- | --- | --- |
| ROE (μg/mL) | - | - | 50 | 100 | 200 | 200 |
### Chart
| Category | |
|---|---|
| NT | 1.0 |
| LPS | 1.8315297207501366 |
| L50 | 1.1587802635710356 |
| L100 | 0.7967961134264309 |
| L200 | 0.4979204171117018 |
| 200 | 0.3495347633621784 |C
###
### Chart
| Category | |
|---|---|
| NT | 1.0 |
| LPS | 2.031952048090764 |
| L50 | 1.8270430513316052 |
| L100 | 1.3787178772288555 |
| L200 | 1.2241201769511159 |
| 200 | 0.9443616588468652 |###
**
ns
***
**
***
***
| LPS (200 ng/mL) | - | + | + | + | + | - |
| --- | --- | --- | --- | --- | --- | --- |
| ROE (μg/mL) | - | - | 50 | 100 | 200 | 200 |
| LPS (200 ng/mL) | - | + | + | + | + | - |
| --- | --- | --- | --- | --- | --- | --- |
| ROE (μg/mL) | - | - | 50 | 100 | 200 | 200 |

## Slide 11
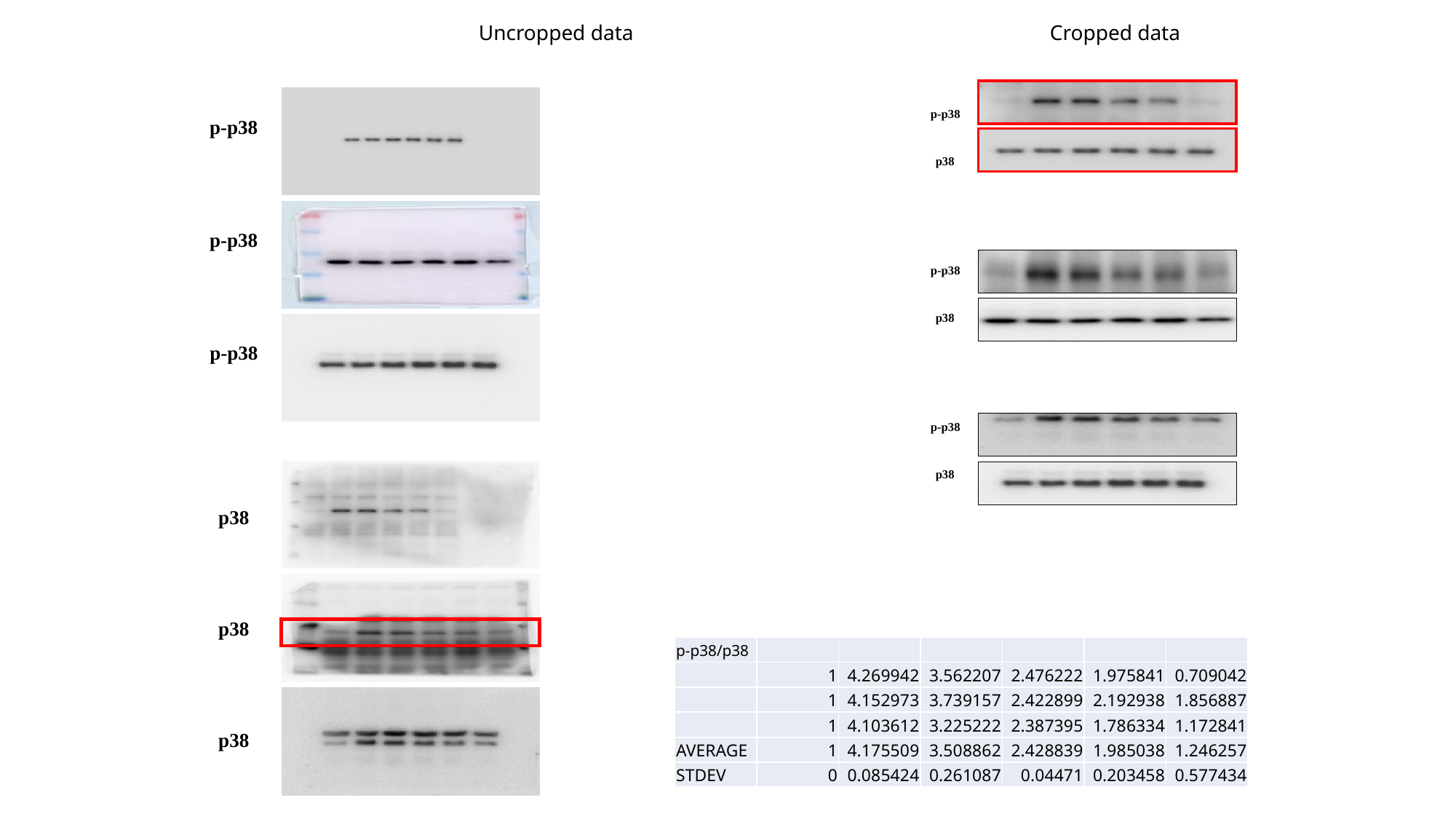

Uncropped data
Cropped data
p-p38
p-p38
p38
p-p38
p-p38
p38
p-p38
p-p38
p38
p38
p38
| p-p38/p38 | | | | | | |
| --- | --- | --- | --- | --- | --- | --- |
| | 1 | 4.269942 | 3.562207 | 2.476222 | 1.975841 | 0.709042 |
| | 1 | 4.152973 | 3.739157 | 2.422899 | 2.192938 | 1.856887 |
| | 1 | 4.103612 | 3.225222 | 2.387395 | 1.786334 | 1.172841 |
| AVERAGE | 1 | 4.175509 | 3.508862 | 2.428839 | 1.985038 | 1.246257 |
| STDEV | 0 | 0.085424 | 0.261087 | 0.04471 | 0.203458 | 0.577434 |
p38

## Slide 12
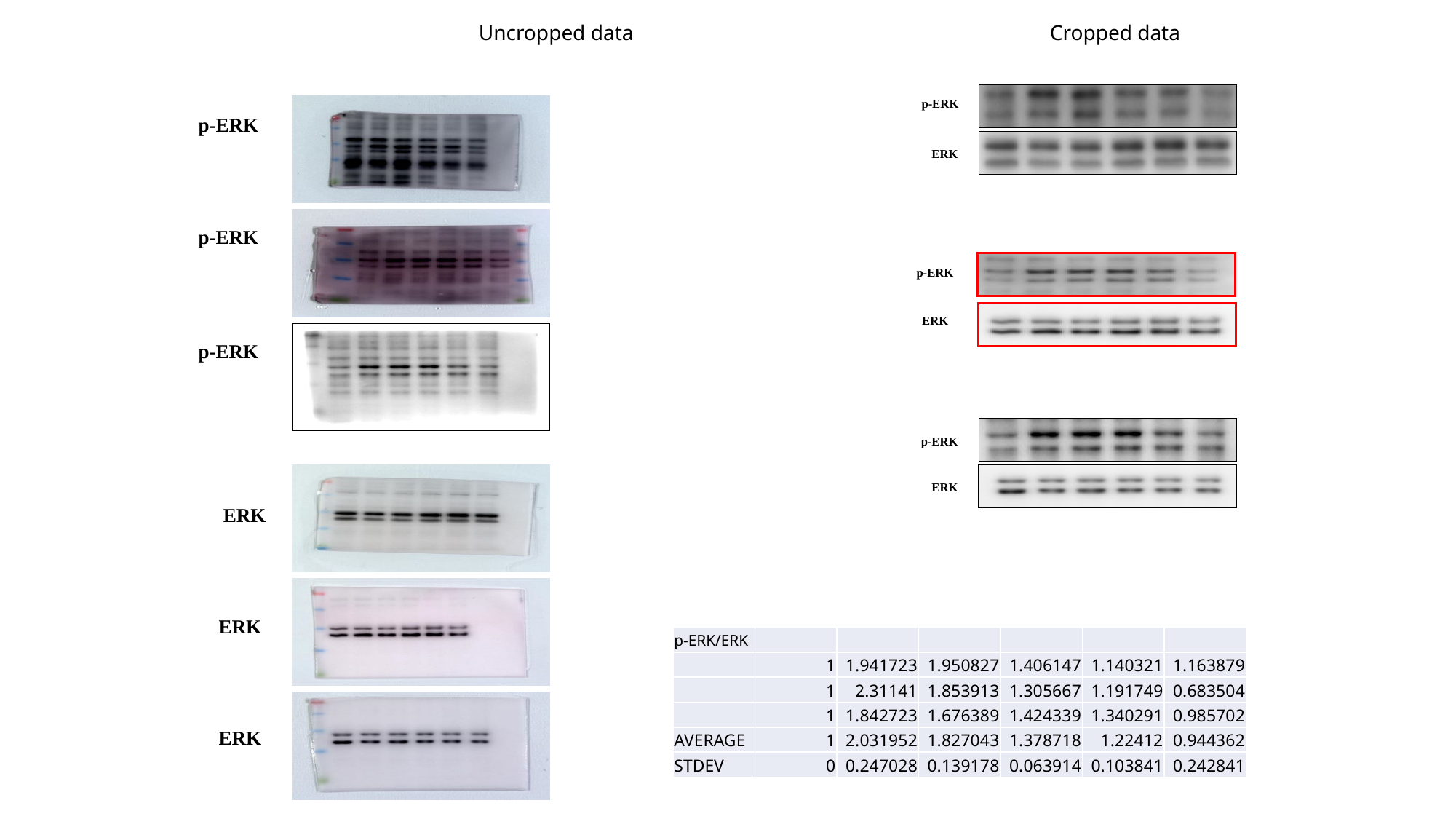

Uncropped data
Cropped data
p-ERK
p-ERK
ERK
p-ERK
p-ERK
ERK
p-ERK
p-ERK
ERK
ERK
ERK
| p-ERK/ERK | | | | | | |
| --- | --- | --- | --- | --- | --- | --- |
| | 1 | 1.941723 | 1.950827 | 1.406147 | 1.140321 | 1.163879 |
| | 1 | 2.31141 | 1.853913 | 1.305667 | 1.191749 | 0.683504 |
| | 1 | 1.842723 | 1.676389 | 1.424339 | 1.340291 | 0.985702 |
| AVERAGE | 1 | 2.031952 | 1.827043 | 1.378718 | 1.22412 | 0.944362 |
| STDEV | 0 | 0.247028 | 0.139178 | 0.063914 | 0.103841 | 0.242841 |
ERK

## Slide 13
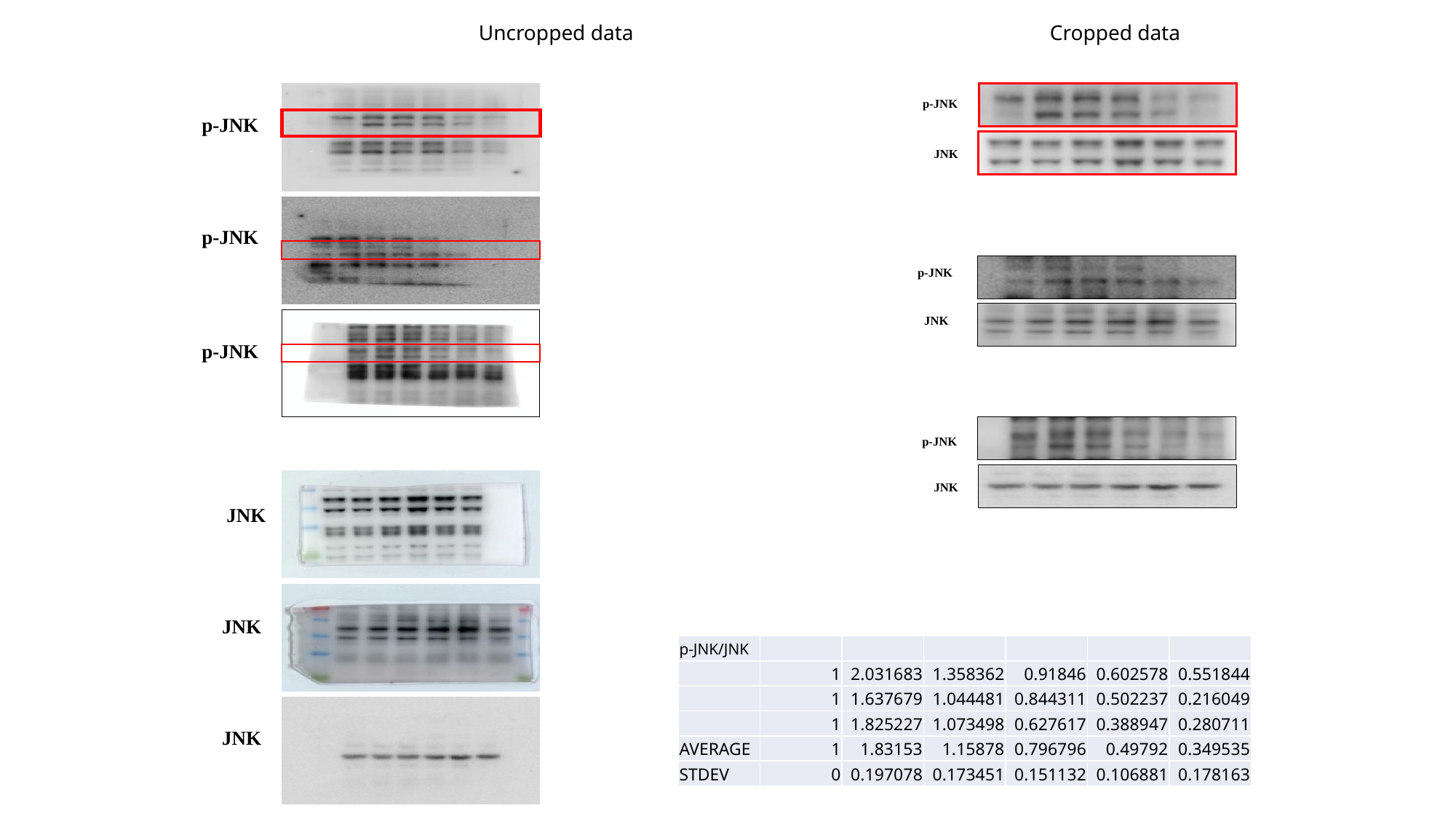

Uncropped data
Cropped data
p-JNK
p-JNK
JNK
p-JNK
p-JNK
JNK
p-JNK
p-JNK
JNK
JNK
JNK
| p-JNK/JNK | | | | | | |
| --- | --- | --- | --- | --- | --- | --- |
| | 1 | 2.031683 | 1.358362 | 0.91846 | 0.602578 | 0.551844 |
| | 1 | 1.637679 | 1.044481 | 0.844311 | 0.502237 | 0.216049 |
| | 1 | 1.825227 | 1.073498 | 0.627617 | 0.388947 | 0.280711 |
| AVERAGE | 1 | 1.83153 | 1.15878 | 0.796796 | 0.49792 | 0.349535 |
| STDEV | 0 | 0.197078 | 0.173451 | 0.151132 | 0.106881 | 0.178163 |
JNK

## Slide 14
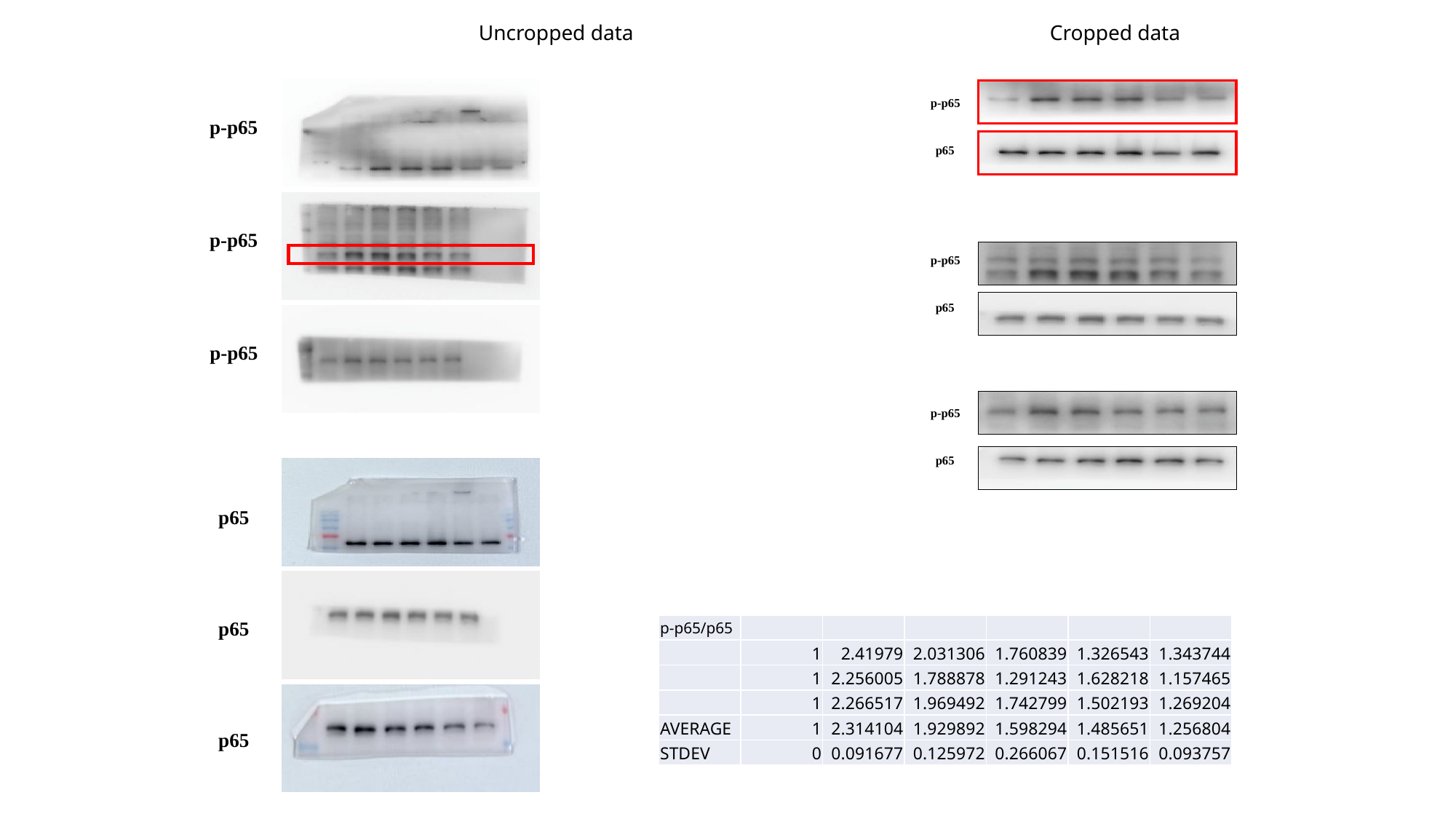

Uncropped data
Cropped data
p-p65
p-p65
p65
p-p65
p-p65
p65
p-p65
p-p65
p65
p65
p65
| p-p65/p65 | | | | | | |
| --- | --- | --- | --- | --- | --- | --- |
| | 1 | 2.41979 | 2.031306 | 1.760839 | 1.326543 | 1.343744 |
| | 1 | 2.256005 | 1.788878 | 1.291243 | 1.628218 | 1.157465 |
| | 1 | 2.266517 | 1.969492 | 1.742799 | 1.502193 | 1.269204 |
| AVERAGE | 1 | 2.314104 | 1.929892 | 1.598294 | 1.485651 | 1.256804 |
| STDEV | 0 | 0.091677 | 0.125972 | 0.266067 | 0.151516 | 0.093757 |
p65

## Slide 15
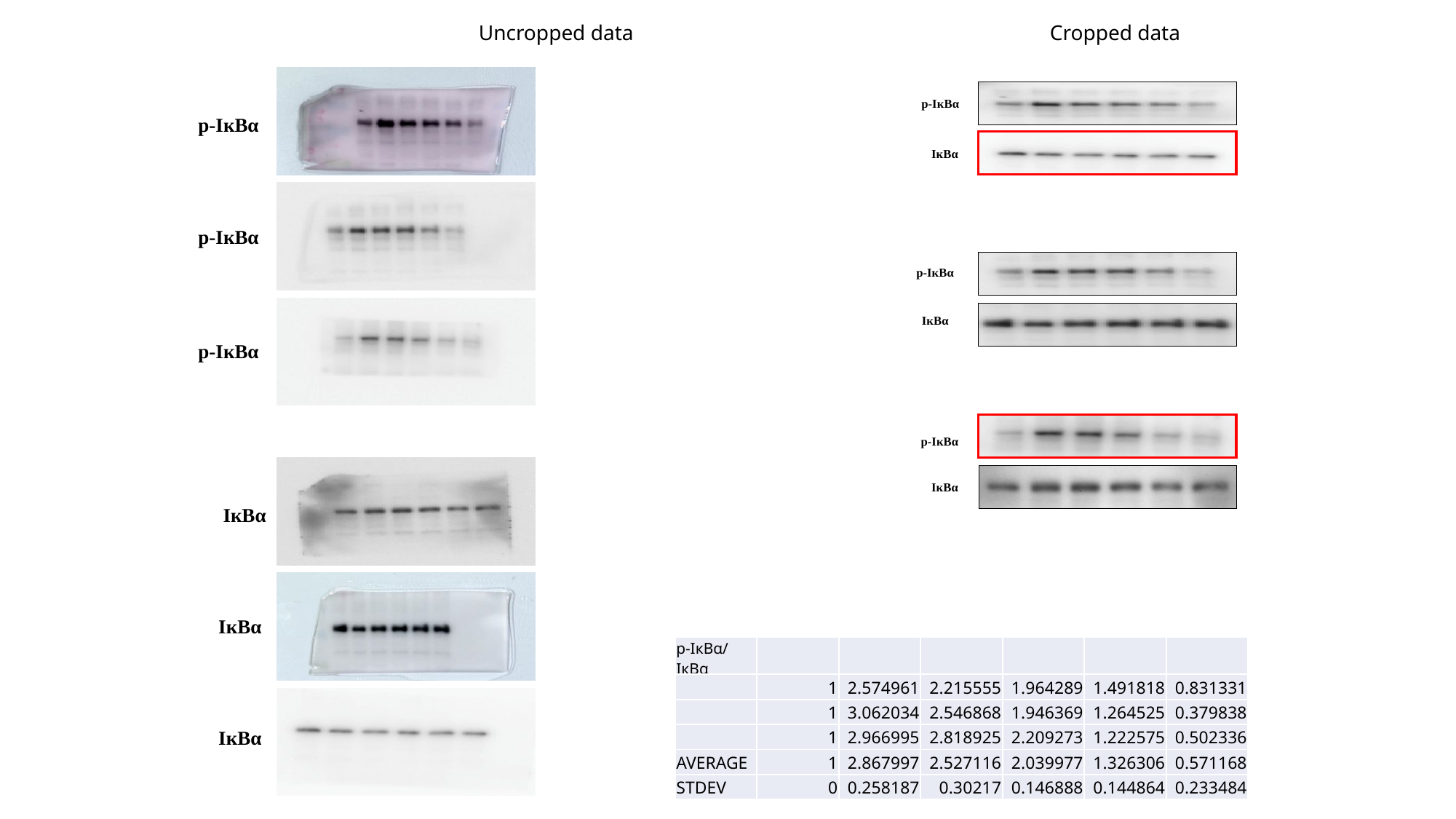

Uncropped data
Cropped data
p-IкBα
p-IкBα
IкBα
p-IкBα
p-IкBα
IкBα
p-IкBα
p-IкBα
IкBα
IкBα
IкBα
| p-IкBα/IкBα | | | | | | |
| --- | --- | --- | --- | --- | --- | --- |
| | 1 | 2.574961 | 2.215555 | 1.964289 | 1.491818 | 0.831331 |
| | 1 | 3.062034 | 2.546868 | 1.946369 | 1.264525 | 0.379838 |
| | 1 | 2.966995 | 2.818925 | 2.209273 | 1.222575 | 0.502336 |
| AVERAGE | 1 | 2.867997 | 2.527116 | 2.039977 | 1.326306 | 0.571168 |
| STDEV | 0 | 0.258187 | 0.30217 | 0.146888 | 0.144864 | 0.233484 |
IкBα

## Slide 16
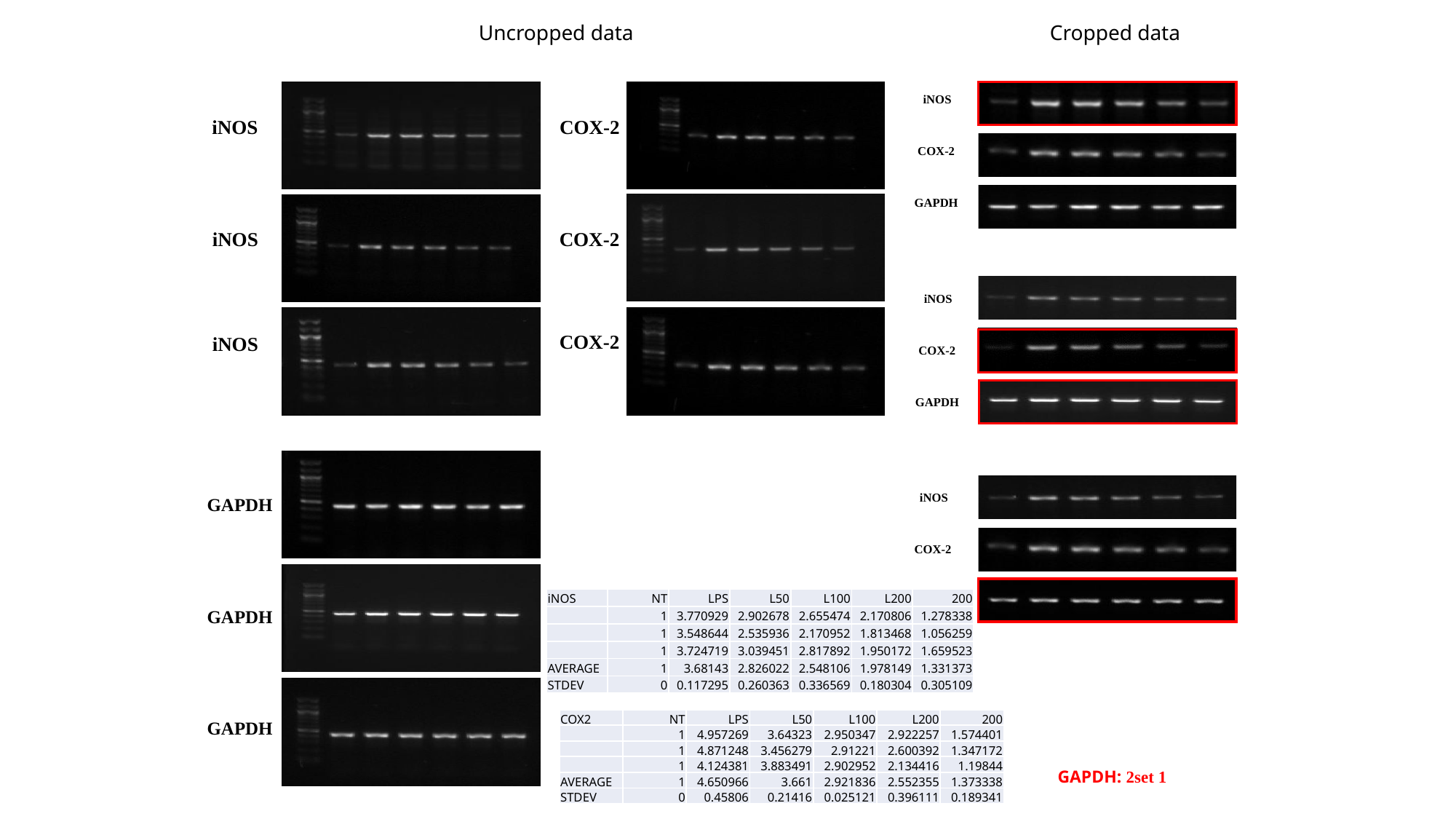

Uncropped data
Cropped data
iNOS
iNOS
COX-2
COX-2
GAPDH
iNOS
COX-2
iNOS
COX-2
iNOS
COX-2
GAPDH
iNOS
GAPDH
COX-2
GAPDH
| iNOS | NT | LPS | L50 | L100 | L200 | 200 |
| --- | --- | --- | --- | --- | --- | --- |
| | 1 | 3.770929 | 2.902678 | 2.655474 | 2.170806 | 1.278338 |
| | 1 | 3.548644 | 2.535936 | 2.170952 | 1.813468 | 1.056259 |
| | 1 | 3.724719 | 3.039451 | 2.817892 | 1.950172 | 1.659523 |
| AVERAGE | 1 | 3.68143 | 2.826022 | 2.548106 | 1.978149 | 1.331373 |
| STDEV | 0 | 0.117295 | 0.260363 | 0.336569 | 0.180304 | 0.305109 |
GAPDH
| COX2 | NT | LPS | L50 | L100 | L200 | 200 |
| --- | --- | --- | --- | --- | --- | --- |
| | 1 | 4.957269 | 3.64323 | 2.950347 | 2.922257 | 1.574401 |
| | 1 | 4.871248 | 3.456279 | 2.91221 | 2.600392 | 1.347172 |
| | 1 | 4.124381 | 3.883491 | 2.902952 | 2.134416 | 1.19844 |
| AVERAGE | 1 | 4.650966 | 3.661 | 2.921836 | 2.552355 | 1.373338 |
| STDEV | 0 | 0.45806 | 0.21416 | 0.025121 | 0.396111 | 0.189341 |
GAPDH
GAPDH: 2set 1

## Slide 17
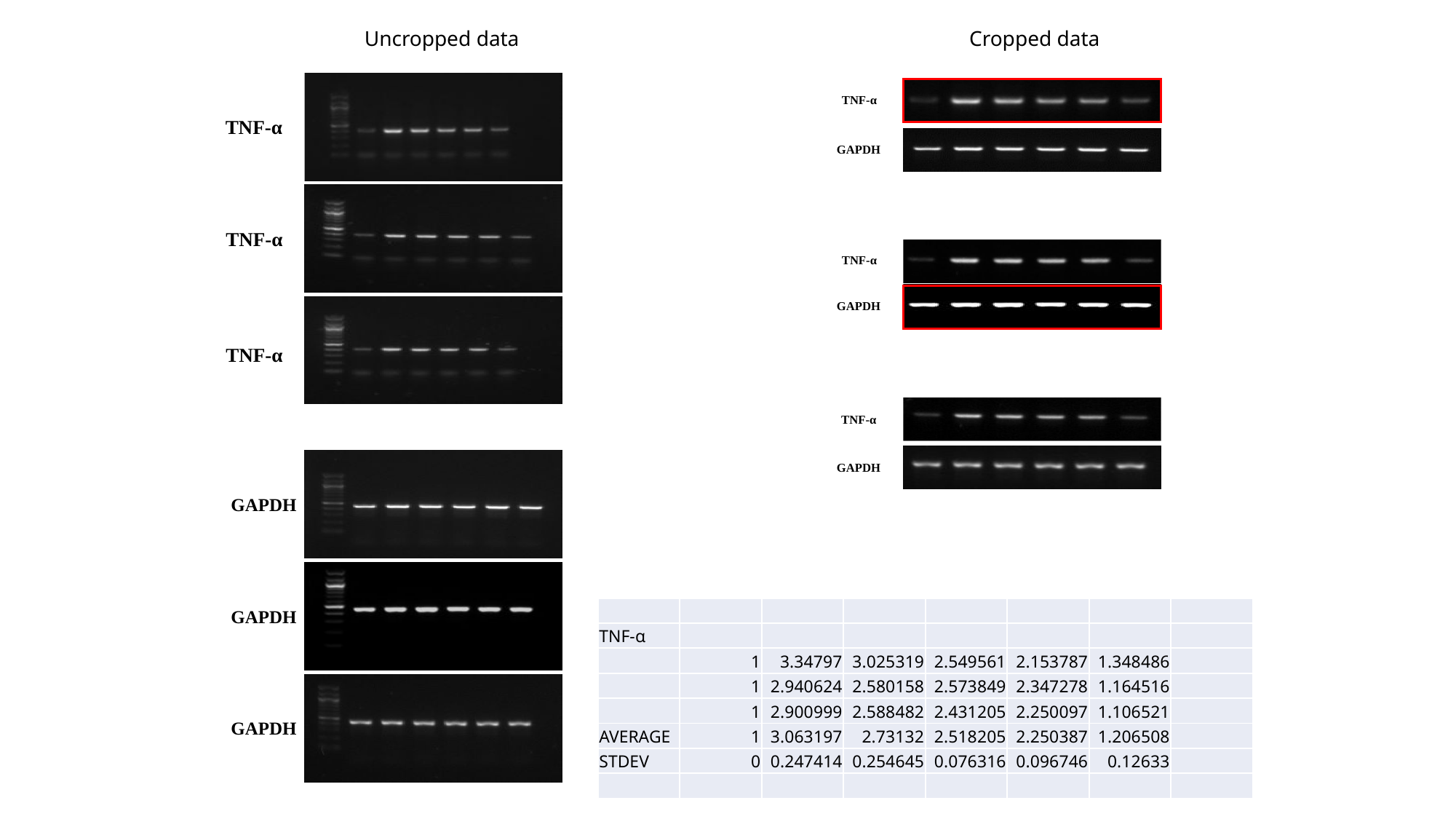

Uncropped data
Cropped data
TNF-α
TNF-α
GAPDH
TNF-α
TNF-α
GAPDH
TNF-α
TNF-α
GAPDH
GAPDH
| | | | | | | | |
| --- | --- | --- | --- | --- | --- | --- | --- |
| TNF-α | | | | | | | |
| | 1 | 3.34797 | 3.025319 | 2.549561 | 2.153787 | 1.348486 | |
| | 1 | 2.940624 | 2.580158 | 2.573849 | 2.347278 | 1.164516 | |
| | 1 | 2.900999 | 2.588482 | 2.431205 | 2.250097 | 1.106521 | |
| AVERAGE | 1 | 3.063197 | 2.73132 | 2.518205 | 2.250387 | 1.206508 | |
| STDEV | 0 | 0.247414 | 0.254645 | 0.076316 | 0.096746 | 0.12633 | |
| | | | | | | | |
GAPDH
GAPDH

## Slide 18
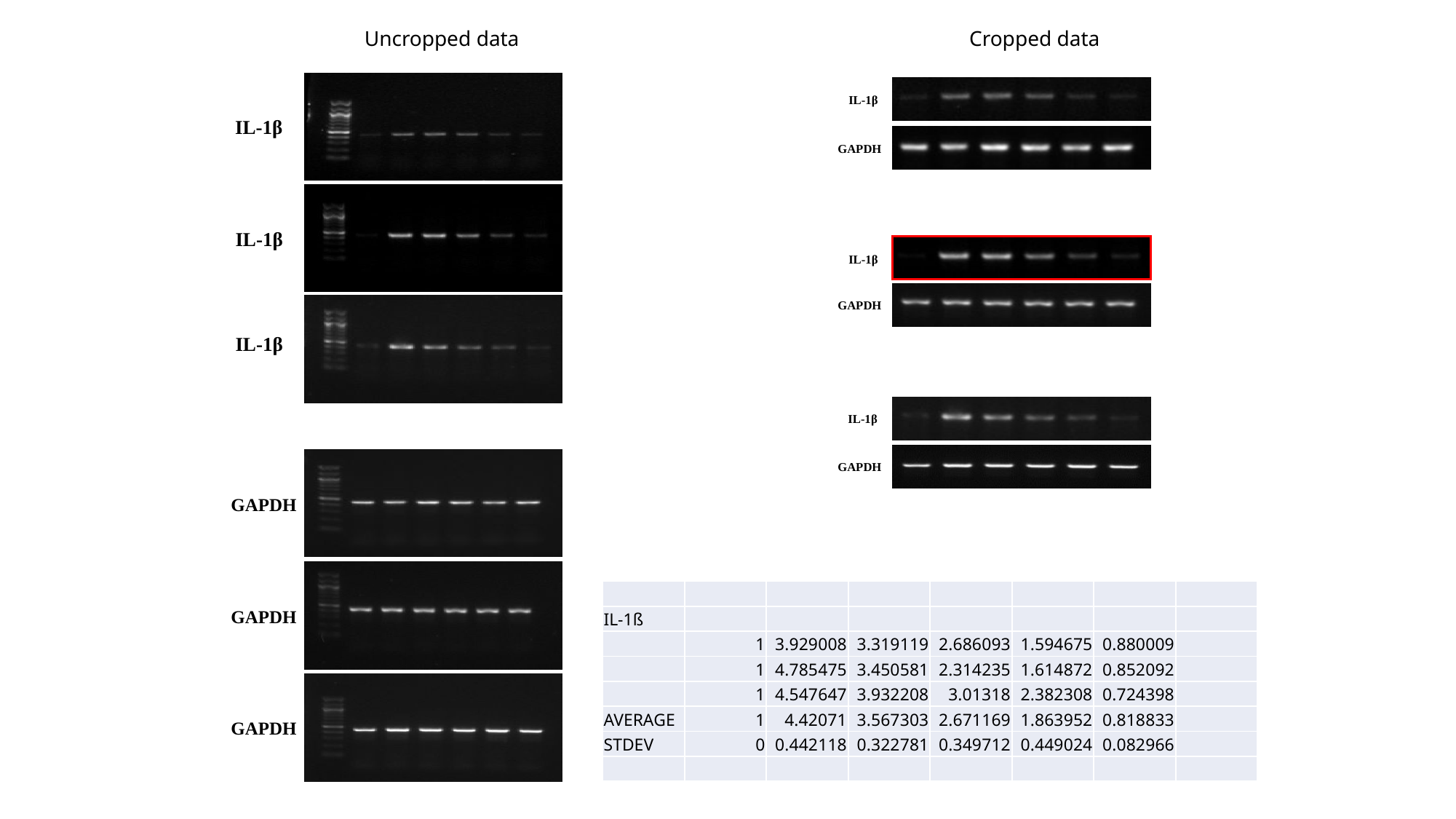

Uncropped data
Cropped data
IL-1β
IL-1β
GAPDH
IL-1β
IL-1β
GAPDH
IL-1β
IL-1β
GAPDH
GAPDH
| | | | | | | | |
| --- | --- | --- | --- | --- | --- | --- | --- |
| IL-1ß | | | | | | | |
| | 1 | 3.929008 | 3.319119 | 2.686093 | 1.594675 | 0.880009 | |
| | 1 | 4.785475 | 3.450581 | 2.314235 | 1.614872 | 0.852092 | |
| | 1 | 4.547647 | 3.932208 | 3.01318 | 2.382308 | 0.724398 | |
| AVERAGE | 1 | 4.42071 | 3.567303 | 2.671169 | 1.863952 | 0.818833 | |
| STDEV | 0 | 0.442118 | 0.322781 | 0.349712 | 0.449024 | 0.082966 | |
| | | | | | | | |
GAPDH
GAPDH

## Slide 19
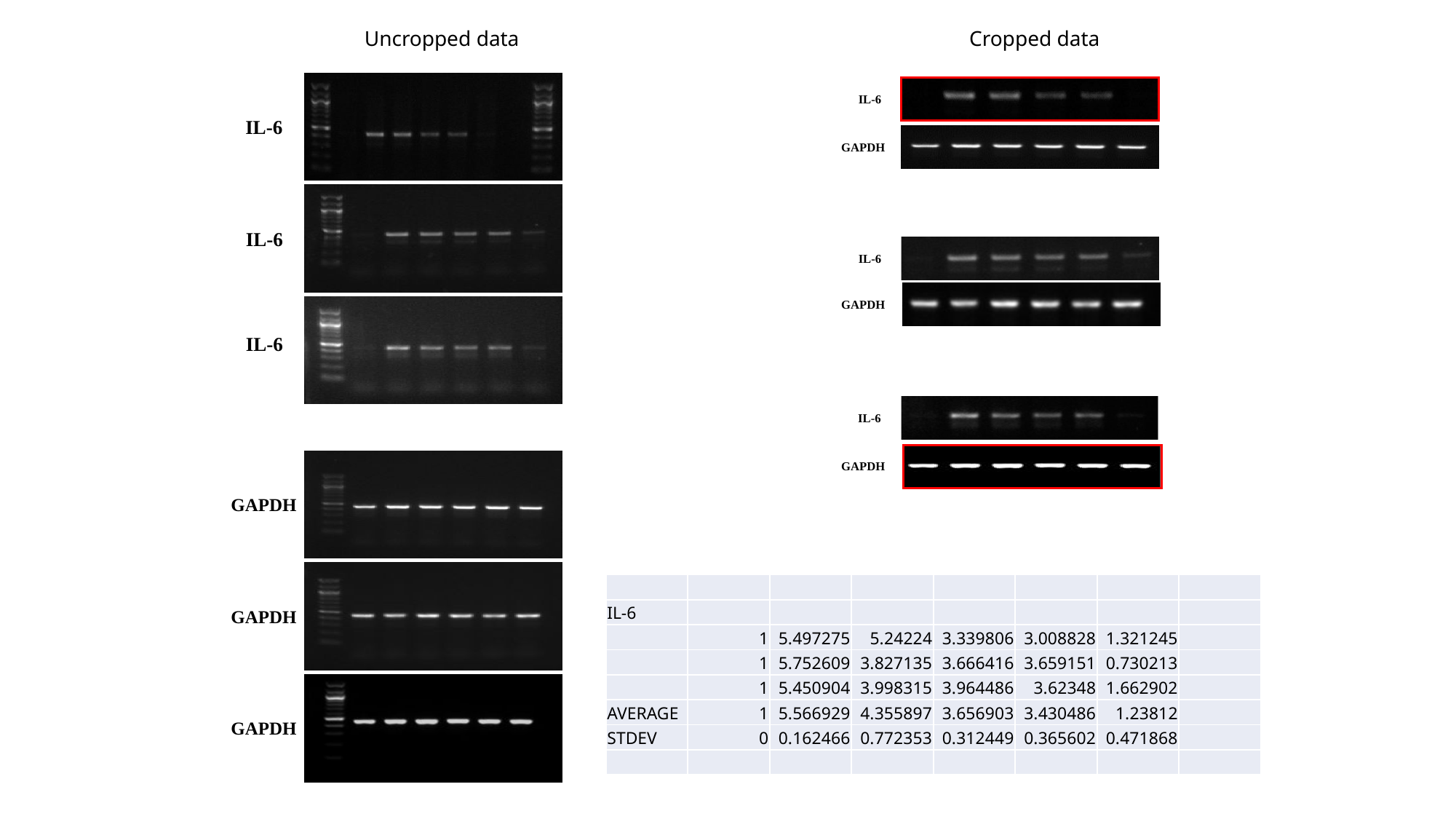

Uncropped data
Cropped data
IL-6
IL-6
GAPDH
IL-6
IL-6
GAPDH
IL-6
IL-6
GAPDH
GAPDH
| | | | | | | | |
| --- | --- | --- | --- | --- | --- | --- | --- |
| IL-6 | | | | | | | |
| | 1 | 5.497275 | 5.24224 | 3.339806 | 3.008828 | 1.321245 | |
| | 1 | 5.752609 | 3.827135 | 3.666416 | 3.659151 | 0.730213 | |
| | 1 | 5.450904 | 3.998315 | 3.964486 | 3.62348 | 1.662902 | |
| AVERAGE | 1 | 5.566929 | 4.355897 | 3.656903 | 3.430486 | 1.23812 | |
| STDEV | 0 | 0.162466 | 0.772353 | 0.312449 | 0.365602 | 0.471868 | |
| | | | | | | | |
GAPDH
GAPDH

## Slide 20
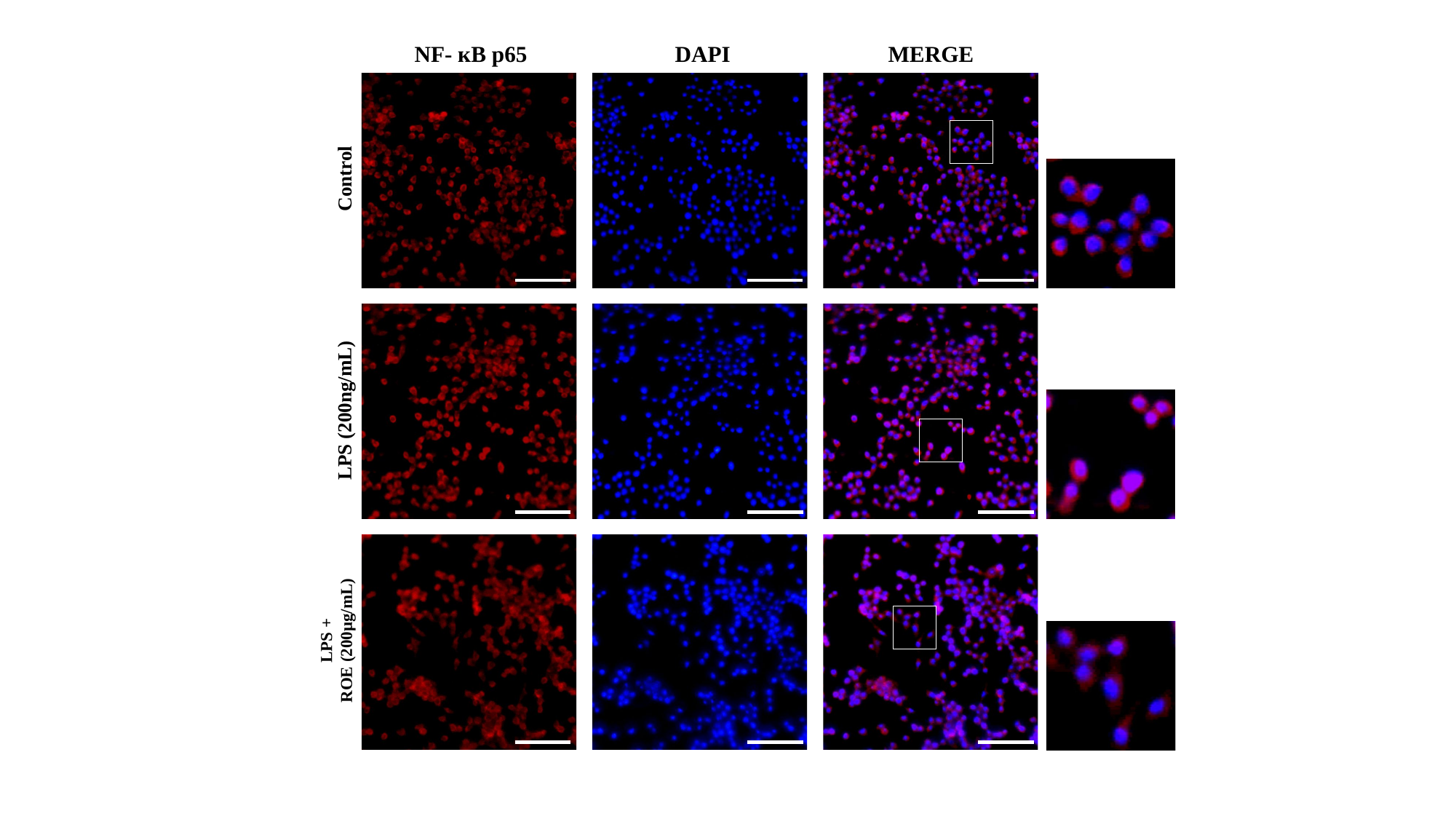

MERGE
DAPI
NF- кB p65
Control
LPS (200ng/mL)
LPS +
ROE (200μg/mL)

## Slide 21
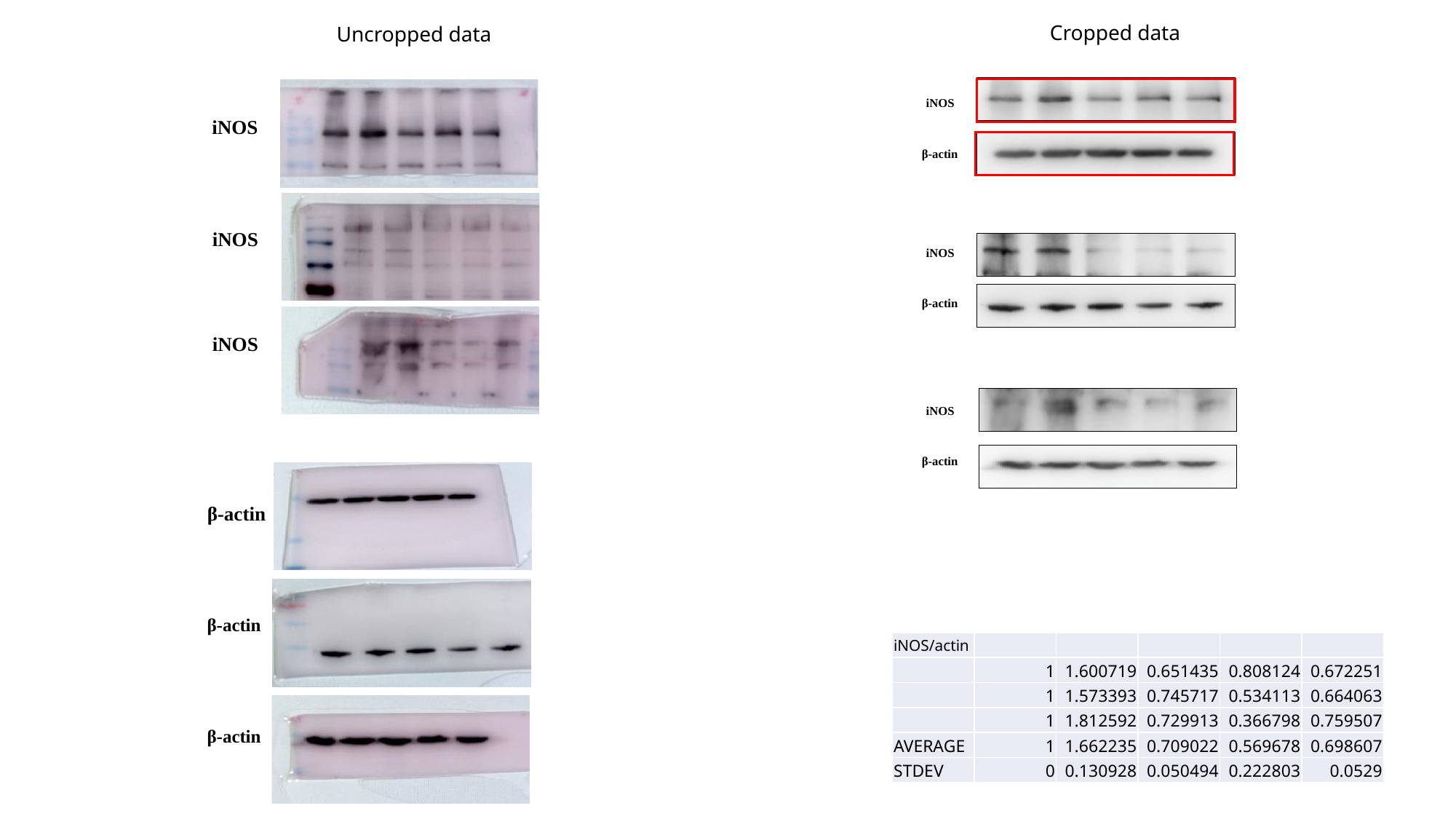

Cropped data
Uncropped data
iNOS
iNOS
β-actin
iNOS
iNOS
β-actin
iNOS
iNOS
β-actin
β-actin
β-actin
| iNOS/actin | | | | | |
| --- | --- | --- | --- | --- | --- |
| | 1 | 1.600719 | 0.651435 | 0.808124 | 0.672251 |
| | 1 | 1.573393 | 0.745717 | 0.534113 | 0.664063 |
| | 1 | 1.812592 | 0.729913 | 0.366798 | 0.759507 |
| AVERAGE | 1 | 1.662235 | 0.709022 | 0.569678 | 0.698607 |
| STDEV | 0 | 0.130928 | 0.050494 | 0.222803 | 0.0529 |
β-actin

## Slide 22
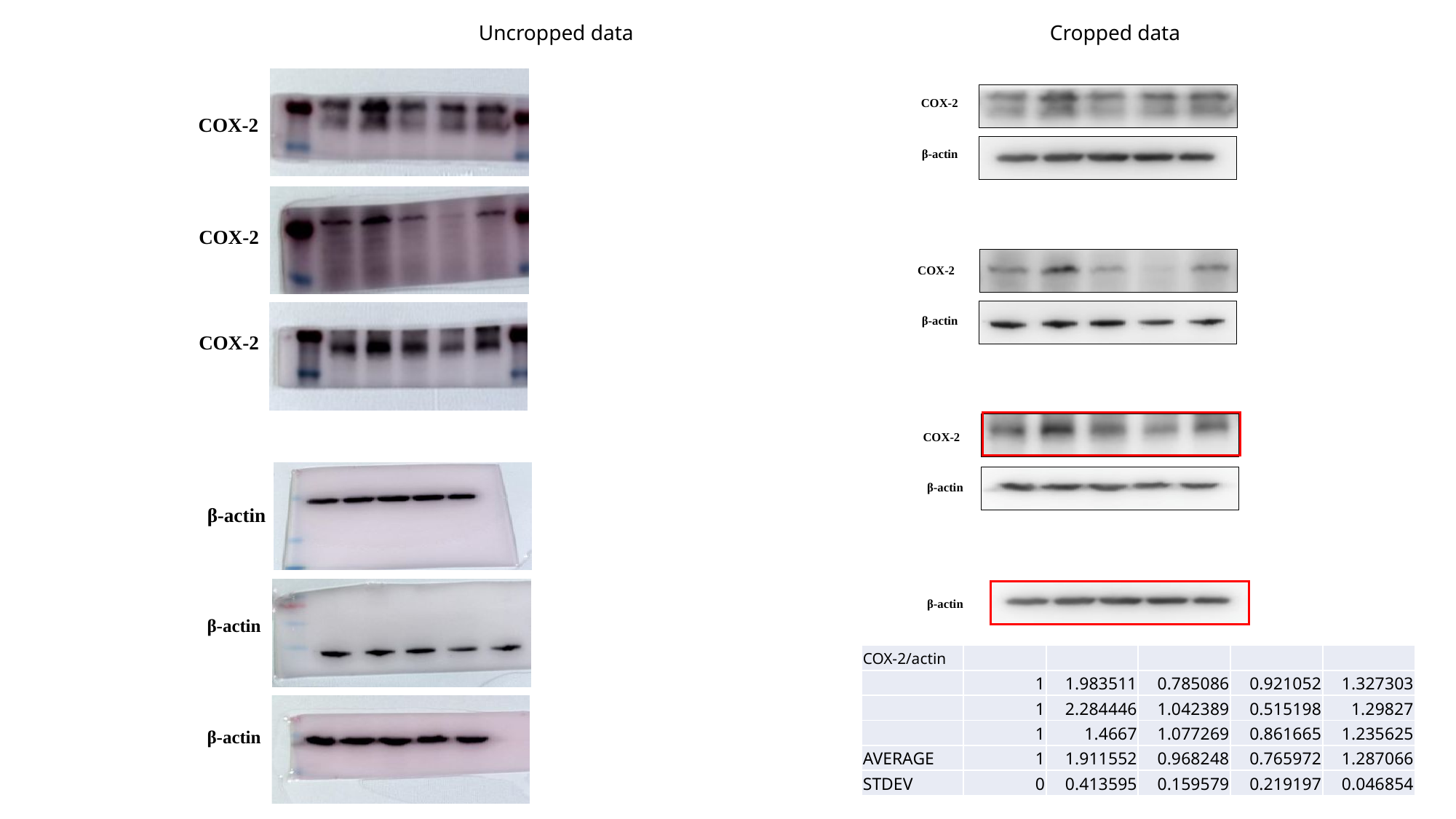

Uncropped data
Cropped data
COX-2
COX-2
β-actin
COX-2
COX-2
β-actin
COX-2
COX-2
β-actin
β-actin
β-actin
β-actin
| COX-2/actin | | | | | |
| --- | --- | --- | --- | --- | --- |
| | 1 | 1.983511 | 0.785086 | 0.921052 | 1.327303 |
| | 1 | 2.284446 | 1.042389 | 0.515198 | 1.29827 |
| | 1 | 1.4667 | 1.077269 | 0.861665 | 1.235625 |
| AVERAGE | 1 | 1.911552 | 0.968248 | 0.765972 | 1.287066 |
| STDEV | 0 | 0.413595 | 0.159579 | 0.219197 | 0.046854 |
β-actin

## Slide 23
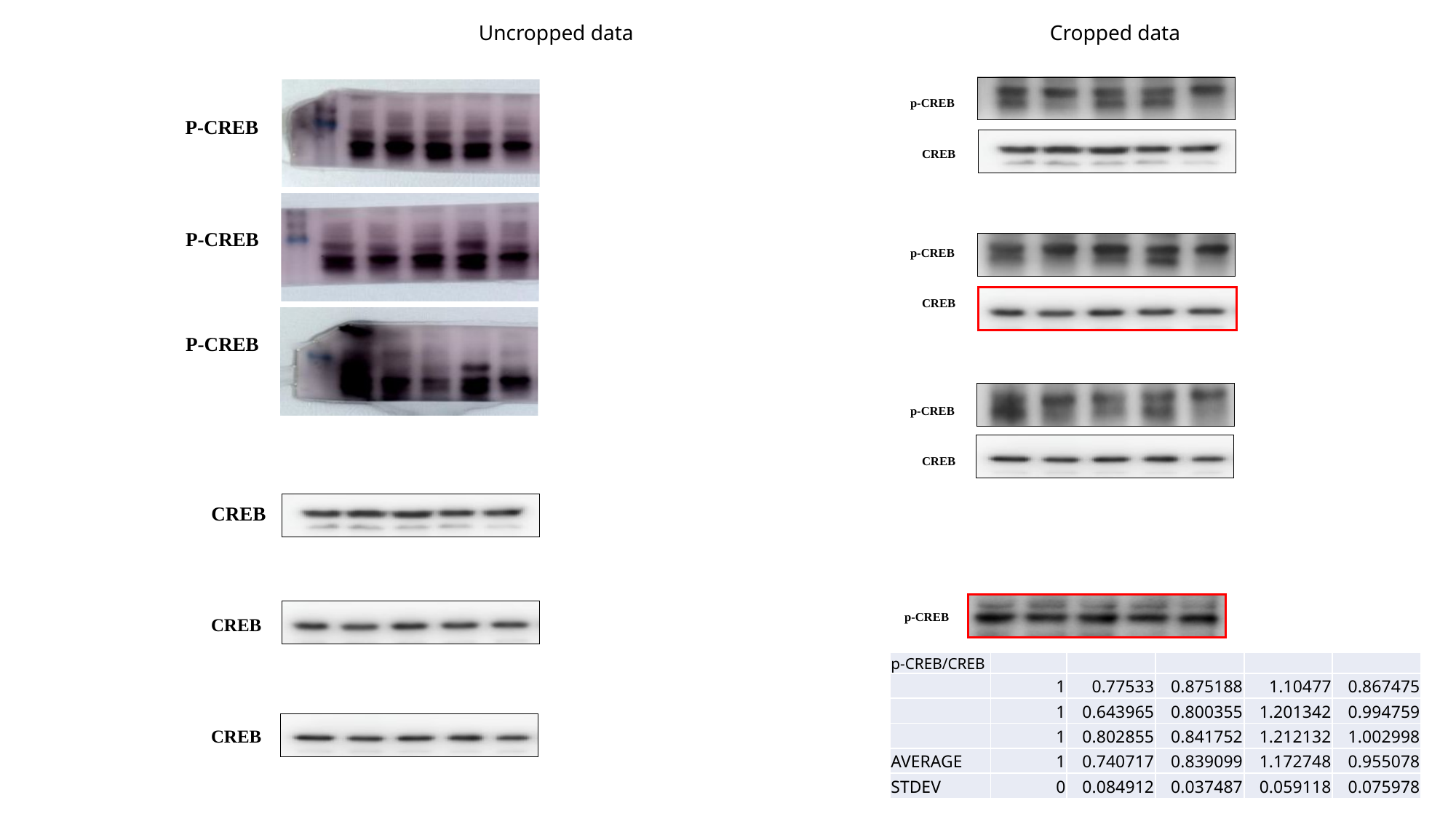

Uncropped data
Cropped data
p-CREB
P-CREB
CREB
P-CREB
p-CREB
CREB
P-CREB
p-CREB
CREB
CREB
p-CREB
CREB
| p-CREB/CREB | | | | | |
| --- | --- | --- | --- | --- | --- |
| | 1 | 0.77533 | 0.875188 | 1.10477 | 0.867475 |
| | 1 | 0.643965 | 0.800355 | 1.201342 | 0.994759 |
| | 1 | 0.802855 | 0.841752 | 1.212132 | 1.002998 |
| AVERAGE | 1 | 0.740717 | 0.839099 | 1.172748 | 0.955078 |
| STDEV | 0 | 0.084912 | 0.037487 | 0.059118 | 0.075978 |
CREB

## Slide 24
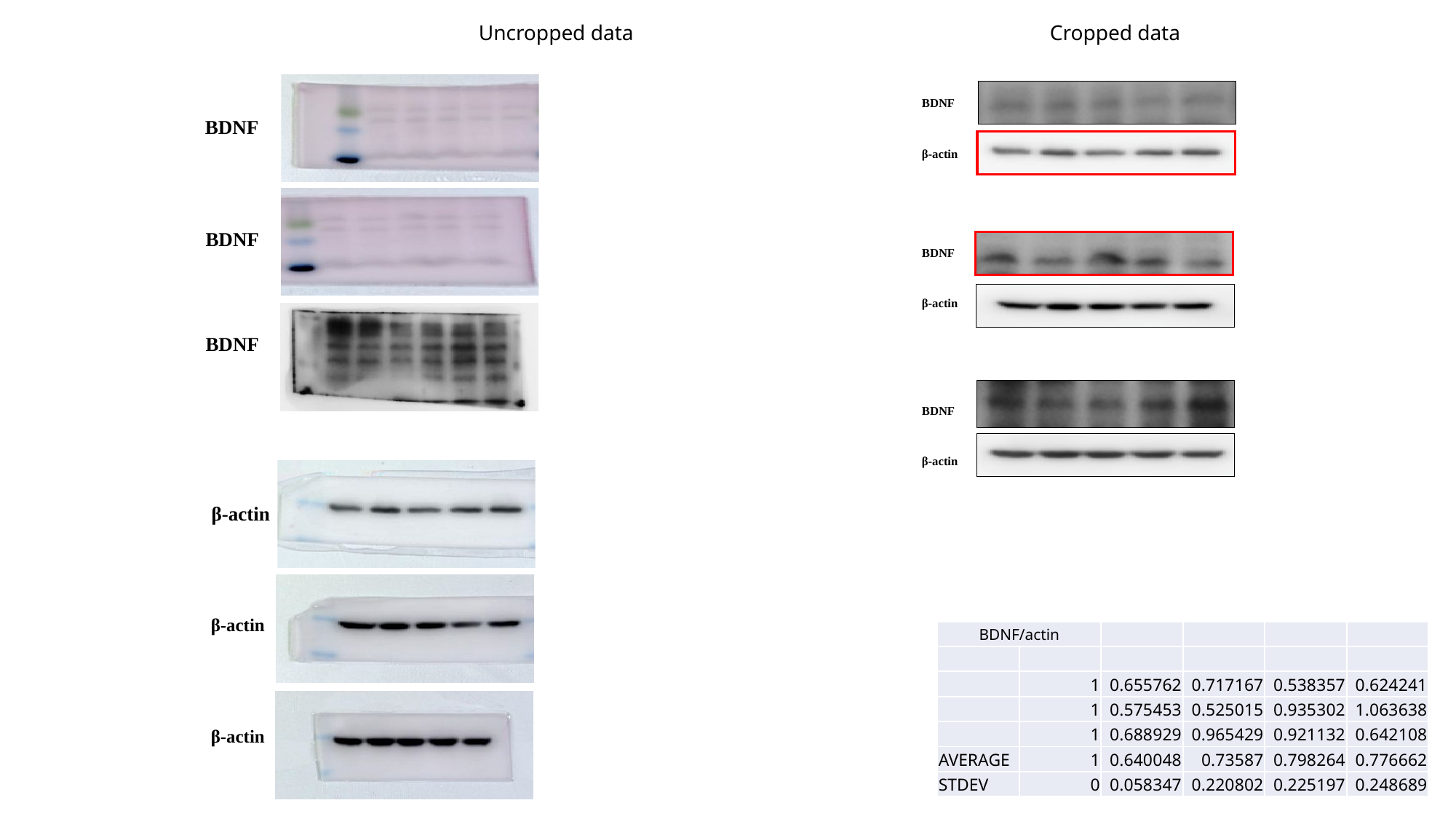

Uncropped data
Cropped data
BDNF
BDNF
β-actin
BDNF
BDNF
β-actin
BDNF
BDNF
β-actin
β-actin
β-actin
| BDNF/actin | | | | | |
| --- | --- | --- | --- | --- | --- |
| | | | | | |
| | 1 | 0.655762 | 0.717167 | 0.538357 | 0.624241 |
| | 1 | 0.575453 | 0.525015 | 0.935302 | 1.063638 |
| | 1 | 0.688929 | 0.965429 | 0.921132 | 0.642108 |
| AVERAGE | 1 | 0.640048 | 0.73587 | 0.798264 | 0.776662 |
| STDEV | 0 | 0.058347 | 0.220802 | 0.225197 | 0.248689 |
β-actin

## Slide 25
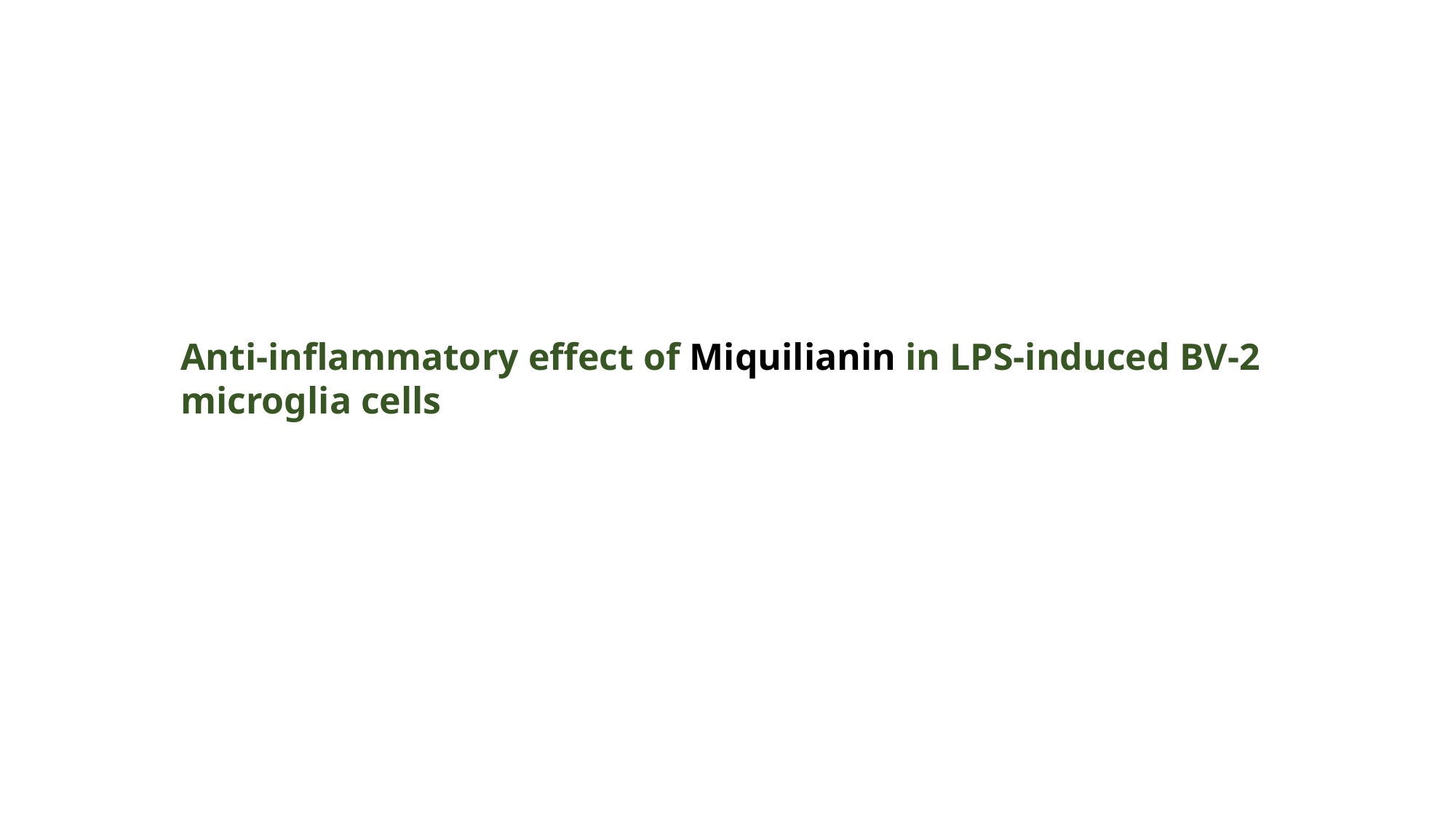

Anti-inflammatory effect of Miquilianin in LPS-induced BV-2 microglia cells

## Slide 26
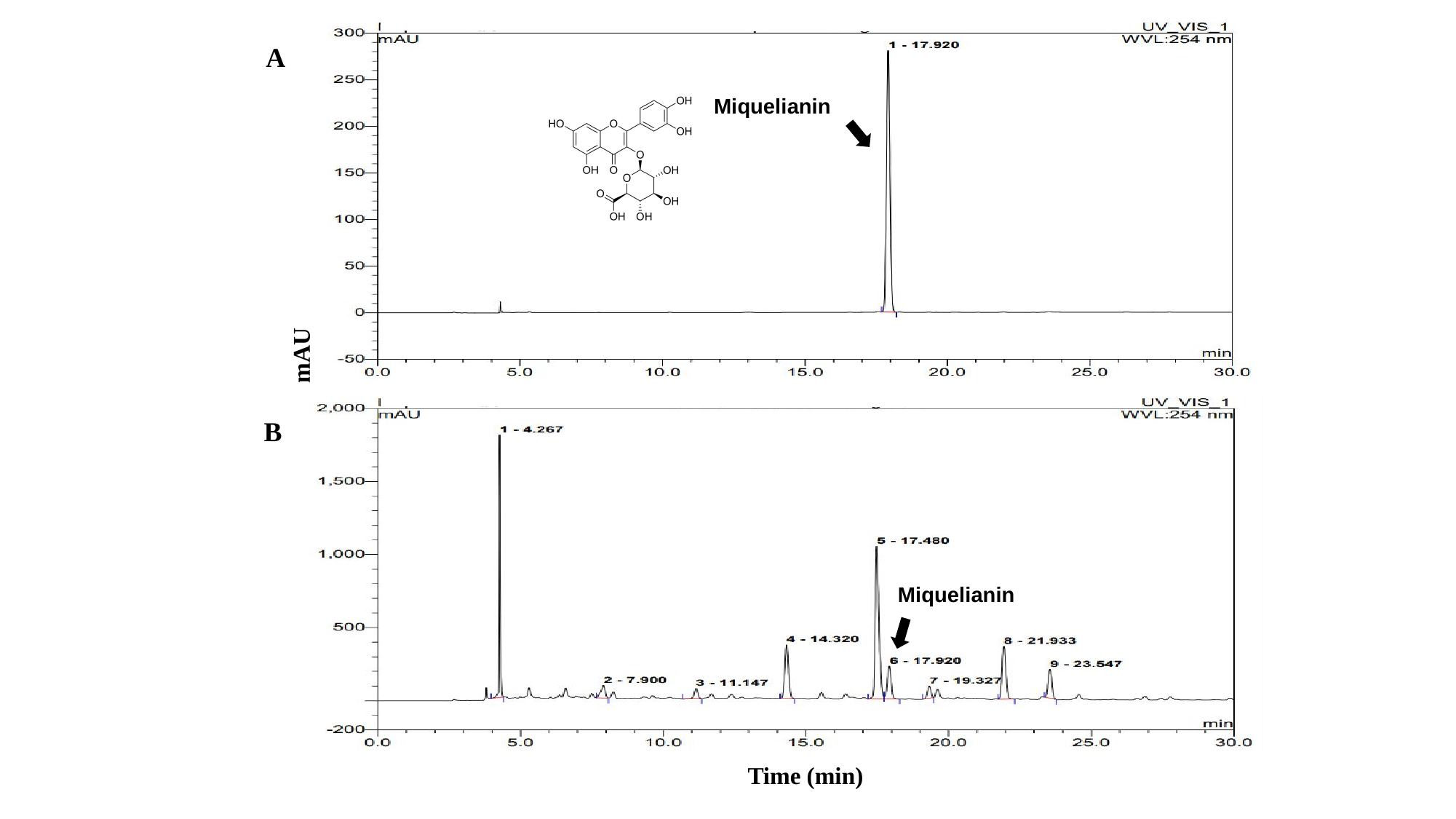

Miquelianin
mAU
Miquelianin
Time (min)
A
B

## Slide 27
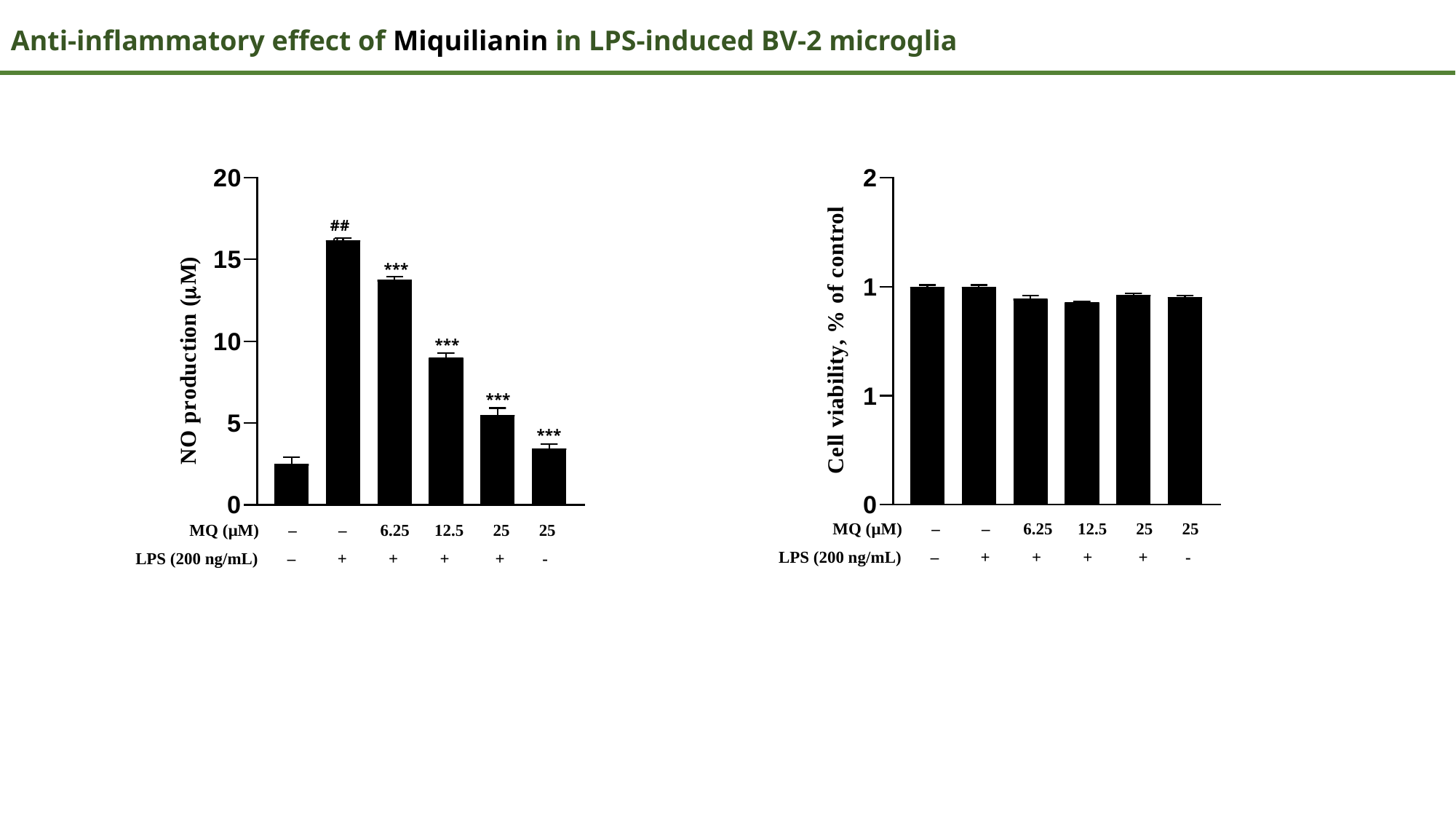

Anti-inflammatory effect of Miquilianin in LPS-induced BV-2 microglia
###
***
***
***
***
 MQ (μM) ‒ ‒ 6.25 12.5 25 25
 LPS (200 ng/mL) ‒ + + + + -
 MQ (μM) ‒ ‒ 6.25 12.5 25 25
 LPS (200 ng/mL) ‒ + + + + -

## Slide 28
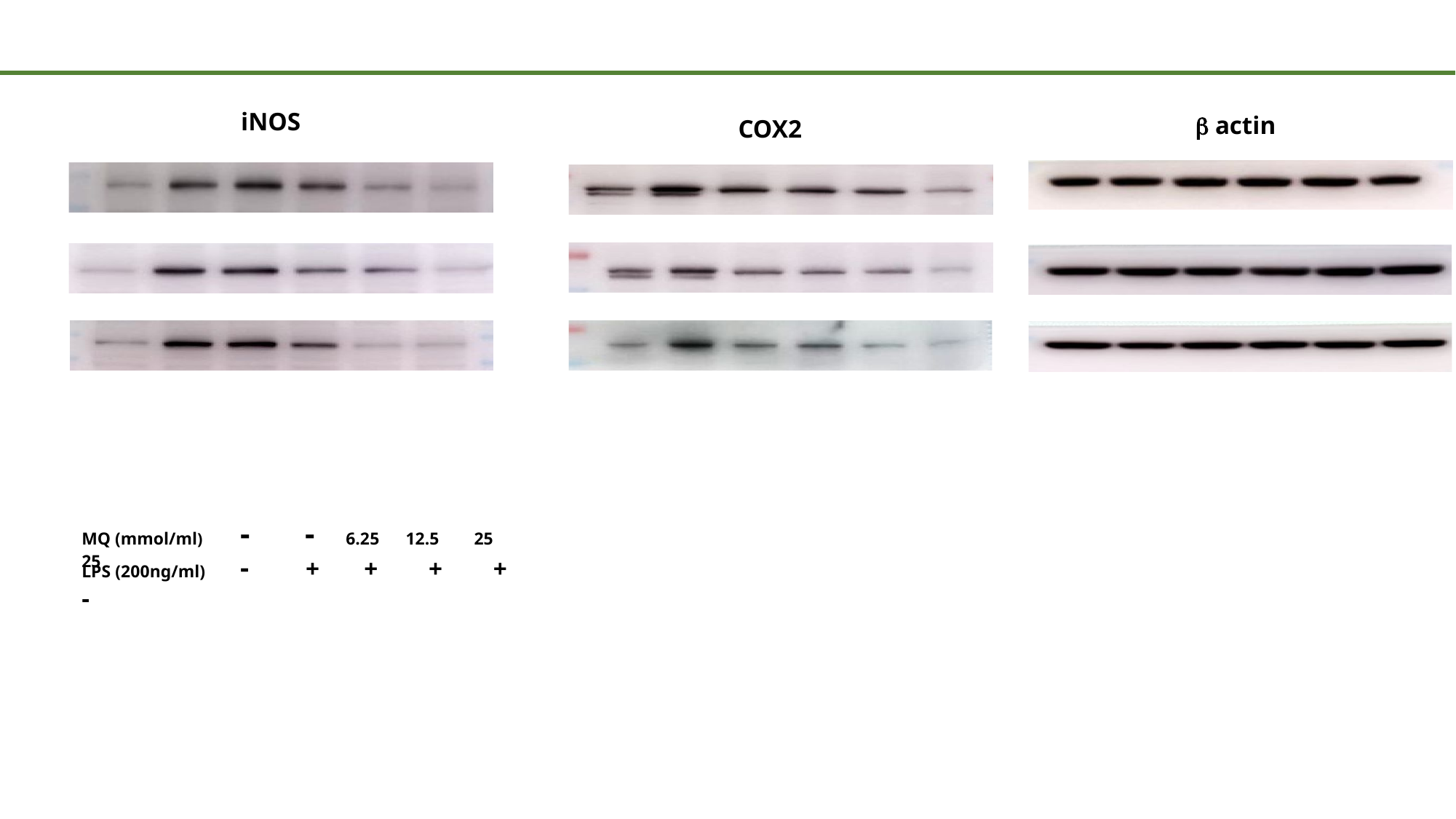

iNOS
 b actin
COX2
MQ (mmol/ml) - - 6.25 12.5 25 25
LPS (200ng/ml) - + + + + -

## Slide 29
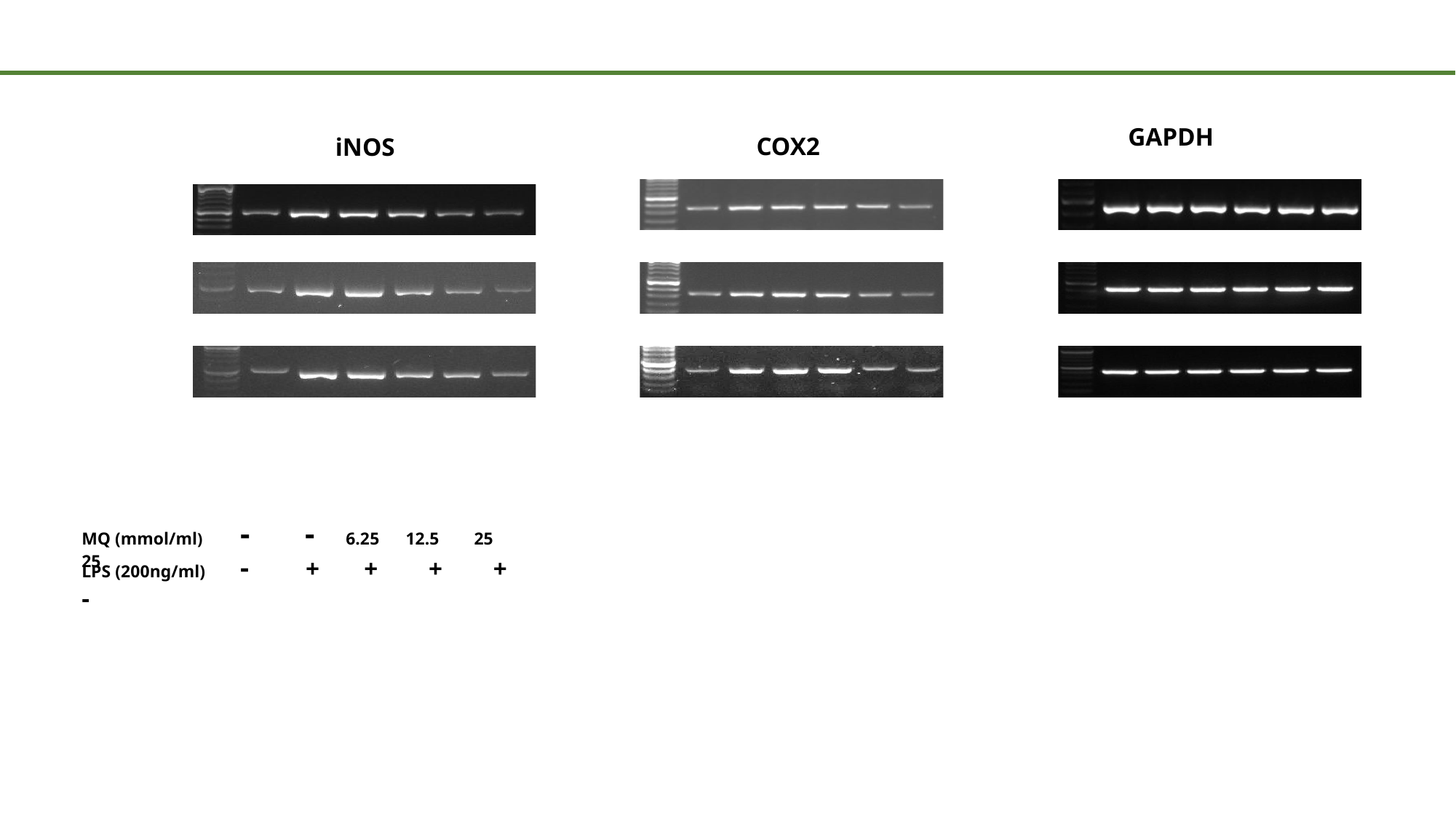

GAPDH
COX2
iNOS
MQ (mmol/ml) - - 6.25 12.5 25 25
LPS (200ng/ml) - + + + + -
